# The Spo13/Meikin pathway confines the onset of gamete differentiation to meiosis II in yeast

**DOI:** 10.15252/embj.2021109446

**Published:** 2022-01-13

**Authors:** Tugce Oz, Valentina Mengoli, Julie Rojas, Katarzyna Jonak, Marianne Braun, Ievgeniia Zagoriy, Wolfgang Zachariae

**Affiliations:** ^1^ Laboratory of Chromosome Biology Max Planck Institute of Biochemistry Martinsried Germany; ^2^ Max Planck Institute of Neurobiology Martinsried Germany; ^3^ Present address: Institute for Research in Biomedicine Università della Svizzera italiana Bellinzona Switzerland; ^4^ Present address: EMBL Heidelberg Heidelberg Germany

**Keywords:** gametogenesis, meiosis II, polo‐like kinase Cdc5, Spo13/Meikin, spore differentiation, Cell Cycle

## Abstract

Sexual reproduction requires genome haploidization by the two divisions of meiosis and a differentiation program to generate gametes. Here, we have investigated how sporulation, the yeast equivalent of gamete differentiation, is coordinated with progression through meiosis. Spore differentiation is initiated at metaphase II when a membrane‐nucleating structure, called the meiotic plaque, is assembled at the centrosome. While all components of this structure accumulate already at entry into meiosis I, they cannot assemble because centrosomes are occupied by Spc72, the receptor of the γ‐tubulin complex. Spc72 is removed from centrosomes by a pathway that depends on the polo‐like kinase Cdc5 and the meiosis‐specific kinase Ime2, which is unleashed by the degradation of Spo13/Meikin upon activation of the anaphase‐promoting complex at anaphase I. Meiotic plaques are finally assembled upon reactivation of Cdk1 at entry into metaphase II. This unblocking‐activation mechanism ensures that only single‐copy genomes are packaged into spores and might serve as a paradigm for the regulation of other meiosis II‐specific processes.

## Graphical abstract

Spore differentiation is coordinated with meiotic progression via APC/C‐mediated Spo13 degradation, which facilitates meiotic plaque formation by removal of Spc72.

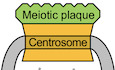

## Introduction

Delivery of the single‐copy genome into the zygote requires meiosis to be accompanied by differentiation into a gamete, a cell capable of engaging in fertilization. While meiosis follows a sequence of evolutionarily conserved steps, gamete differentiation is more diverse, creating spores, sperm, and oocytes. Nevertheless, conceptual similarities have been noted between sporulation in yeast and spermiogenesis in animals (White‐Cooper *et al*, 1998). A common feature is a transcriptional program, which provides the mRNAs that encode differentiation factors as well as cell cycle regulators. This program is activated before the first division by transcription factors such as Ndt80 in yeast, MYBL1 in mice, or a testis‐specific TFIID complex in flies (Lin *et al*, 1996; Chu & Herskowitz, 1998; Hiller *et al*, 2004; Bolcun‐Filas *et al*, 2011). Remarkably, work in flies has shown that spermiogenesis occurs even when the activation of Cdk1 is prevented and nuclear divisions are blocked (Alphey *et al*, 1992; Courtot *et al*, 1992; Sigrist *et al*, 1995). Thus, differentiation is thought to progress independently of cell cycle controls, raising the question of how and, indeed, whether the initiation of differentiation is coordinated with progression through meiosis. In most animals, spermiogenesis commences after meiosis and requires communication between spermatocytes and other cell types. In yeast, however, spore differentiation starts at meiosis II and is, therefore, more closely linked to meiosis. Furthermore, mutations in several cell cycle genes affect sporulation as well as nuclear division (Simchen, 1974), making yeast a promising model to study the coordination between the two processes.

Genome haploidization involves one round of DNA replication followed by two nuclear divisions, known as meiosis I and meiosis II (Petronczki *et al*, 2003). While DNA replication creates sister chromatids linked by cohesin complexes, subsequent recombination generates crossovers between homologous chromosomes, so that cohesins now connect all four chromatids. Nuclear divisions are initiated by the separase protease, which cleaves cohesin on chromosome arms at meiosis I and cohesin around centromeres at meiosis II. The stepwise cleavage of meiotic cohesin relies on its kleisin subunit Rec8, which is only recognized by separase upon phosphorylation (Katis *et al*, 2010). In yeast, the kinases Hrr25/CK1δ and Cdc7‐Dbf4 phosphorylate Rec8 on chromosome arms at meiosis I, while centromeric Rec8 is dephosphorylated and thereby protected from separase by the PP2A phosphatase bound to the shugoshin Sgo1 (Riedel *et al*, 2006). At anaphase II, centromeric Rec8 is deprotected through the removal of PP2A and subsequently phosphorylated by Hrr25 (Arguello‐Miranda *et al*, 2017).

Progression through meiosis I and meiosis II is governed by an oscillatory system with Cdk1 and the ubiquitin‐ligase APC/C^Cdc20^ at its center. Cdk1 bound to M‐phase cyclins (Clbs) induces spindle formation and is required for the activity of APC/C^Cdc20^. The activation of APC/C^Cdc20^ is restrained by the spindle assembly checkpoint (SAC), which is only silenced when all chromosomes are bioriented on the spindle (Lara‐Gonzalez *et al*, 2012). APC/C^Cdc20^‐dependent degradation of the separase‐inhibitor Pds1/securin triggers cohesin cleavage, while Clb degradation causes spindle disassembly and inactivation of APC/C^Cdc20^. This, in turn, allows re‐accumulation of Clbs and entry into meiosis II. In yeast, the Cdk1‐APC/C^Cdc20^ oscillator is initiated by the meiosis‐specific transcription factor Ndt80, which induces the expression of M‐phase regulators, such as Clbs and Cdc20, at entry into metaphase I (Chu & Herskowitz, 1998). How the oscillator is stopped is less clear but might involve activation of the meiosis‐specific APC/C^Ama1^ and degradation of Ndt80 at exit from meiosis II.

Additional mechanisms are required for ensuring the different outcomes of meiosis I and meiosis II. For instance, meiosis I segregates dyad chromosomes rather than sister chromatids because S‐phase kinases induce not only DNA replication but also recombination and sister kinetochore mono‐orientation before entry into metaphase I (Henderson *et al*, 2006; Matos *et al*, 2008). By contrast, it is unclear how meiosis II‐specific events are regulated. These include, for instance, the reduplication of spindle pole bodies (SPBs, the yeast centrosomes), the initiation of spore differentiation, translation of the Clb3 cyclin, redistribution of mitochondria, and deprotection of centromeric cohesin (Carlile & Amon, 2008; Neiman, 2011; Berchowitz *et al*, 2013; Agarwal *et al*, 2018; Sawyer *et al*, 2019; Mengoli *et al*, 2021). While these events depend on the Hrr25 or the Ime2 kinase, little is known about the regulation of these kinases (Benjamin *et al*, 2003; Petronczki *et al*, 2006). Here, we have investigated how the initiation of spore differentiation is confined to meiosis II, so that only single‐copy genomes are packaged into spores.

Spore formation requires the synthesis of a double‐membrane, called the prospore membrane (PSM), around each nucleus at meiosis II (Neiman, 2005, 2011). PSMs grow from a cap‐shaped structure, known as the meiotic plaque (MP), which forms on the cytoplasmic face of the SPB/centrosome. MPs assemble at metaphase II through the interdependent recruitment of three meiosis‐specific proteins, called Spo74, Mpc54, and Mpc70/Spo21 (Knop & Strasser, 2000; Bajgier *et al*, 2001; Nickas *et al*, 2003). MPs attract membrane vesicles from the cytoplasm, which fuse to form a pouch that expands over the emerging nuclei (Moreno‐Borchart & Knop, 2003). MP proteins disappear at late anaphase II when PSMs have fully enclosed each nucleus (Knop & Strasser, 2000). Finally, crosslinking of precursor molecules secreted into the PSM lumen creates the multi‐layered, mature spore wall (Coluccio *et al*, 2004).

Meiotic plaque proteins are expressed at metaphase I by the Ndt80 transcription factor (Chu & Herskowitz, 1998), but do not form MPs until metaphase II, when cells are poised to segregate sister centromeres. While this ensures that PSMs only enclose single‐copy genomes, the underlying mechanism is unclear. The finding that spore formation requires cell cycle regulators, such as Cdk1 and APC/C (Simchen, 1974), but not SPB reduplication or chromosome segregation (Buonomo *et al*, 2003; Marston *et al*, 2003), indicates that nuclear division and spore formation are under independent cell cycle control. However, it remains unclear how and, indeed, whether the cell cycle machinery directly controls MP assembly. Interestingly, MP assembly is preceded by the degradation of Spc72, the receptor of the γ‐tubulin complex (γ‐TuC) on the cytoplasmic face of the SPB, but its relevance to MP assembly is unclear (Knop & Schiebel, 1998; Knop & Strasser, 2000; Renicke *et al*, 2017). Any model attempting to explain how wild‐type cells confine MP assembly to metaphase II has to accommodate mutants that produce only two spores (Klapholz & Esposito, 1980a). Of particular interest in this regard is the *spo13Δ* mutant, which undergoes only a single round of APC/C activation and nuclear division (Shonn *et al*, 2002; Katis *et al*, 2004; Lee *et al*, 2004). Spo13 and its orthologues in fission yeast (Moa1) and mammals (Meikin) are meiosis I‐specific proteins that bind to polo‐like kinase (PLK) and promote sister kinetochore mono‐orientation at metaphase I (Matos *et al*, 2008; Kim *et al*, 2015).

We show here that spore differentiation is initiated by a three‐step sequence. While the Ndt80 transcription factor produces MP proteins at metaphase I, they cannot form MPs because the SPB outer layer is occupied by the γ‐TuC receptor Spc72. At anaphase I, APC/C^Cdc20^ unblocks SPBs by mediating the degradation of Spo13/Meikin and the inactivation of Cdk1‐Clb1. This enables the conserved kinases Cdc5/PLK and Ime2 to remove Spc72 from SPBs. MP assembly is finally triggered by the subsequent reappearance of Cdk1‐Clb activity. This three‐step sequence confines MP assembly to metaphase II and might serve as a paradigm for the regulation of other meiosis II‐specific processes.

## Results

### APC/C^Cdc20^ controls sporulation via Spo13/Meikin and the cyclin Clb1

**Figure 1:**
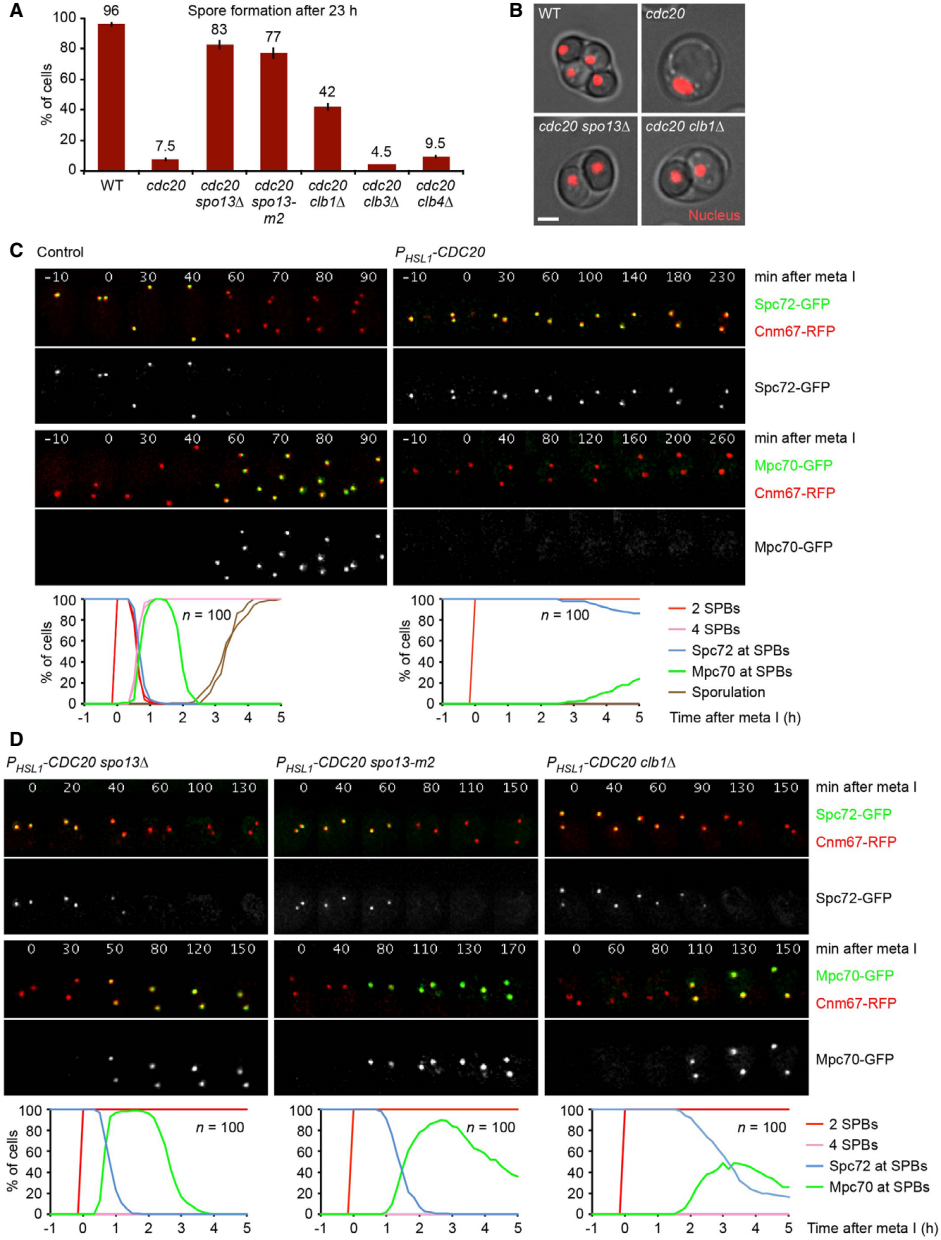
APC/C^Cdc20^ controls sporulation via Spo13/Meikin and the cyclin Clb1 Quantification of spore formation in the wild‐type (WT) and the indicated *P_HSL1_‐CDC20* strains at 23 h in sporulation medium (SPM). Error bars extend to data from two independent experiments (*n* = 200 cells per strain).Cells at 23 h in SPM. Nuclei are labeled with TetR‐RFP. Scale bar, 2 μm.Imaging of SPBs (Cnm67‐RFP) and Spc72‐GFP or Mpc70‐GFP in control and *P_HSL1_‐CDC20* cells. Top, time‐lapse series. Bottom, meiotic events were quantified in cells synchronized *in silico* to SPB separation at entry into metaphase I (*t* = 0). Graphs show overlays of *SPC72‐GFP* and *MPC70‐GFP* strains.Imaging of SPBs (Cnm67‐RFP) and Spc72‐GFP or Mpc70‐GFP in *P_HSL1_‐CDC20* strains containing the indicated mutations. Meiotic events were analyzed as in (C). Data information: Data are representative of four (C) or two (D) independent experiments.

Depleting meiotic cells of the APC/C activator Cdc20 prevents spore formation as well as nuclear division, indicating that APC/C^Cdc20^ has a role in coordinating these processes. *cdc20* mutants might accumulate inhibitors of sporulation, and deletion of such proteins might restore sporulation in the mutant. Indeed, deletion of *SPO13*, which encodes a meiosis I‐specific substrate of APC/C^Cdc20^, has been reported to cause sporulation in cells expressing *CDC20* from the mitosis‐specific *CLB2* promoter (Katis *et al*, 2004). We confirmed this finding in cells expressing *CDC20* from the more tightly controlled *HSL1* promoter. Whereas only few *P_HSL1_‐CDC20* cells (8%) produce a single spore, *P_HSL1_‐CDC20 spo13Δ* double mutants undergo nuclear division and form two spores with high efficiency (83%; Fig 1A and B). The *spo13‐m2* mutation, which reduces Spo13's affinity for the PLK Cdc5 (Matos *et al*, 2008), has a similar effect. In addition, deletion of *CLB1*, encoding an M‐phase cyclin, causes *P_HSL1_‐CDC20* cells to undergo nuclear division and spore formation with a frequency of 42% (Fig 1A and B). By contrast, no effect was observed in cells lacking the cyclin Clb3 or Clb4. Thus, APC/C^Cdc20^ might control the spore formation pathway through the degradation of Spo13 and Clb1.

Sporulation depends on the synthesis of MP proteins at metaphase I and their assembly into MPs on the cytoplasmic face of the SPB at metaphase II. MP assembly might be controlled by the γ‐TuC receptor Spc72, which is degraded and removed from SPBs as cells approach meiosis II (Knop & Strasser, 2000; Renicke *et al*, 2017). Thus, we investigated the role of APC/C^Cdc20^ in switching SPBs from Spc72 binding to MP assembly. Imaging of control cells shows that Spc72‐GFP disappears from SPBs (labeled with Cnm67‐RFP) at late anaphase I. Mpc70‐GFP appears on SPBs ~ 10 min later, when SPBs reduplicate at entry into metaphase II (Fig 1C, left). The tight correlation between Spc72 removal and Mpc70 loading at SPBs was confirmed in cells containing fluorophore‐tagged versions of both proteins (Appendix Fig S1A and B). Spc72 removal and Mpc70 loading are blocked in *P_HSL1_‐CDC20* cells (Fig 1C, right). These data support the idea that APC/C^Cdc20^ activity is required for the removal of Spc72 from SPBs, which might be a prerequisite for MP assembly.

Next, we investigated the roles of Spo13 and Clb1 in the exchange of Spc72 for Mpc70. In wild‐type cells, the exchange occurs after the degradation of Pds1 at anaphase I (Appendix Fig S1A). In *spo13Δ* mutants, Pds1 degradation is delayed in a SAC‐dependent manner, and SPB reduplication does not occur (Shonn *et al*, 2002). Nevertheless, *spo13Δ* cells exchange Spc72 for Mpc70 slightly earlier than control cells. As a result, the exchange occurs before the degradation of Pds1, that is, at metaphase I (Appendix Fig S1A). This indicates that the *SPO13* deletion uncouples Spc72 removal from APC/C^Cdc20^ activity, while the strict correlation between Spc72 removal and Mpc70 recruitment is preserved (Appendix Fig S1B). Indeed, *P_HSL1_‐CDC20 spo13Δ* double mutants remove Spc72 from and recruit Mpc70 to SPBs with near‐normal kinetics (Fig 1D, left). SPBs also exchange Spc72 for Mpc70, albeit with slower kinetics, in *P_HSL1_‐CDC20* cells that contain the *spo13‐m2* mutation or the *CLB1* deletion (Fig 1D, middle and right).

### Spc72's presence at SPBs during metaphase I requires Spo13 and Cdk1‐Clb1

**Figure EV1:**
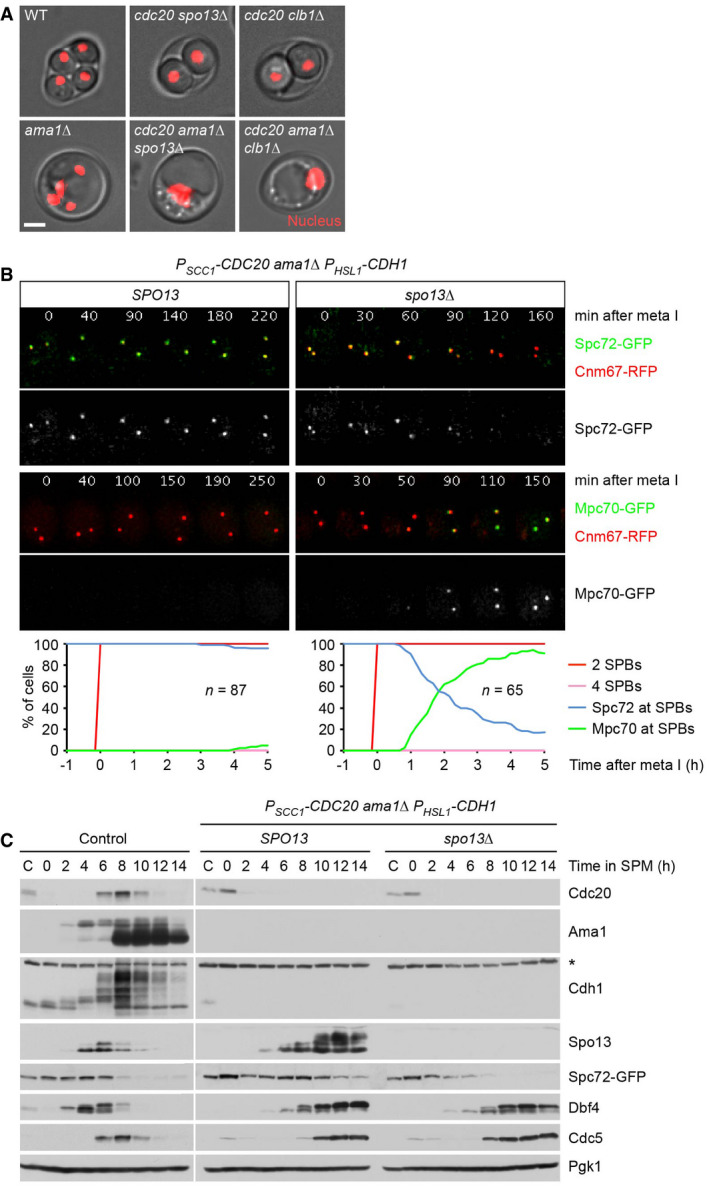
Analysis of Spo13 function in cells lacking APC/C activators Deletion of *AMA1* blocks spore formation and reduces nuclear division (to < 5%) in *P_HSL1_‐CDC20 spo13Δ* and *P_HSL1_‐CDC20 clb1Δ* cells. Images were taken at 23 h in SPM. Nuclei are labeled with TetR‐RFP. Scale bar, 2 μm.Imaging of SPBs (Cnm67‐RFP) and Spc72‐GFP or Mpc70‐GFP in *SPO13* and *spo13Δ* cells lacking all three APC/C activators. Top, time‐lapse series. Bottom, meiotic events were quantified in cells synchronized *in silico* to SPB separation at entry into metaphase I (*t* = 0). Graphs show overlays of *SPC72‐GFP* and *MPC70‐GFP* strains. Data are representative of three independent experiments.Immunoblot detection of proteins in whole‐cell extracts from control cells and from *SPO13* and *spo13Δ* cells lacking all three APC/C activators. C, sample from proliferating cells. The asterisk marks a non‐specific band. Source data are available online for this figure.

**Figure 2:**
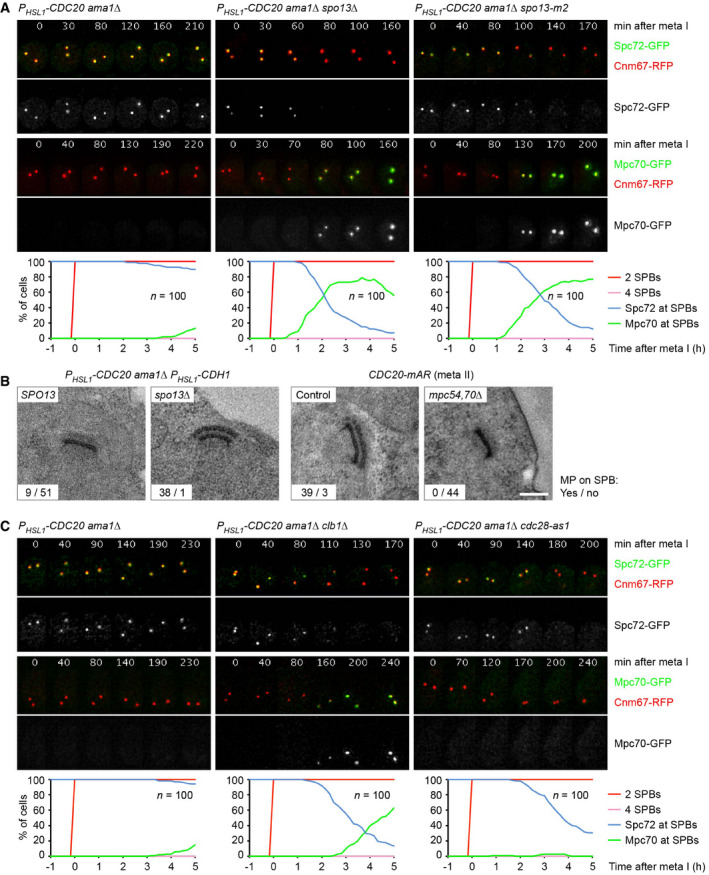
Inactivation of Spo13 or Cdk1‐Clb1 causes exchange of Spc72 for Mp70 in metaphase I‐arrested cells Imaging of SPBs (Cnm67‐RFP) and Spc72‐GFP or Mpc70‐GFP in *P_HSL1_‐CDC20 ama1Δ* control cells and *spo13* mutants. Top, time‐lapse series. Bottom, meiotic events were quantified in cells synchronized *in silico* to SPB separation at entry into metaphase I (*t* = 0). Graphs show overlays of *SPC72‐GFP* and *MPC70‐GFP* strains.TEM analysis of SPBs. Left, SPBs in *SPO13* and *spo13Δ* cells lacking all APC/C activators. TEM samples were collected at 12 h in SPM. The *spo13Δ* mutation causes MP formation at metaphase I (*P* < 0.0001, Fisher's exact test). Right, SPBs at metaphase II. *CDC20‐mAR* and *CDC20‐mAR mpc54Δ mpc70Δ* cells were synchronized by arrest/release at metaphase I, and TEM samples were collected at metaphase II (80 min after release). MP formation is blocked in the absence of Mpc54 and Mpc70 (*P* < 0.0001, Fisher's exact test). Scale bar, 0.2 μm.Imaging of SPBs (Cnm67‐RFP) and Spc72‐GFP or Mpc70‐GFP in *P_HSL1_‐CDC20 ama1Δ* control cells and cells containing *clb1Δ* or *cdc28‐as1*, which have been treated with 1NM‐PP1 at metaphase I (8 h in SPM). Meiotic events (bottom) were analyzed as in (A). Data information: Data in (A) and (C) are representative of two independent experiments.

*P_HSL1_‐CDC20 spo13Δ* cells undergo nuclear division as well as spore formation. This nuclear division is blocked upon deletion of *AMA1* (Fig EV1A), suggesting that Spo13 has a role in preventing APC/C^Ama1^ from promoting nuclear division when APC/C^Cdc20^ is inhibited or absent. The *AMA1* deletion also precludes spore formation because it prevents the closure of the PSM into a sphere around each meiosis II nucleus (Diamond *et al*, 2009). However, the absence of Ama1 does not prevent the exchange of Spc72 for Mpc70 at SPBs (Fig 2A). The *SPO13* deletion causes Spc72 removal and Mpc70 recruitment even in cells that lack all three APC/C activators (Cdc20, Ama1, and Cdh1; Fig EV1B). Transmission electron microscopy (TEM) showed that these metaphase I‐arrested cells produce MPs, which are indistinguishable from those normally observed at metaphase II (Fig 2B). Immunoblotting of protein extracts revealed that Spc72's removal from SPBs is accompanied by its degradation (Fig EV1C), demonstrating that APC/C^Cdc20^ controls Spc72 stability indirectly, by mediating the degradation of Spo13. While Spo13 is considered a bona fide APC/C substrate (Sullivan & Morgan, 2007), recent work on Meikin, the mammalian orthologue of Spo13, suggested an alternative mechanism for APC/C‐dependent inactivation, namely cleavage by separase (Maier *et al*, 2021). Appendix Fig S2 shows, however, that Spo13 is not affected by the activation of separase in metaphase I‐arrested cells.

The *CLB1* deletion resembles the *SPO13* deletion in several aspects: First, it causes nuclear division and spore formation in cells depleted of Cdc20 (Fig 1A and B). Second, this nuclear division is blocked upon deletion of *AMA1* (Fig EV1A). Third, the *CLB1* deletion causes the SPBs of *P_HSL1_‐CDC20 ama1Δ* cells to exchange Spc72 for Mpc70 (Fig 2C, middle). By contrast, deletion of *CLB3* or *CLB4* does not elicit Spc72 removal (Appendix Fig S3), which is consistent with the absence of sporulation in the corresponding *P_HSL1_‐CDC20* mutants (Fig 1A). To investigate whether Clb1 exerts its function through Cdk1 activity, we used *cdc28‐as1* cells whose Cdk1 can be inhibited by the ATP‐analogue 1NM‐PP1 (Bishop *et al*, 2000). Thus, we allowed *P_HSL1_‐CDC20 ama1Δ cdc28‐as1* cells to progress to metaphase I and then added 1NM‐PP1. Indeed, inhibition of Cdk1 results in Spc72's removal from SPBs (Fig 2C, right). These data suggest that Cdk1‐Clb1 activity is required for Spc72's persistence at SPBs during metaphase I. Interestingly, in contrast to the *SPO13* and the *CLB1* deletion, Spc72 removal triggered by Cdk1 inhibition does not elicit recruitment of Mpc70 to SPBs (Fig 2C, right), indicating that Cdk1 activity has an additional role in MP assembly. Taken together, our data suggest that Spo13 and Cdk1‐Clb1 activity are both required for Spc72's presence at SPBs during metaphase I and that inactivation of either one of them is sufficient to elicit Spc72's removal.

### Spc72 prevents premature MP assembly at metaphase I

**Figure 3:**
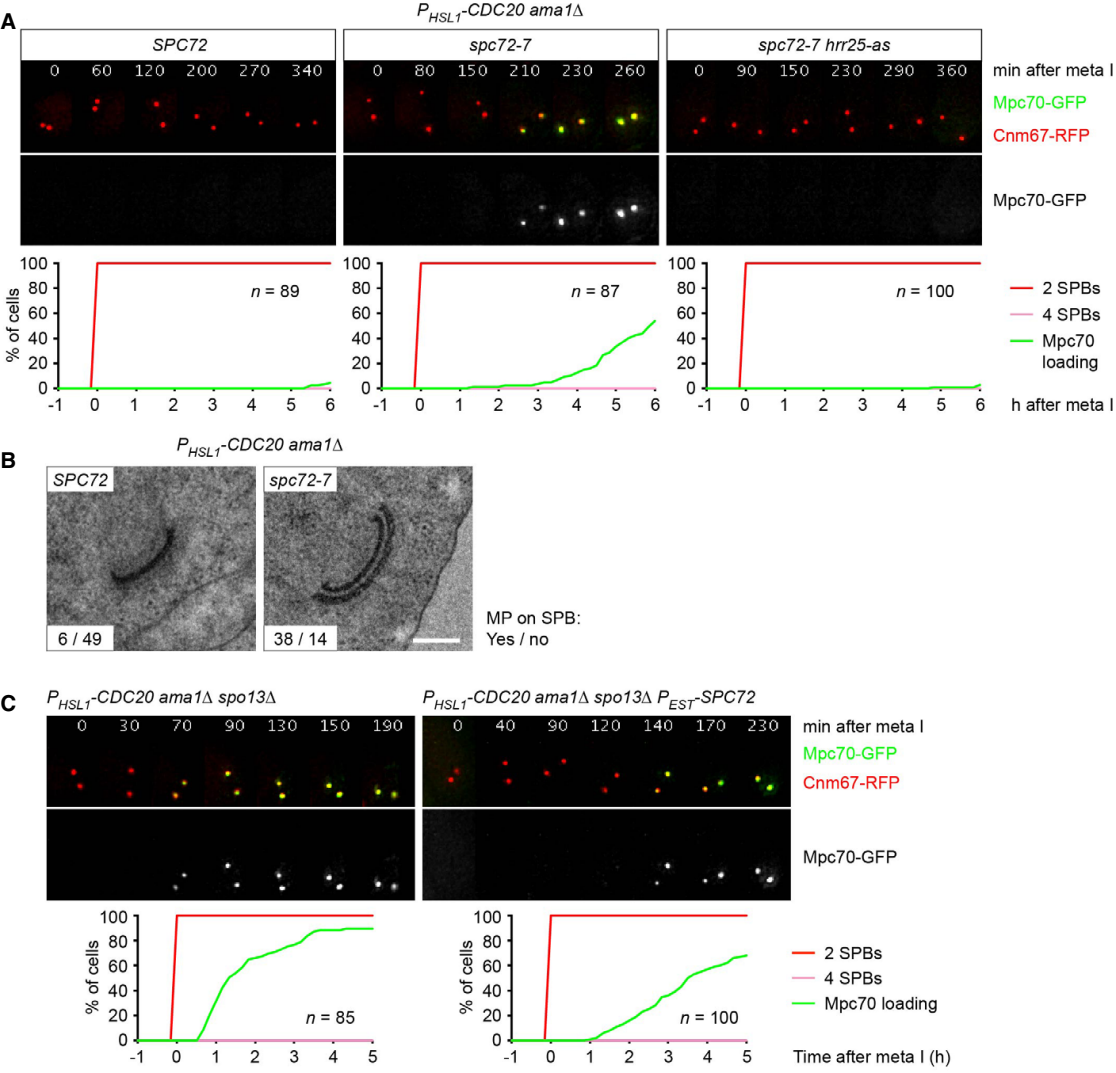
Spc72 prevents premature MP assembly at metaphase I Imaging of SPBs (Cnm67‐RFP) and Mpc70‐GFP in *P_HSL1_‐CDC20 ama1Δ* cells containing *SPC72*, *spc72‐7*, or *spc72‐7* plus *hrr25‐as*. Cells were shifted from 24 to 36°C at 4 h in SPM and treated with 1NM‐PP1 to inhibit Hrr25‐as. Top, time‐lapse series. Bottom, Mpc70 loading to SPBs was quantified in cells synchronized *in silico* to SPB separation at entry into metaphase I (*t* = 0).TEM analysis of SPBs. *P_HSL1_‐CDC20 ama1Δ* cells containing *SPC72* or *spc72‐7* were shifted from 24 to 36°C at 4 h in SPM. TEM samples were collected at 12 h in SPM. Spc72 inactivation causes MP formation at metaphase I (*P* < 0.0001, Fisher's exact test). Scale bar, 0.2 μm.Imaging of SPBs (Cnm67‐RFP) and Mpc70‐GFP in *P_HSL1_‐CDC20 ama1Δ spo13Δ* control cells and cells expressing an additional copy of *SPC72* from the *P_EST_* promoter at 4 h in SPM. Mpc70 loading (bottom) was analyzed as in (A). *P_EST_‐SPC72* expression delays the time of Mpc70 loading by 98 min (95% CI, 67–129; *P* < 0.0001; Welch's *t*‐test). Data information: Data in (A) and (C) are representative of three independent experiments.

To more directly test whether Spc72 prevents MP assembly at metaphase I, we introduced the temperature‐sensitive (ts) *spc72‐7* allele (Knop & Schiebel, 1998) into *P_HSL1_‐CDC20 ama1Δ* cells. Shifting metaphase I‐arrested cells to 36°C causes accumulation of Mpc70 at SPBs in the *spc72‐7* mutant but not in the *SPC72* control strain (Fig 3A). This Mpc70 recruitment depends on Hrr25 activity (Fig 3A, right), similar to the recruitment that occurs at metaphase II in the wild type (Arguello‐Miranda *et al*, 2017). Indeed, *spc72‐7* cells assemble MPs, which closely resemble those normally produced at metaphase II (Fig 3B). These data suggest that Spc72 is required for suppressing premature MP assembly at metaphase I. Consistent with this idea, expression of an additional copy of *SPC72* delays the recruitment of Mpc70 observed in *P_HSL1_‐CDC20 ama1Δ spo13Δ* cells by 98 min (Fig 3C). However, Mpc70 recruitment is slower in *spc72‐7* than in *spo13Δ* cells, raising the possibility that Spc72 is not the only means by which Spo13 inhibits MP assembly at metaphase I. We shall return to this issue further below.

### Co‐expression of stabilized Spo13 and Clb1 delays Spc72 removal from SPBs

**Figure EV2:**
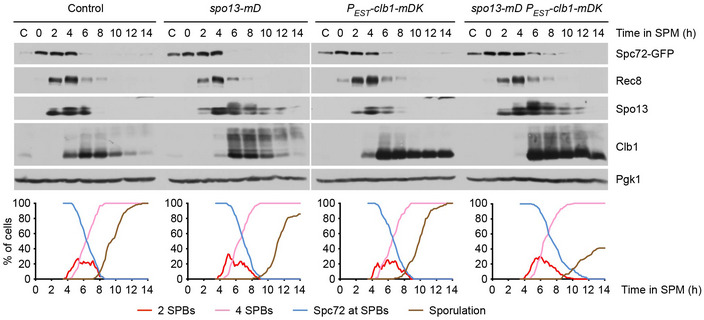
Protein levels in strains expressing non‐degradable Spo13 and/or Clb1 Analysis of meiosis in cells containing Spo13‐mD and/or expressing Clb1‐mDK from the *P_EST_* promoter. Estradiol was added at 4 h in SPM to induce *P_EST_‐clb1‐mDK*. Top, immunoblot detection of proteins in whole‐cell extracts. C, sample from proliferating cells. Bottom, culture aliquots were subjected to live‐imaging of SPBs (Cnm67‐RFP) and Spc72‐GFP. Graphs show quantification of meiotic events (*n* = 100 cells per strain). Source data are available online for this figure.

**Figure 4:**
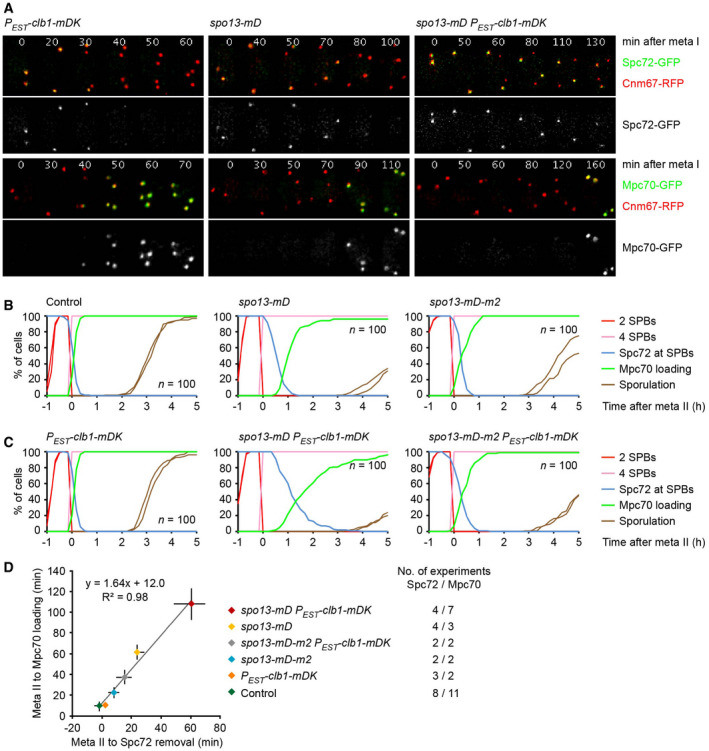
Co‐expression of stabilized Spo13 and Clb1 delays Spc72 removal and Mpc70 recruitment at SPBs A–DImaging of SPBs (Cnm67‐RFP) and Spc72‐GFP or Mpc70‐GFP in cells containing different *SPO13* alleles and/or *P_EST_‐clb1‐mDK*, which was induced with estradiol at 4 h in SPM. (A) Representative time‐lapse series. (B) Analysis of cells containing wild‐type Spo13, Spo13‐mD, or Spo13‐mD‐m2. Meiotic events were quantified in cells synchronized *in silico* to SPB reduplication at entry into metaphase II (*t* = 0). Graphs show overlays of *SPC72‐GFP* and *MPC70‐GFP* strains. *spo13‐mD* delays Spc72 removal and Mpc70 loading by 30 min (95% CI, 26–34; *P* < 0.0001) and 60 min (95% CI, 55–66; *P* < 0.0001; Welch's *t*‐test), respectively. (C) Analysis of cells expressing Clb1‐mDK and different versions of Spo13. Meiotic events were quantified as in (B). *spo13‐mD* plus *P_EST_‐clb1‐mDK* delays Spc72 removal and Mpc70 loading by 72 min (95% CI, 63–80; *P* < 0.0001) and 114 min (95% CI, 99–129; *P* < 0.0001; Welch's *t*‐test), respectively. (D) Time from SPB reduplication at metaphase II to Mpc70 loading plotted versus time from SPB reduplication to Spc72 removal for strains with the indicated genotypes. Diamonds represent mean values, and error bars indicate data range. Numbers of independent experiments used to calculate mean values are shown on the right.

If degradation of either Spo13 or Clb1 induces Spc72 removal, stabilization of both proteins should hinder Spc72's removal and thereby delay MP assembly and sporulation. To investigate this prediction, we first mutated the D‐box of the endogenous *SPO13* gene (Sullivan & Morgan, 2007), which results in the expression of a markedly stabilized protein, called Spo13‐mD (Fig EV2). Imaging of control and *spo13‐mD* cells revealed that Spc72 removal is delayed by 30 min and Mpc70 recruitment by 60 min in the mutant (Fig 4A and B). As a result, spore formation is reduced in *spo13‐mD* cells. At 5 h after entry into metaphase II, sporulation reaches 98% in control cells but only 33% in the *spo13‐mD* mutant. Next, we used an estradiol‐inducible promoter to express a stabilized Clb1 protein, the D‐box/KEN‐box mutant Clb1‐mDK (Okaz *et al*, 2012), at entry into metaphase I (Fig 4C). The expression of Clb1‐mDK in *spo13‐mD* cells increases the delay of Spc72 removal to 72 min and that of Mpc70 recruitment to 114 min (Fig 4A and 4C). As a result, spore formation is reduced to 22% at 5 h after entry into metaphase II. Sporulation slowly increases at later timepoints, probably because Spo13‐mD is not completely stable and Spc72 eventually disappears from SPBs (Fig EV2). By contrast, the expression of Clb1‐mDK alone has little effect on the exchange of Spc72 for Mpc70 at SPBs, and spore formation occurs with similar kinetics as in control cells (Fig 4A and C). Indeed, Spo13 is degraded normally in these cells (Fig EV2). It therefore appears that Spo13‐mD is a more potent inhibitor of Spc72 removal than Clb1‐mDK. Clb1‐mDK (bound to Cdk1) merely enhances or activates the function of Spo13‐mD, but cannot prevent Spc72 removal on its own. Next, we used the *spo13‐m2* mutation to attenuate the function of Spo13‐mD, either in the presence or in the absence of Clb1‐mDK (Fig 4B and C, right). This results in a series of strains in which an increasing delay in the removal of Spc72 correlates with a concomitant delay in the recruitment of Mpc70 (Fig 4D). Our data show that non‐degradable Spo13 delays the removal of Spc72 from SPBs beyond metaphase I, especially in the presence of non‐degradable Clb1. Interestingly, in *spo13‐mD* cells, Spc72 persists on the meiosis I‐SPBs but is not detectable at the SPBs that newly appear at metaphase II (Fig 4A). Nevertheless, Mpc70 does not occupy these SPBs until Spc72 disappears from the meiosis I‐SPBs. We speculate that Spo13 has, in fact, two functions at meiosis I: It inhibits the removal of Spc72 from SPBs and also the recruitment of MP proteins to SPBs.

### Control of spore formation by Spo13 does not require intact kinetochores

Spo13 binds to kinetochores where it is thought to coordinate the processes of sister kinetochore mono‐orientation and protection of centromeric cohesin (Katis *et al*, 2004; Lee *et al*, 2004; Kim *et al*, 2015). Thus, we asked whether Spo13's function in preventing sporulation at meiosis I depends on the integrity of kinetochores. Deletion of subunits of the inner kinetochore Ctf19‐complex, such as Mcm21 or Iml3, is compatible with proliferation but prevents kinetochore reassembly at entry into metaphase I, leading to massive chromosome miss‐segregation (Mehta *et al*, 2014; Borek *et al*, 2021). Thus, *mcm21Δ* and *iml3Δ* mutants produce four spores of low viability (3 and 18%, respectively). *P_HSL1_‐CDC20* cells lacking Mcm21 or Iml3 differ from *P_HSL1_‐CDC20 spo13Δ* cells in that they fail to undergo spore formation or nuclear division. Accordingly, the kinetochore mutants retain Spc72 at SPBs (Appendix Fig S4A and B). We conclude that Spo13 prevents premature spore formation even in the absence of intact kinetochores.

### Cdc5/PLK plays a dual role in the regulation of Spc72's removal from SPBs

Next, we analyzed the relationship between Spo13 and its binding partner, the PLK Cdc5. The *spo13‐m2* mutation in the polo‐box‐binding motif of Spo13 (Matos *et al*, 2008) causes *P_HSL1_‐CDC20 ama1Δ* cells to exchange Spc72 for Mpc70, albeit with slightly slower kinetics than the *SPO13* deletion (Fig 2A, right). The *spo13‐m2* mutation also reduces the ability of non‐degradable Spo13‐mD to delay Spc72 removal and Mpc70 recruitment when expressed alone or together with non‐degradable Clb1‐mDK (Fig 4B and C, right). We conclude that binding to Cdc5 is required for Spo13's ability to inhibit the removal of Spc72 from SPBs at metaphase I.

**Figure 5:**
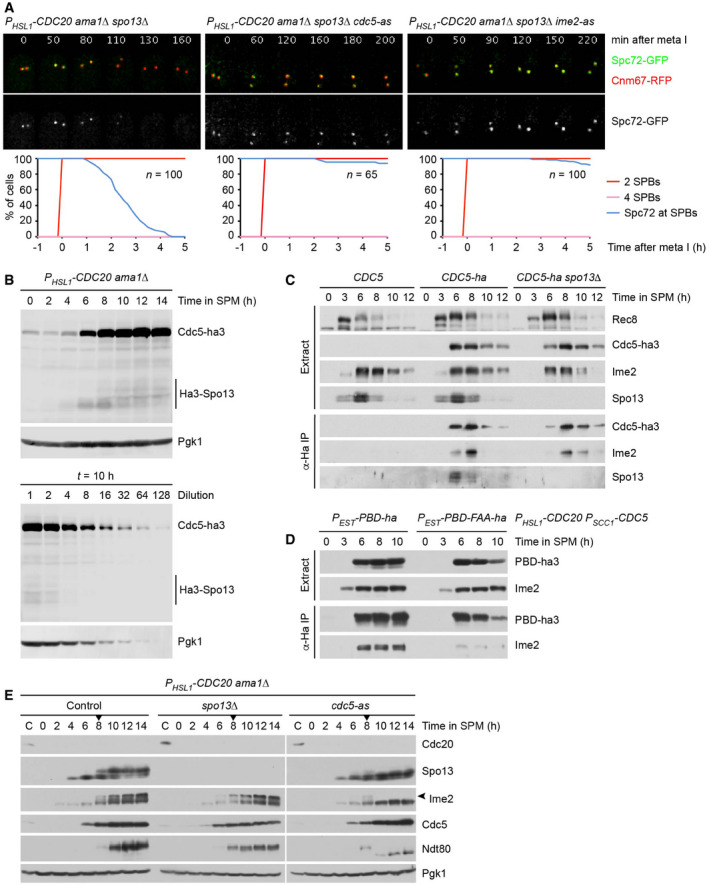
Spc72 removal from SPBs requires the activities of Cdc5 and Ime2 Imaging of SPBs (Cnm67‐RFP) and Spc72‐GFP in *P_HSL1_‐CDC20 ama1Δ spo13Δ* control cells and cells containing *cdc5‐as* or *ime2‐as* treated with CMK or 1Na‐PP1, respectively, at metaphase I (7 h in SPM). Top, time‐lapse series. Bottom, Spc72's presence at SPBs was quantified in cells synchronized *in silico* to SPB separation at entry into metaphase I (*t* = 0).Whole‐cell extracts from *P_HSL1_‐CDC20 ama1Δ CDC5‐ha3 Ha3‐SPO13* cells were subjected to α‐Ha immunoblotting. Secondary antibodies were detected by near‐infrared fluorescence imaging. Top, progression into metaphase I. Bottom, serial two‐fold dilutions of the sample collected at *t* = 10 h in SPM. The Cdc5‐ha3/Ha3‐Spo13 ratio is ~ 20.Cdc5 interacts with Ime2. Cdc5‐ha3, Ime2, and Spo13 were detected by immunoblotting in whole‐cell extracts and α‐Ha immunoprecipitates from the indicated strains.Cdc5's polo‐box domain (PBD) binds to Ime2. Ha3‐tagged versions of the wild‐type PBD or the phosphopeptide‐binding mutant PBD‐FAA were expressed at *t* = 5.5 h in SPM in cells depleted of Cdc20 and Cdc5. PBD‐ha3 and Ime2 were detected by immunoblotting in whole‐cell extracts and α‐Ha immunoprecipitates.Cdc5‐dependent modification of Ime2. *P_HSL1_‐CDC20 ama1Δ* control cells and cells containing *spo13Δ* or *cdc5‐as* were treated with CMK (to inhibit Cdc5‐as) at *t* = 8 h in SPM (arrowheads). The panel shows immunoblot analysis of whole‐cell extracts. C, sample from proliferating cells. Increased gel mobility of Ndt80 at *t* ≥ 10 h confirms inhibition of Cdc5‐as. The arrow marks the modified form of Ime2. Data information: Data in (A‐E) are representative of two independent experiments. Source data are available online for this figure.

What is the role of Cdc5 in the regulation of Spc72 removal? Cells depleted of Cdc5 arrest at metaphase I because Cdc5 activity is required for APC/C^Cdc20^‐dependent proteolysis at meiosis I (Clyne *et al*, 2003; Lee & Amon, 2003). Interestingly, these cells retain Spc72 at SPBs and differ, therefore, from *spo13Δ* cells, which remove Spc72 from SPBs even in the absence of APC/C activity (Appendix Fig S5A). Indeed, Spc72 also persists at SPBs when *spo13Δ* cells are depleted of Cdc5. Next, we used an analogue‐sensitive version of Cdc5 (Snead *et al*, 2007) to inhibit its kinase activity in *P_HSL1_‐CDC20 ama1Δ* cells that remove Spc72 due to the deletion of *SPO13* or the inhibition of Cdk1. In both cases, inhibition of Cdc5 prevents the removal of Spc72 from SPBs (Fig 5A and Appendix Fig S5B, middle). We conclude that Spc72's removal from SPBs requires Cdc5 kinase activity. This finding explains how Spc72 persists at SPBs during S‐ and prophase: On the one hand, these cells do not express Clb1, an inhibitor of Spc72 removal. On the other hand, they also lack Cdc5, an essential activator of removal.

Our data reveal that Cdc5 plays a dual role in the regulation of Spc72 removal from SPBs. While Cdc5 kinase activity promotes removal, the Cdc5‐Spo13 interaction is required for preventing removal. A parsimonious interpretation is that Spo13 prevents the Cdc5 kinase from promoting Spc72's removal. However, the levels of Spo13 at metaphase I are much lower (~ 20×) than those of Cdc5 (Fig 5B), making it unlikely that Spo13 functions as a stoichiometric Cdc5 inhibitor. We therefore hypothesize that Cdc5 exists in two different forms: The kinase activity of Cdc5 alone promotes Spc72 removal, while that of Cdc5‐Spo13 inhibits removal, even in the presence of an excess of free Cdc5.

### Spc72 removal from SPBs depends on the meiosis‐specific Ime2 kinase

Our data so far suggest that Spc72 removal requires the presence of Cdc5 and the absence of either Spo13 or Clb1. In meiotic cells, these conditions are first established at anaphase I, when Spc72 is removed from SPBs, and prevail until late anaphase II, at which time Cdc5 is degraded. However, these conditions are also met in proliferating cells at mitosis. Nevertheless, Spc72 persists at SPBs and nucleates the astral microtubules that guide the nucleus to the bud neck at metaphase (Chen *et al*, 1998; Knop & Schiebel, 1998; Soues & Adams, 1998). We hypothesized, therefore, that Spc72 removal requires an additional, meiosis‐specific activity. A promising candidate is the Ime2 kinase, which appears during early meiosis and accumulates to higher levels as cells enter metaphase I (Kominami *et al*, 1993; Benjamin *et al*, 2003). To investigate the role of Ime2, we introduced an analogue‐sensitive version (Benjamin *et al*, 2003) into *P_HSL1_‐CDC20 ama1Δ* cells, which arrest at metaphase I but remove Spc72 due to the absence of the Spo13 protein or the activity of Cdk1. In both cases, inhibition of Ime2 activity prevents the removal of Spc72 (Fig 5A and Appendix Fig S5B, right). We conclude that in addition to the activity of Cdc5 also that of Ime2 is required for Spc72's removal from SPBs at anaphase I. This requirement confines the process of Spc72 removal to meiotic cells.

What is the relationship between Cdc5 and Ime2? Immunoprecipitation experiments reveal an interaction between Cdc5 and Ime2 (Fig 5C), which is mediated by Cdc5's polo‐box domain (PBD; Fig 5D). Furthermore, Ime2 undergoes an electrophoretic mobility shift, which depends on Cdc5 activity (Fig 5E) and might reflect activation of Ime2 (Schindler & Winter, 2006). However, neither the Cdc5‐Ime2 interaction nor Ime2's mobility shift depends on Spo13. The idea that Cdc5 activates Ime2 is consistent with the finding that Cdc5 activity is required for Spc72 removal. We therefore propose that Cdc5 alone promotes Ime2's function in Spc72 removal, while Cdc5‐Spo13 inhibits it. If correct, hyperactivation of Ime2 might elicit Spc72 removal even in the presence of Spo13. To test this, we replaced *IME2* of *P_HSL1_‐CDC20 ama1Δ* cells with *IME2‐ΔC*, encoding a more stable and active version that lacks the C‐terminal domain required for rapid turnover of Ime2 (Sia & Mitchell, 1995; Sari *et al*, 2008). Ime2‐ΔC causes removal of Spc72 in metaphase I‐arrested cells (Appendix Fig S5C), albeit with slower kinetics than the *SPO13* deletion (see Figs 2A and 5A). However, Spc72 removal in *IME2‐ΔC* cells still requires the activity of Cdc5.

### Control of Spc72 removal from SPBs by Ndt80 and Cdk1

**Figure 6:**
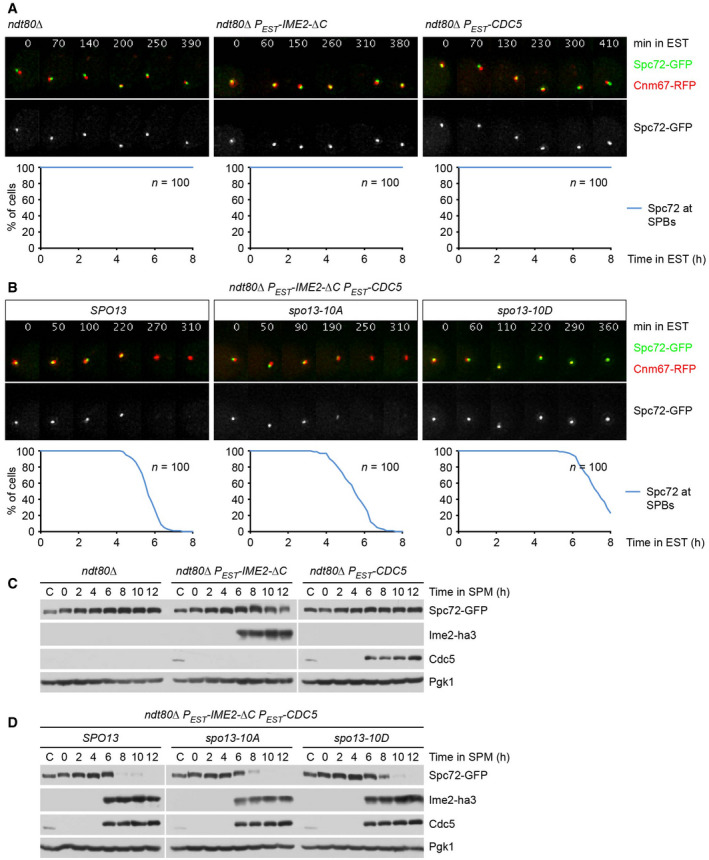
Reconstitution of the regulation of Spc72 removal from SPBs in *ndt80Δ* cells A, BImaging of SPBs (Cnm67‐RFP) and Spc72‐GFP in *ndt80Δ* cells expressing *P_EST_‐IME2‐ΔC* and/or *P_EST_‐CDC5* upon addition of estradiol (EST) at 4 h in SPM (*t* = 0). Top, time‐lapse series. Bottom, quantification of cells with Spc72 at SPBs. (A) *ndt80Δ* cells expressing Ime2‐ΔC or Cdc5. (B) Expression of Ime2‐ΔC and Cdc5 in *ndt80Δ* cells containing wild‐type Spo13 or the phospho‐site mutants Spo13‐10A and Spo13‐10D. While Spo13‐10D delays Spc72 removal by 127 min (95% CI, 105–149; *P* < 0.0001), Spo13‐10A has little effect (95% CI, −24 to 0.8; *P* = 0.07; Welch's *t*‐test).C, DImmunoblot analysis of whole‐cell extracts. C, sample from proliferating cells. (C) Protein levels in the cultures shown in (A). (D) Protein levels in the cultures shown in (B). Data information: Data are representative of two (A) or 2–4 (B) independent experiments. Source data are available online for this figure.

Cells lacking the Ndt80 transcription factor arrest at prophase and fail to accumulate or fully activate the proteins required for Spc72's removal from SPBs, namely Cdc20, Cdc5, and Ime2 (Chu & Herskowitz, 1998; Benjamin *et al*, 2003; Okaz *et al*, 2012). Of the proteins counteracting Spc72's removal, *ndt80Δ* cells contain Spo13 but lack Clb1. *ndt80Δ* cells might therefore serve as a testbed for reconstituting the regulation of Spc72's removal from SPBs. To investigate whether Ndt80‐dependent accumulation of Cdc5 and Ime2 is sufficient for removal of Spc72, we sought to express both kinases in prophase‐arrested *ndt80Δ* cells. We used Ime2‐ΔC since the wild‐type kinase is unstable at prophase and requires Ndt80 for its accumulation and activation at metaphase I (Guttmann‐Raviv *et al*, 2002; Benjamin *et al*, 2003; Berchowitz *et al*, 2013). While the expression of either Ime2‐ΔC or Cdc5 alone has no effect (Fig 6A and C), co‐expression of both kinases causes Spc72's degradation and removal from SPBs (Fig 6B and D, left). Increased levels of Ime2 and Cdc5 activity are therefore sufficient for Spc72 removal in the absence of Ndt80.

**Figure EV3:**
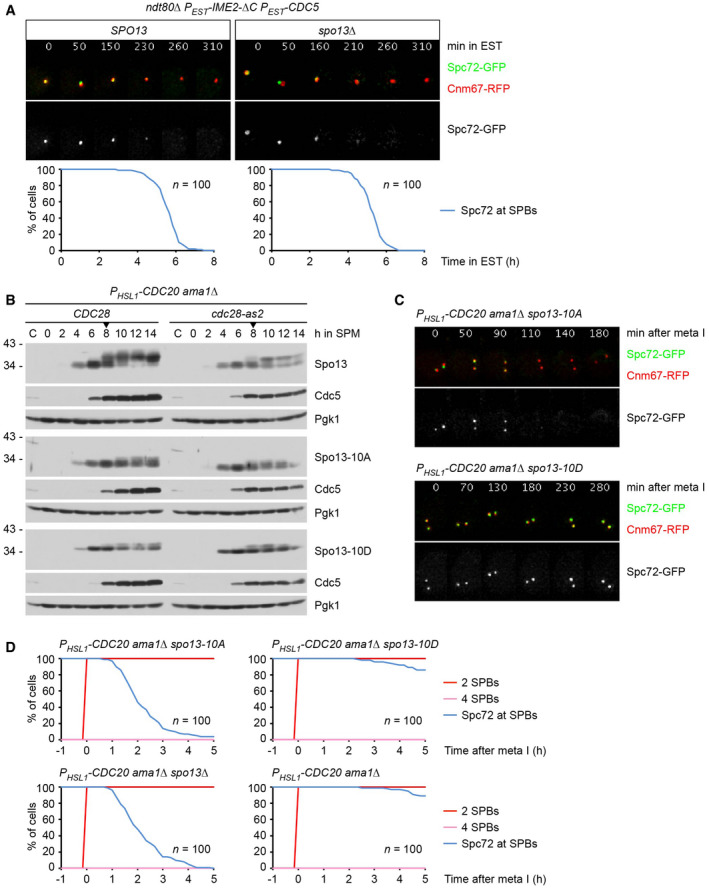
Regulation of Spo13 by Cdk1 activity AImaging of SPBs (Cnm67‐RFP) and Spc72‐GFP in *ndt80Δ* and *ndt80Δ spo13Δ* cells induced to express *P_EST_‐IME2‐ΔC* plus *P_EST_‐CDC5* at 4 h in SPM (*t* = 0). Top, time‐lapse series. Bottom, quantification of cells with Spc72 at SPBs. In *ndt80Δ* cells, the *SPO13* deletion causes only a small advance in Spc72 removal (19 min; 95% CI, 8–30; *P* = 0.001; Welch's *t*‐test).BImmunoblot detection of Spo13, Spo13‐10A, and Spo13‐10D in whole‐cell extracts from *P_HSL1_‐CDC20 ama1Δ* cells progressing into metaphase I. Cdc28‐as2 was inhibited with 1Na‐PP1 at 8 h in SPM (arrow). C, sample from proliferating cells.C, DImaging of SPBs (Cnm67‐RFP) and Spc72‐GFP in *P_HSL1_‐CDC20 ama1Δ* cells containing different *SPO13* alleles. (C) Time‐lapse series of *spo13‐10A* and *spo13‐10D* cells. (D) Spc72's presence at SPBs was quantified in *spo13‐10A*, *spo13‐10D*, *spo13Δ*, and control cells synchronized *in silico* to SPB separation at entry into metaphase I (*t* = 0). *spo13‐10D* cells remove Spc72 with similar timing after entry into metaphase I as *spo13Δ* cells (*P* = 0.64, Welch's *t*‐test). Data information: Data are representative of two (A) or three (C, D) independent experiments. Source data are available online for this figure.

In *ndt80Δ* cells expressing Ime2‐ΔC and Cdc5, the deletion of *SPO13* has only a small effect on the kinetics of Spc72 removal, suggesting that Spo13 is inactive in these cells (Fig EV3A). Spo13 might be inactive because *ndt80Δ* cells cannot produce Cdk1‐Clb1 activity. Thus, we investigated the hypothesis that Cdk1‐Clb1 phosphorylates and thereby activates Spo13. In extracts from metaphase I‐arrested cells, Spo13 shows several bands of reduced electrophoretic mobility (Fig EV3B). These slower‐migrating bands are reduced upon inhibition of Cdk1, suggesting that they result from Cdk1‐dependent phosphorylation. Spo13 contains 10 potential phosphorylation sites for Cdk1 (Ser/Thr‐Pro). Mutating these motifs to non‐phosphorylatable Ala‐Pro (*spo13‐10A*) or to phospho‐mimetic Asp‐Pro (*spo13‐10D*) also reduces the slower‐migrating species of Spo13, suggesting that at least some of these sites are phosphorylated in the wild‐type protein (Fig EV3B). In metaphase I‐arrested *P_HSL1_‐CDC20 ama1Δ* cells, the *spo13‐10A* allele causes removal of Spc72 similar to the *SPO13* deletion. By contrast, *spo13‐10D* resembles wild‐type *SPO13* in that it retains Spc72 at SPBs (Fig EV3C and D). These data support the idea that Spo13 function depends on its phosphorylation by Cdk1.

Next, we introduced the *spo13* alleles into *ndt80Δ* cells that remove Spc72 due to the expression of Ime2‐ΔC and Cdc5. While *spo13‐10A* has little effect, *spo13‐10D* delays Spc72's removal by 127 min (Fig 6B and D). Thus, mimicking Cdk1‐dependent phosphorylation restores Spo13's activity as an inhibitor of Cdc5/Ime2‐mediated removal of Spc72 in cells that lack M‐phase‐Cdk1 activity. Our data suggest that Ndt80 switches the system from a state where Spc72 removal is neither promoted nor repressed to a state where proteins required for removal coexist with proteins counteracting removal. While Spo13 and Cdk1‐Clb1 prevail at metaphase I, their degradation via APC/C^Cdc20^ at anaphase I unleashes the Cdc5/Ime2‐dependent mechanism that removes Spc72 from SPBs.

### Reconstitution of Spc72 removal and MP assembly in mitotic cells

**Figure 7:**
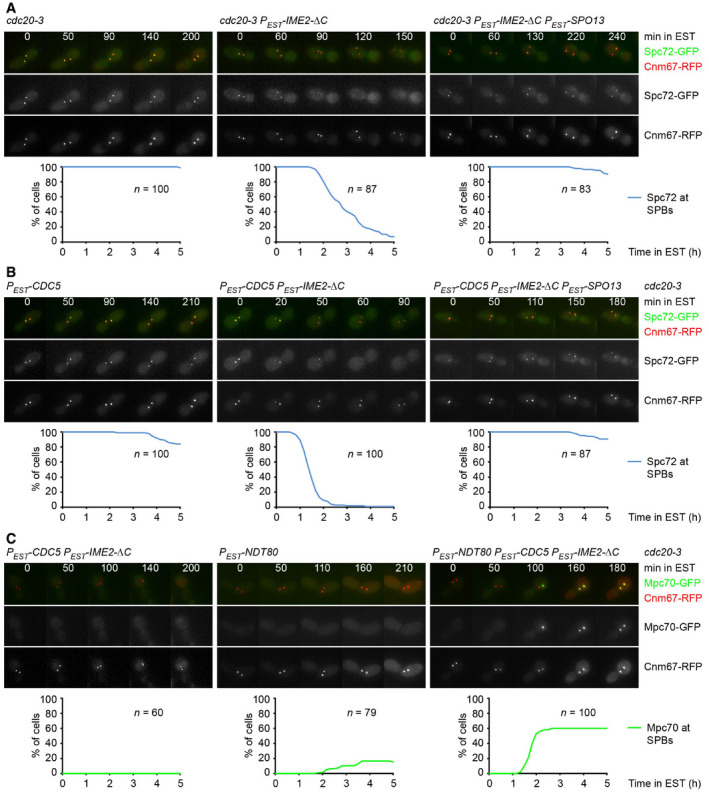
Reconstitution of Spc72 removal and MP assembly in mitotic cells A, BMitotic *cdc20‐3* cells were shifted to 36°C for 80 min. At *t* = 0, cells were treated with estradiol to induce expression from the *P_EST_* promoter. Top, time‐lapse series from the imaging of SPBs (Cnm67‐RFP) and Spc72‐GFP. Frame width, 19 μm. Bottom, quantification of cells with Spc72‐GFP at SPBs. (A) Expression of Ime2‐ΔC induces removal of Spc72 from SPBs, which is inhibited by co‐expression of Spo13. (B) Expression of Cdc5 plus Ime2‐ΔC causes rapid removal of Spc72, which is inhibited by co‐expression of Spo13.CMitotic *cdc20‐3* cells containing *P_EST_‐CDC5* plus *P_EST_‐IME2‐ΔC* and/or *P_EST_‐NDT80* were shifted to 36°C for 30 min and subsequently treated with estradiol (*t* = 0). Top, time‐lapse series from the imaging of SPBs (Cnm67‐RFP) and Mpc70‐GFP. Frame width, 19 μm. Bottom, quantification of cells with Mpc70‐GFP at SPBs. Data information: Data are representative of three (A, B) or two (C) independent experiments.

**Figure EV4:**
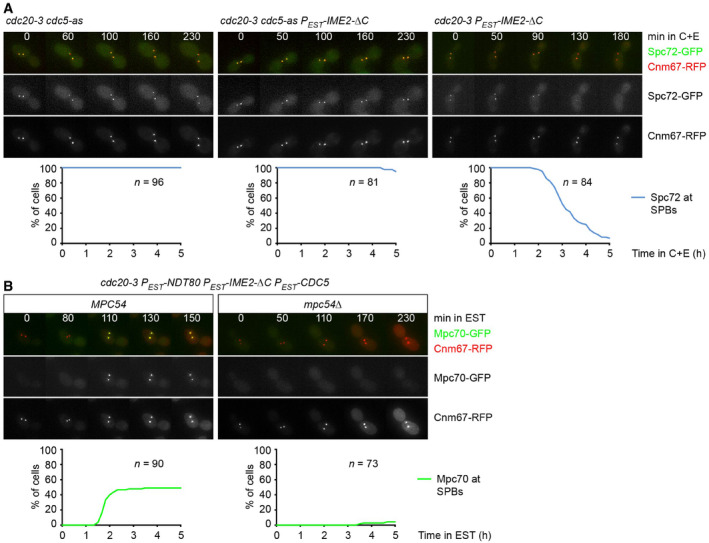
Analysis of Spc72 removal and MP assembly in mitotic cells Mitotic *cdc20‐3* cells containing *cdc5‐as* and/or *P_EST_‐IME2‐ΔC* were shifted to 36°C for 80 min. At *t* = 0, cells were treated with CMK and estradiol (C + E) to inhibit Cdc5‐as and induce *P_EST_‐IME2‐ΔC*, respectively. Top, time‐lapse series from the imaging of SPBs (Cnm67‐RFP) and Spc72‐GFP. Frame width, 19 μm. Bottom, quantification of cells with Spc72‐GFP at SPBs.Mitotic *cdc20‐3 MPC54* and *cdc20‐3 mpc54Δ* cells containing *P_EST_‐NDT80*, *P_EST_‐IME2‐ΔC*, and *P_EST_‐CDC5* were shifted to 36°C for 30 min and treated with estradiol (*t* = 0). Top, time‐lapse series from the imaging of SPBs (Cnm67‐RFP) and Mpc70‐GFP. Frame width, 19 μm. Bottom, quantification of cells with Mpc70‐GFP at SPBs. Data information: Data are representative of two (A) or three (B) independent experiments.

Are Ime2 and Spo13 sufficient for the meiosis‐specific control of Spc72's removal from SPBs? To address this question, we used the ts‐mutation *cdc20‐3* to arrest mitotic cells at metaphase (Shirayama *et al*, 1998). As shown in Fig 7A, the expression of Ime2‐ΔC results in Spc72's removal from SPBs. Spc72 removal requires the endogenous Cdc5 activity present at mitosis (Fig EV4A) and is accelerated by overexpression of Cdc5 (Fig 7B, middle). By contrast, overexpression of Cdc5 alone has little effect (Fig 7B, left). Remarkably, Spc72 removal elicited by Ime2‐ΔC or Ime2‐ΔC plus Cdc5 is inhibited by the co‐expression of Spo13 (Fig 7A and B, right). Thus, the expression of Ime2 and Spo13 is sufficient to reconstitute key aspects of the meiosis‐specific regulation of Spc72 removal from SPBs in mitotic cells. Next, we asked whether mitotic SPBs are capable of MP assembly if Spc72 is removed. Thus, we expressed Ndt80 in metaphase‐arrested cells to enable the synthesis of MP components, including GFP‐tagged Mpc70 (Fig 7C). In a small fraction of these cells (16%), SPBs become weakly labeled with Mpc70, probably because Ndt80 also promotes the expression of endogenous Ime2 and Cdc5. By contrast, co‐expression of Ndt80, Ime2‐ΔC, and Cdc5 causes robust accumulation of Mpc70 at SPBs in 60% of cells (Fig 7C). Mpc70's recruitment to SPBs is abrogated upon deletion of *MPC54* (Fig EV4B), which is consistent with the finding that MP assembly requires the expression of all three MP components (Knop & Strasser, 2000; Bajgier *et al*, 2001; Nickas *et al*, 2003). Taken together, our data suggest that Spc72 removal requires the activities of Ime2 and Cdc5 and the absence of Spo13. Once Spc72 has been removed, MP proteins expressed by Ndt80 require the environment of metaphase to form MPs. These conditions would limit MP assembly to metaphase II of meiosis but do not exist in a normal mitosis.

### MP assembly is promoted by Cdk1‐Clb and inhibited by Cdc5‐Spo13

**Figure EV5:**
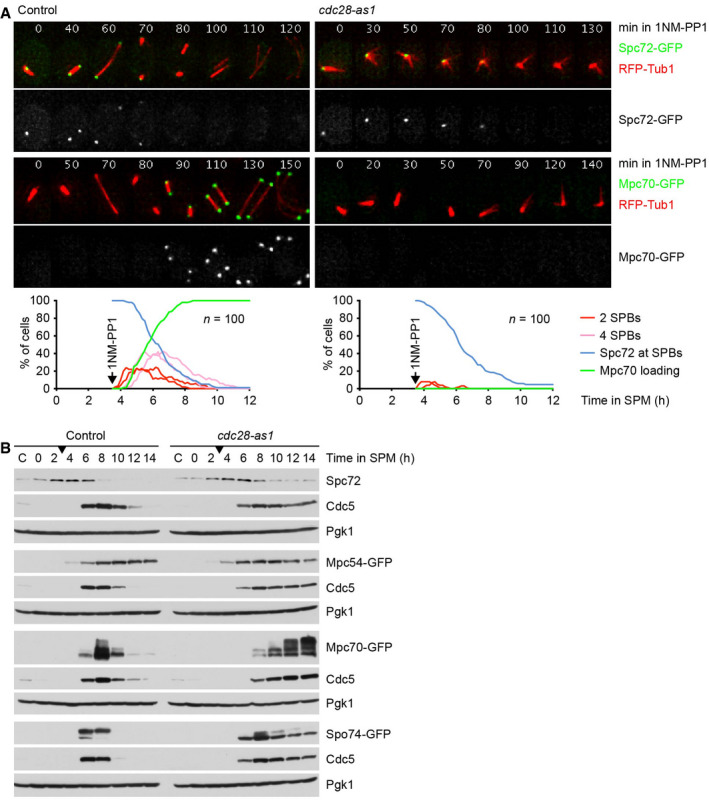
Analysis of Spc72 and MP proteins after inhibition of Cdk1 Imaging of microtubules (RFP‐Tub1) and Spc72‐GFP or Mpc70‐GFP in control and *cdc28‐as1* cells treated with 1NM‐PP1 at 3.5 h in SPM. Top, time‐lapse series. Bottom, quantification of meiotic events. Graphs show overlays of *SPC72‐GFP* and *MPC70‐GFP* strains.Immunoblot detection of Spc72 and GFP‐tagged MP proteins in whole‐cell extracts from control and *cdc28‐as1* strains treated with 1NM‐PP1 at 3 h in SPM (arrow). The appearance of Cdc5 marks the activation of Ndt80. C, sample from proliferating cells. Source data are available online for this figure.

**Figure 8:**
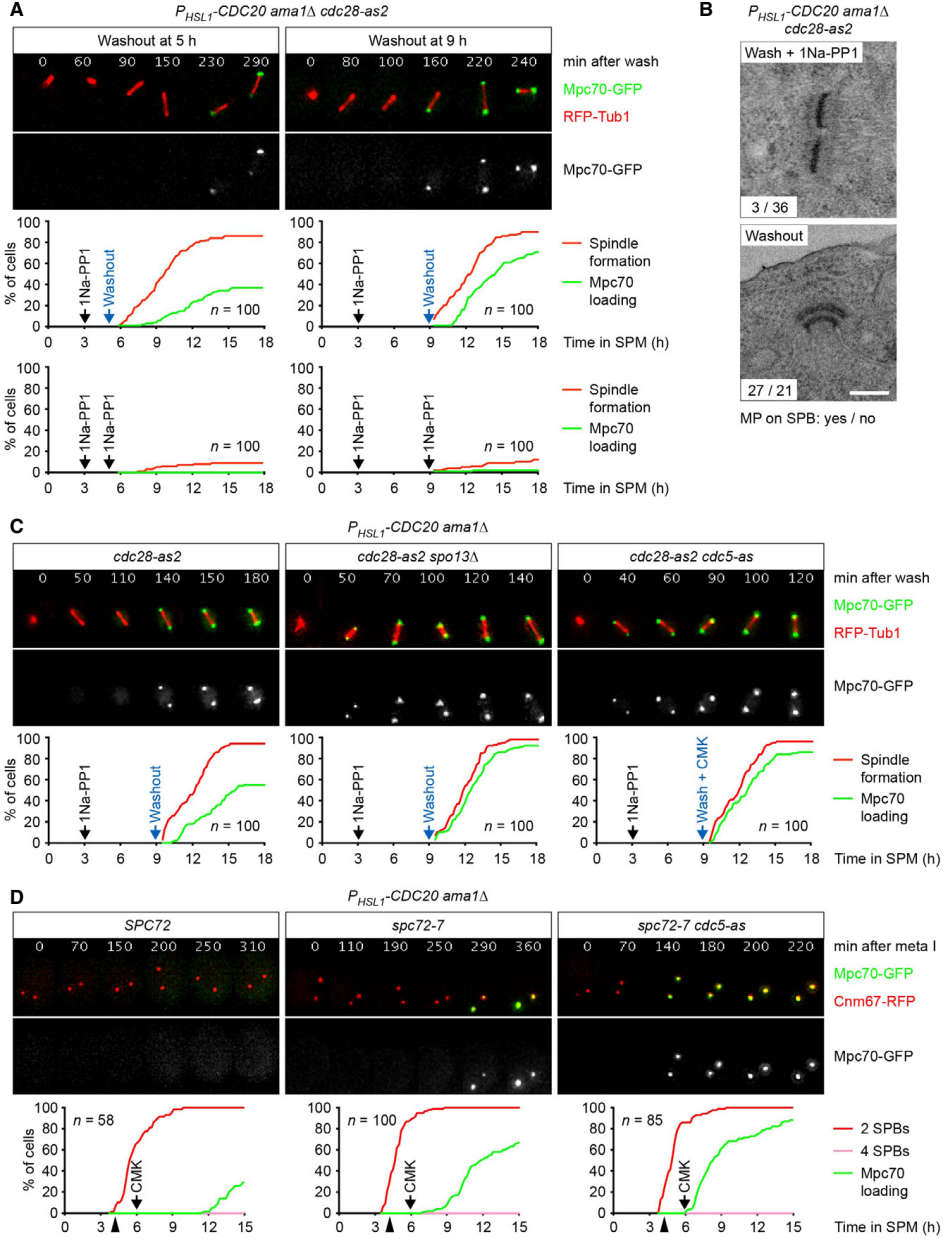
MP assembly is promoted by Cdk1‐Clb and inhibited by Cdc5‐Spo13 *P_HSL1_‐CDC20 ama1Δ cdc28‐as2* cells were treated with 1Na‐PP1 at *t* = 3 h in SPM to inhibit Cdc28‐as2. At *t* = 5 h (left) or *t* = 9 h (right), Cdc28‐as2 was activated by washing cells with conditioned SPM (cSPM) lacking 1Na‐PP1 (washout). Top, time‐lapse series from the imaging of Mpc70‐GFP and RFP‐tubulin. The weak, nuclear signal from Ndt80‐GFP serves to confirm entry into meiosis. Middle, quantification of spindle formation and Mpc70 loading to SPBs. Bottom, quantification of control experiments. Cells were washed with cSPM plus 1Na‐PP1 at *t* = 5 h (left) or *t* = 9 h (right) in SPM.TEM analysis of SPBs. *P_HSL1_‐CDC20 ama1Δ cdc28‐as2* cells were treated with 1Na‐PP1 at 3 h in SPM. At 9 h in SPM, cells were washed with cSPM containing (top) or lacking 1Na‐PP1 (washout, bottom). TEM samples were collected at 13 h in SPM. Washout of 1Na‐PP1 causes MP formation (*P* < 0.0001, Fisher's exact test). Scale bar, 0.2 μm.
*P_HSL1_‐CDC20 ama1Δ cdc28‐as2* control cells and cells containing *spo13Δ* or *cdc5‐as* were treated with 1Na‐PP1 at *t* = 3 h in SPM to inhibit Cdc28‐as2. At *t* = 9 h in SPM, Cdc28‐as2 was activated by washing cells with cSPM lacking 1Na‐PP1 (washout). *cdc5‐as* cells were washed with cSPM plus CMK to activate Cdc28‐as2 and inhibit Cdc5‐as. Top, time‐lapse series from the imaging of Mpc70‐GFP and RFP‐tubulin. The weak, nuclear signal originates from Ndt80‐GFP. Bottom, quantification of spindle formation and Mpc70 loading to SPBs.
*P_HSL1_‐CDC20 ama1Δ* cells containing *SPC72*, *spc72‐7*, or *spc72‐7* plus *cdc5‐as* were shifted from 24 to 36°C at *t* = 4.2 h in SPM (to inactivate Spc72‐7, arrowheads) and treated with CMK at *t* = 6 h in SPM (to inhibit Cdc5‐as). Top, time‐lapse series from the imaging of SPBs (Cnm67‐RFP) and Mpc70‐GFP. Bottom, quantification of SPB separation and Mpc70 loading to SPBs. Inhibition of Cdc5‐as in *spc72‐7* cells advances the time of Mpc70 loading by 119 min (95% CI, 76–162; *P* < 0.0001; Welch's *t*‐test). Data are representative of two independent experiments.

Next, we investigated the role of Cdk1‐Clb kinases in MP assembly. While inhibition of Cdk1 in metaphase I‐arrested cells causes removal of Spc72 due to the inactivation of Spo13, it does not elicit the recruitment of Mpc70 to SPBs (Fig 2C). Likewise, Spc72 is removed from SPBs and degraded, but Mpc70 fails to appear at SPBs, when cells enter meiosis without being able to activate Cdk1 (Fig EV5A and B, top). However, MP proteins accumulate normally in the absence of Cdk1 activity (Fig EV5B), implying that MP assembly requires the activity of Cdk1 as well as the removal of Spc72. To test this, we sought to first prevent the activation of Cdk1 for a period of time that allows for the synthesis of MP proteins and the removal of Spc72. We would then activate Cdk1 and ask whether this results in MP assembly. We used *cdc28‐as2* cells, whose Cdk1 can be inhibited with the ATP‐analogue 1Na‐PP1 and subsequently activated by washout of the inhibitor. These cells lack Cdc20 and Ama1 to prevent progression beyond metaphase I. As shown in Fig 8A, the addition of 1Na‐PP1 at prophase followed by its washout either two or six h later induces spindle formation with similar kinetics. However, Mpc70 is recruited to SPBs more efficiently in the latter (≥ 70% of cells) than in the former case (37% of cells). TEM confirmed that late activation of Cdk1 causes the formation of normal‐looking MPs (Fig 8B). These data suggest that Cdk1 activation induces MP assembly provided that Spc72 has been removed from SPBs due to the absence of Cdk1 and/or Spo13 activity.

Since Spo13 is not degraded in *P_HSL1_‐CDC20 ama1Δ* cells, it should regain its function upon reactivation of Cdk1. Thus, the washout experiment allows us to investigate whether Spo13 affects MP assembly after Spc72 has been removed from SPBs. Indeed, Mpc70 recruitment in response to Cdk1 reactivation is more efficient in *spo13Δ* cells (Fig 8C, middle). A similar effect is observed when analogue‐sensitive Cdc5 is inhibited at the time of Cdk1 reactivation (Fig 8C, right). These data support the idea that at metaphase I, the Cdc5‐Spo13 kinase not only inhibits the removal of Spc72 but also hinders the assembly of MP proteins at SPBs. This notion provides a rationale for the slow Mpc70 recruitment in *P_HSL1_‐CDC20 ama1Δ spc72‐7* cells shifted to 36°C (Fig 3A), namely that Cdc5‐Spo13 delays MP formation even when Spc72 has been inactivated. Accordingly, inhibition of Cdc5 elicits prompt Mpc70 recruitment in these *spc72‐7* cells (Fig 8D). We conclude that Cdk1‐Clb activity promotes MP assembly, whereas Cdc5‐Spo13 activity inhibits it.

### Regulation of MP assembly and PSM formation at meiosis II

**Figure 9:**
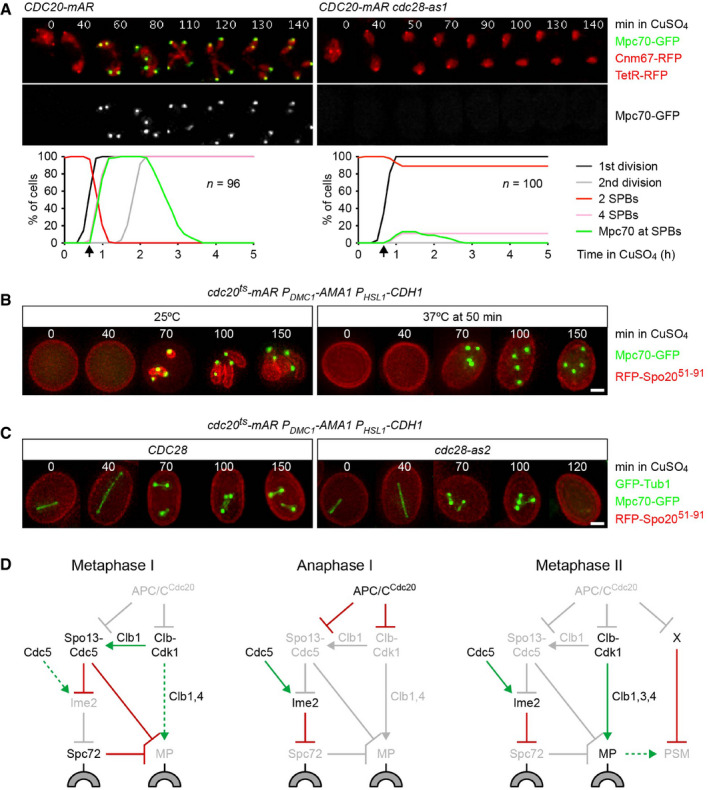
Regulation of MP assembly and PSM formation at meiosis II Inhibition of Cdk1 at anaphase I. *CDC20‐mAR* and *CDC20‐mAR cdc28‐as1* cells were released from the metaphase I‐arrest with CuSO_4_ at 7 h in SPM (*t* = 0) and treated with 1NM‐PP1 at *t* = 40 min to inhibit Cdc28‐as1 (arrows). Top, time‐lapse series from the imaging of Mpc70‐GFP, SPBs (Cnm67‐RFP), and nuclei (TetR‐RFP). Bottom, quantification of meiotic events.
*cdc20^ts^‐mAR P_DMC1_‐AMA1 P_HSL1_‐CDH1* cells were released from the metaphase I‐arrest with CuSO_4_ (*t* = 0) at 25°C. Subsequent incubation at 25°C allows progression through meiosis II (left), while a shift to 37°C at *t* = 50 min causes arrest at metaphase II (right). Mpc70‐GFP and the PSM marker RFP‐Spo20^51–91^ were imaged in cells fixed at the indicated times. RFP‐Spo20^51–91^ is expressed from an episomal plasmid (Diamond *et al*, 2009). Images represent ≥ 90% of cells harboring the plasmid. Scale bar, 2 μm.
*cdc20^ts^‐mAR P_DMC1_‐AMA1 P_HSL1_‐CDH1* cells containing *CDC28* or *cdc28‐as2* were released from metaphase I at *t* = 0 and shifted to 37°C at *t* = 50 min to cause arrest at metaphase II. 1Na‐PP1 was added at *t* = 100 min to inhibit Cdc28‐as2. Microtubules (GFP‐Tub1), Mpc70‐GFP, and RFP‐Spo20^51–91^ were imaged in cells fixed at the indicated times. Images represent ≥ 90% of cells carrying the RFP‐Spo20^51–91^ plasmid. Scale bar, 2 μm.The regulatory network controlling MP assembly at different stages of meiosis. Green arrows: activation. Dashed, green arrows: latent activation overwhelmed by inhibition. Red, bar‐headed lines: inhibition. Items in light gray are inactive or absent. Block arc: SPB outer layer. X: Cdc20 substrate that blocks PSM formation at metaphase II. See text for details. Data information: Data in (B) and (C) are representative of two independent experiments.

In wild‐type cells, APC/C^Cdc20^ inactivates Cdc5‐Spo13 and Cdk1‐Clb at anaphase I. On the one hand, this unblocks SPBs and enables MP assembly. On the other hand, APC/C^Cdc20^ destroys the Cdk1 activity required for MP assembly. It follows that MP assembly has to await the reactivation of Cdk1 at entry into metaphase II. To confirm this, we sought to inhibit Cdk1 at anaphase I. Thus, we used *CDC20‐mAR* cells, which initially arrest at metaphase I due to depletion of endogenous Cdc20. Cells are then released into anaphase I by a copper‐inducible *CDC20* gene (Arguello‐Miranda *et al*, 2017). Treatment of *CDC20‐mAR cdc28‐as1* cells with 1NM‐PP1 at anaphase I blocks not only spindle formation and nuclear division at meiosis II but also Mpc70 recruitment to SPBs (Fig 9A). While inhibition of analogue‐sensitive Cdc5 or Ime2 at anaphase I also blocks the meiosis II division, it does not hinder the recruitment of Mpc70 to SPBs (Appendix Fig S6). Thus, Cdc5 and Ime2 promote MP formation only indirectly, by mediating Spc72's removal from SPBs at anaphase I. We conclude that MP assembly at metaphase II depends on the reactivation of Cdk1 after its inactivation at anaphase I. Which Cdk1‐Clb kinase promotes MP assembly? Inhibition of analogue‐sensitive Cdc28/Cdk1 and deletion of *CLB1* cause removal of Spc72. While the former blocks subsequent MP assembly, the latter merely delays it (Fig 2C). This implies that Clb1 promotes MP assembly but is assisted and can be replaced in this function by Clb3 and/or Clb4.

One might expect that PSM formation at meiosis II is coordinated with nuclear division and spindle elongation, both of which depend on APC/C^Cdc20^. To investigate PSM formation, we used *cdc20^ts^‐mAR* *ama1 cdh1* cells, which initially arrest at metaphase I due to the depletion of all three APC/C activators. Cells are released into anaphase I by copper‐inducible expression of the ts‐protein Cdc20‐3 (Mengoli *et al*, 2021). At 25°C, cells progress through meiosis II, while a shift to 37°C causes arrest at metaphase II. Cells recruit Mpc70 to SPBs at both 25 and 37°C (Fig 9B), which is consistent with the notion that APC/C activity is dispensable for MP formation once Spc72 has been removed from SPBs. While PSMs emerge at 25°C, they are misshapen due to the absence of Ama1 (Diamond *et al*, 2009). At 37°C, however, cells fail to generate PSMs, suggesting that APC/C^Cdc20^ activity is required for PSM formation. Next, we used the *cdc28‐as2* mutation to inhibit Cdk1 in cells arrested at metaphase II (Fig 9C). MPs as well as spindles disappear upon inhibition of Cdk1, suggesting that Cdk1 activity is required for both assembly and persistence of MPs at metaphase II. However, Cdk1 inhibition does not elicit PSM formation, implying that this process involves degradation of Cdc20 substrates other than cyclins.

In summary, our data suggest that MP formation requires three steps (Fig 9D). First, Ndt80 produces MP proteins and regulators that promote or inhibit the removal of Spc72 from SPBs. However, Cdc5‐Spo13, the inhibitor of Spc72 removal and MP assembly, prevails at metaphase I. Second, APC/C^Cdc20^‐dependent inactivation of Cdc5‐Spo13 at anaphase I unblocks SPBs and licenses MP proteins for assembly. Third, the reappearance of Cdk1 activity at metaphase II induces MP assembly. Subsequent activation of APC/C^Cdc20^ initiates PSM formation, which leads to spore wall synthesis. Since activation of APC/C^Cdc20^ depends on silencing of the SAC, the SAC ultimately controls Spc72 removal at meiosis I and PSM formation at meiosis II. Thus, the SAC coordinates spore formation as well as nuclear division with the biorientation of chromosomes.

## Discussion

### Coordination of meiotic divisions and gamete differentiation

Sporulation in yeast and spermatogenesis in animals have in common that gamete differentiation is initiated at or after the second division of meiosis. Furthermore, mRNAs for cell cycle regulators and differentiation factors are produced by the same transcriptional program. One might therefore expect that the initiation of differentiation is somehow coordinated with progression through the two divisions of meiosis to ensure the formation of euploid gametes. However, work in *Drosophila* implies that spermiogenesis proceeds independently of cell cycle controls (White‐Cooper *et al*, 1998). For instance, spermiogenesis occurs even when Cdk1 cannot be activated (Sigrist *et al*, 1995). Our analysis of the control of spore differentiation in yeast paints a different picture. Spore formation requires that the cytoplasmic face of the SPB/centrosome switches from microtubule nucleation by the γ‐TuC receptor Spc72 to membrane nucleation by the MP. While MP proteins appear at metaphase I, they can only assemble into MPs at metaphase II and initiate PSM formation at anaphase II. We show that APC/C^Cdc20^ mediates Spc72 removal and MP assembly through a regulatory network composed of Spo13/Meikin, the PLK Cdc5, the CDK Cdk1‐Clb1, and Ime2, the yeast homologue of male germ cell‐associated kinase (MAK; Fu *et al*, 2005). Thus, yeast employs conserved cell cycle regulators to coordinate spore differentiation with nuclear division. APC/C^Cdc20^ connects this network to the SAC, which inhibits APC/C^Cdc20^ until kinetochores come under tension from bipolar spindle forces. SAC activity delays spore formation by preventing APC/C^Cdc20^ from inducing Spc72 removal at meiosis I and PSM formation at meiosis II. This ensures the formation of euploid spores despite the variability in the duration of meiosis that arises from the process of bi‐orienting chromosomes at each division.

Is there evidence for regulation of gamete differentiation by the cell cycle machinery also in animals? Inactivation of Cdk1 causes mouse spermatocytes to arrest at late prophase for prolonged periods of time without inducing apoptosis (Clement *et al*, 2015). In contrast to *Drosophila*, post‐meiotic events or aspects of differentiation do not occur, indicating that spermiogenesis requires processes set into motion by Cdk1 activity at meiosis. Links between meiosis and differentiation might also become apparent when APC/C^Cdc20^ is inhibited by the SAC. In mouse spermatocytes unable to form chiasmata, SAC activity induces apoptosis soon after entry into metaphase I, thereby preventing the production of aneuploid sperm (Eaker *et al*, 2002; Faisal & Kauppi, 2017). By contrast, in zebrafish and even more so in some insects (e.g., grasshopper or praying mantid), the SAC is capable of prolonging metaphase I of male meiosis for considerable amounts of time before inducing apoptosis (Dooley, 1941; Li & Nicklas, 1995; Nicklas *et al*, 1995; Poss *et al*, 2004; Leal *et al*, 2008). In these cases, the SAC reduces the incidence of aneuploidy by providing additional time for error correction. This implies that a delay during meiosis can be transmitted to the differentiation program, and APC/C^Cdc20^ would be expected to control differentiation as well as nuclear division. *Drosophila* and yeast might represent the extremes of a spectrum between no control and strict control of gamete differentiation by the meiotic cell cycle machinery.

### A three‐step sequence confines MP assembly to metaphase II

While meiosis I is preceded by high transcriptional activity in yeast and animals, there is no evidence for a transcriptional program specifically dedicated to meiosis II (Monesi, 1964; Olivieri & Olivieri, 1965; Chu *et al*, 1998; Brar *et al*, 2012). In yeast, the Ndt80 transcription factor is active from the onset of metaphase I until anaphase II (Chu & Herskowitz, 1998), which ensures the synthesis of cell cycle regulators at both divisions. However, Ndt80 also produces proteins that control meiosis II‐specific processes, including spore differentiation. While posttranslational modifications might activate these proteins at meiosis II, the underlying mechanisms have remained unclear. Yeast employs translational control to restrict the accumulation of certain proteins, such as the cyclin Clb3, to meiosis II (Carlile & Amon, 2008; Berchowitz *et al*, 2013). However, the temporal control of meiosis II‐specific translation is unclear, and the number of these proteins is relatively small (Brar *et al*, 2012; Jin *et al*, 2015). In male animals, by contrast, transcription is downregulated soon after entry into meiosis I, and translational control is the major mechanism that determines when proteins become available (Schafer *et al*, 1995). However, how translation is coordinated with the progression of meiosis and differentiation is not understood. Our work provides an example of how proteins that accumulate at metaphase I are activated at metaphase II by the regulated access to a subcellular structure, namely the SPB/centrosome.

During meiotic S‐ and prophase, Spc72 is bound to SPBs, while MP proteins are absent. Also absent are the proteins that either promote or inhibit Spc72's removal from SPBs. This phase might be regarded as the off‐state of the regulatory system. Upon completion of recombination, Ndt80 produces MP components and proteins required for Spc72's removal (i.e., Cdc20, Cdc5, Ime2). However, Ndt80 also produces Clb1, which counteracts removal by generating the Cdk1‐Clb1 activity that phosphorylates and thereby activates Spo13. As a result, Cdc5‐Spo13 prevents Ime2 from eliciting the removal of Spc72 from SPBs. Cdc5‐Spo13 inhibits Spc72 removal even though free Cdc5 is an activator of Ime2's function in Spc72 removal. In addition, Cdc5‐Spo13 prevents MP proteins from assembling into MPs. Thus, all components of the regulatory network are present at metaphase I, while the inhibitors of Spc72 removal and MP assembly have the upper hand (Fig 9D, left). However, the persistence of Spc72 at SPBs now relies on the stability of Spo13 and Clb1, which is controlled by APC/C^Cdc20^. Upon silencing of the SAC, APC/C^Cdc20^ mediates the degradation Spo13 and Clb1, which eliminates the activity of Cdc5‐Spo13. Cdc5 now becomes an activator of Ime2, which elicits, in turn, Spc72's removal from SPBs. Furthermore, the inactivation of Cdc5‐Spo13 licenses MP proteins for assembly. Accordingly, SPBs are ready for MP assembly at anaphase I (Fig 9D, middle). However, they cannot recruit MP proteins yet because the APC/C activity required for Spc72 removal also destroys the Cdk1 activity essential for MP assembly. Thus, it is the second wave of Cdk1 activity that induces MP assembly at metaphase II (Fig 9D, right). Silencing of the SAC and subsequent activation of APC/C^Cdc20^ initiates formation of the PSM, the precursor of the spore wall.

Unlike wild‐type cells, *cdc20 spo13Δ* and *cdc20 clb1Δ* mutants sporulate while undergoing only a single division (Katis *et al*, 2004; Fig 1A and 1B). We propose that this phenotype arises from two effects: First, the absence of Cdc20 creates a prolonged metaphase during which conditions allowing Spc72 removal (Spo13 absent or inactive) coexist with conditions promoting MP assembly (Cdk1 activity elevated). Second, when Spo13 is inactive, Ama1 can assume functions that normally depend on Cdc20, namely nuclear division and PSM formation. Similar conditions might prevail in the *spo13Δ* single mutant, in which APC/C^Cdc20^ is inhibited by the SAC (Shonn *et al*, 2002).

### Dynamics of the regulatory network

Cdk1 and APC/C^Cdc20^ are the key components of an oscillatory system that generates waves of Cdk1 and APC/C^Cdc20^ activity, which are, in essence, identical to each other (Sha *et al*, 2003; Pomerening *et al*, 2005). This raises the question of how cells skip the first M‐phase and assign MP assembly to the second one. Three aspects are worth considering: the starting conditions at entry into meiosis, the “wiring” of the components of the regulatory network, and the properties of Spo13. In principle, MP proteins can assemble into MPs at the high‐Cdk1 state of metaphase I (Figs 2 and 3). However, Spc72 already occupies SPBs when cells enter meiosis, while MP proteins are only synthesized upon activation of Ndt80 (Knop & Strasser, 2000; Gordon *et al*, 2006). Thus, it is history rather than biochemistry that gives Spc72 the upper hand over MP assembly at metaphase I. This renders MP assembly dependent on APC/C^Cdc20^‐mediated degradation of Spo13, which unblocks SPBs through the removal of Spc72. However, the wiring between Ndt80, Cdk1‐Clb, and APC/C^Cdc20^ dictates that the Cdk1 activity produced by Ndt80 eventually activates APC/C^Cdc20^ (Okaz *et al*, 2012). As a result, Cdk1 is inactivated at anaphase I, and MP assembly is only possible at the second wave of Cdk1 activity, that is, at metaphase II (Fig 9A). Cdc5‐Spo13 also prevents MP assembly directly, by inhibiting the ability of MP proteins to accumulate at SPBs (Fig 8C). Consequently, efficient MP assembly requires the APC/C^Cdc20^‐dependent degradation of Spo13 and/or Clb1, which helps to confine MP assembly to the second M‐phase.

An important feature of Spo13 and its orthologue in fission yeast (Moa1) is their presence only at meiosis I (Katis *et al*, 2004; Lee *et al*, 2004; Yokobayashi & Watanabe, 2005; Kim *et al*, 2015). While Spo13 is degraded in an APC/C^Cdc20^‐dependent manner at anaphase I (Sullivan & Morgan, 2007), it fails to reaccumulate at metaphase II when APC/C^Cdc20^ is inactive. We propose that Spo13 cannot reappear because its transcription is downregulated after meiosis I (Wang *et al*, 1987; Brar *et al*, 2012), which makes Spo13 inactivation an irreversible process. This creates two, mutually exclusive states: At metaphase I, Spo13 is stable and Cdc5‐Spo13 is active. Spc72 therefore persists at SPBs, and the assembly of MP proteins is inhibited. At meiosis II, when Spo13 is absent, Spc72 is removed from SPBs and subject to proteolysis. Furthermore, *SPC72* transcription ceases at entry into meiosis I (Brar *et al*, 2012), thereby preventing the re‐accumulation of Spc72. SPBs are therefore free to receive MP proteins, which are now licensed for assembly. As a result, Cdk1 is activated under different conditions at meiosis I and meiosis II. At metaphase I, Cdk1 is activated while Spc72 is bound to SPBs. In fact, by activating Spo13, Cdk1 promotes Spc72's persistence at SPBs and inhibits the ability of MP proteins to assemble (Fig 6). At metaphase II, Cdk1 activity rises in the absence of Spo13 and Spc72 and therefore triggers MP assembly. Together, the starting conditions at entry into meiosis, the wiring of the network, and the properties of Spo13 create differences between the two M‐phases, which are ultimately responsible for the different outcomes of meiosis I and meiosis II.

The principles outlined here might serve as a paradigm for the regulation of other meiosis II‐specific processes. APC/C^Cdc20^‐dependent proteolysis of inhibitory proteins serves to unleash events at entry into anaphase I. For instance, degradation of Pds1, the inhibitor of separase, triggers cohesin cleavage at the onset of anaphase I (Buonomo *et al*, 2000). By contrast, meiosis II‐specific processes have to be delayed at least until entry into metaphase II. This might be achieved by a two‐step mechanism: First, APC/C^Cdc20^‐dependent degradation of Spo13 at anaphase I leads to the inactivation of an inhibitor of the meiosis II‐specific process. Since Spo13 does not reappear after anaphase I, the inhibitor remains inactive during meiosis II. Second, the meiosis II‐specific process is triggered by the appearance of an essential activator, such as Cdk1‐Clb, which is linked to the onset of M‐phase, but is not necessarily specific to meiosis II. An additional requirement for the activity of APC/C^Cdc20^ would allow the control of events specific to anaphase II, such as PSM formation (Fig 9B) or the deprotection of centromeric cohesin (Mengoli *et al*, 2021).

While the yeast Cdc5‐Spo13 kinase is only active at metaphase I, its mammalian counterpart might function at both metaphase I and metaphase II. Plk1 bound to full‐length Meikin is present at metaphase I and might inhibit meiosis II‐specific processes similar to Cdc5‐Spo13 in yeast. However, cleavage of Meikin by separase at anaphase I releases a C‐terminal fragment that binds to Plk1 until being degraded by APC/C‐dependent proteolysis at anaphase II (Maier *et al*, 2021). This second form of the Plk1‐Meikin kinase might then promote meiosis II‐specific processes.

### Regulation of Spc72 removal by Cdc5 and Ime2

While we describe a regulatory network capable of confining MP assembly to meiosis II, many of its biochemical mechanisms remain to be elucidated. It is unclear, for instance, how predominantly nuclear proteins control events at the cytoplasmic face of the SPB. Spo13 binds to kinetochores but prevents premature spore formation also in cells unable to assemble kinetochores (Appendix Fig S4). Indeed, Spo13 is detectable throughout the nucleus (Katis *et al*, 2004; Sullivan & Morgan, 2007). It is unclear, however, whether Spo13 needs to be exported from the nucleus to inhibit Spc72 removal and MP assembly at SPBs. While Cdc5 and Ime2 accumulate in the nucleus (Kominami *et al*, 1993; Matos *et al*, 2008), their substrates include cytoplasmic as well as nuclear proteins (Song *et al*, 2000; Holt *et al*, 2007; Snead *et al*, 2007; Berchowitz *et al*, 2013). Cdc5 has been shown to bind to components of the SPB outer layer, such as Spc72 and Cnm67, but cannot elicit Spc72 removal on its own (Song *et al*, 2000; Park *et al*, 2004; Snead *et al*, 2007). However, Cdc5 binds to Ime2 (Fig 5C and D), the only meiosis‐specific regulator required for reconstituting Spc72 removal in mitotic cells (Fig 7). Cdc5 might therefore recruit Ime2 to SPBs. Furthermore, Cdc5 has been proposed to activate Ime2 (Schindler & Winter, 2006), and Ime2 undergoes an electrophoretic mobility shift that depends on Cdc5 activity (Fig 5E). Thus, it might be the Cdc5‐Ime2 complex that releases Spc72 from SPBs, for instance, by phosphorylating Spc72 itself or its binding partner at the SPB, the centriolin Nud1 (Gruneberg *et al*, 2000; Gordon *et al*, 2006). An alternative mechanism is suggested by the finding that removal of Spc72 from SPBs correlates with and might even be caused by the degradation of the cytoplasmic pool of Spc72. Accordingly, Cdc5‐Ime2 might phosphorylate Spc72 for recognition by a ubiquitin‐ligase that mediates Spc72's degradation. The translational repressor Rim4 and the mitochondria‐plasma membrane tether Num1 are examples of cytoplasmic proteins degraded in an Ime2‐dependent manner (Berchowitz *et al*, 2013; Sawyer *et al*, 2019).

### The function of Spo13/Meikin

How does Spo13 control processes as diverse as kinetochore orientation and spore formation? While Spo13 and its orthologues are poorly conserved, they share the ability to bind Cdc5/Plk1 (Matos *et al*, 2008; Kim *et al*, 2015; Maier *et al*, 2021). Indeed, Spo13/Meikin has been proposed to promote the meiosis I‐specific behavior of centromeres by recruiting Cdc5/Plk1 to kinetochores (Kim *et al*, 2015; Galander *et al*, 2019; Maier *et al*, 2021). However, Spo13 has a different function in the control of spore formation: First, this function does not require intact kinetochores (Appendix Fig S4). Second, by blocking premature sporulation, Spo13 plays an inhibitory role. A simple hypothesis is that Spo13's binding to the phosphopeptide‐binding cleft of the PBD prevents Cdc5 from engaging its substrates. For instance, Spo13 might prevent Cdc5 from activating Ime2. However, the levels of Spo13 at metaphase I are much lower than those of Cdc5 (Fig 5B), making it unlikely that Spo13 can inhibit the entire pool of Cdc5. We therefore propose that Cdc5 exists as a free and a Spo13‐bound kinase. Indeed, several meiosis I‐specific processes require Cdc5 but not Spo13. Examples include the resolution of double‐Holliday junctions (Klapholz & Esposito, 1980b; Clyne *et al*, 2003), disassembly of the synaptonemal complex (Katis *et al*, 2004), and activation of APC/C^Cdc20^ (Shonn *et al*, 2002; Clyne *et al*, 2003; Lee & Amon, 2003). On the other hand, Cdc5 activity and Spo13 are both required for the inhibition of MP assembly (Fig 8C and D) and the hyperphosphorylation of the monopolin subunit Lrs4 (Katis *et al*, 2004; Matos *et al*, 2008). Furthermore, the hyperphosphorylation of the cyclin Clb1 at metaphase I depends on Cdc5 activity (Attner *et al*, 2013; Tibbles *et al*, 2013) and is enhanced, rather than repressed, by non‐degradable Spo13 (Fig EV2). The idea of Cdc5‐Spo13 as an active kinase makes at least two predictions: First, Spo13 should change Cdc5's target sites, so that Cdc5‐Spo13 phosphorylates inhibitory sites on Ime2, while free Cdc5 modifies activating sites. Second, Cdc5‐Spo13 should recognize its substrates while Spo13 occupies the phosphopeptide‐binding cleft of the PBD. Indeed, the PBD has an additional interaction surface, which can bind substrates independently of the phosphopeptide‐binding cleft (Chen & Weinreich, 2010; Almawi *et al*, 2020). Spo13 might force Cdc5 to adopt this noncanonical mechanism of substrate recognition, and it might provide additional contacts to the substrate.

Spo13 and its orthologues have been regarded as kinetochore proteins that promote the meiosis I‐specific behavior of centromeres. We show, however, that Spo13 has additional functions, being able to control events at the centrosome in a kinetochore‐independent manner. Spo13/Meikin might play a more general role in giving meiosis I and meiosis II their distinct properties.

## Material and Methods

### Construction of *Saccharomyces cerevisiae* strains

Experiments were performed with diploid SK1 strains generated by mating of the appropriate haploids. Genotypes including the names of the fluorescent proteins are listed in Appendix Table S1. In the main text, all green and red fluorescent proteins are abbreviated as GFP and RFP, respectively. We used *CDC20‐mAR* strains for arrest/release at metaphase I (Arguello‐Miranda *et al*, 2017) and *cdc20^ts^‐mAR* strains for arrest at metaphase II (Mengoli *et al*, 2021). Estradiol‐inducible expression from the *GAL* promoter (called *P_EST_* herein) was achieved with a Gal4‐estrogen receptor fusion controlled by the *GPD1* promoter (Benjamin *et al*, 2003). To deplete meiotic cells of Cdc5 or Cdc20, the endogenous promoters were replaced with the mitosis‐specific promoters of *SCC1* (Clyne *et al*, 2003) or *HSL1* (Okaz *et al*, 2012), respectively. SK1 strains containing analogue‐sensitive *cdc28‐as1* (F88G; Oelschlaegel *et al*, 2005), *cdc5‐as* (L158G; Okaz *et al*, 2012), *ime2‐as* (M146G; Benjamin *et al*, 2003), or *hrr25‐as* (I82G; Petronczki *et al*, 2006) have been described. To create SK1 *cdc28‐as2* cells, the ts‐mutant SK1 *cdc28‐4* (H128Y) was transformed with *cdc28‐F88A* DNA (Bishop *et al*, 2000) followed by selection for growth at 37°C. In *IME2‐ΔC*, the C‐terminal 241 residues have been replaced with an Ha3 tag (Sari *et al*, 2008). The alleles *spc72Δ leu2::SPC72*, *spc72Δ leu2::spc72‐7* (Knop & Strasser, 2000), and *cdc20‐3* (Shirayama *et al*, 1998) were backcrossed into SK1 ≥8 times. Clb1‐mDK (Okaz *et al*, 2012), Cdc5's PBD (357–705), and the corresponding FAA mutant (W517F, V518A, L530A; Song *et al*, 2000) were expressed using the *P_EST_* system. The alleles *spo13‐mD* (L26A; Sullivan & Morgan, 2007) and *spo13‐m2* (S132T, S134T; Matos *et al*, 2008) have been described. In *spo13‐10A* and *spo13‐10D*, all S/T‐P motifs have been mutated to A‐P and D‐P, respectively. For auxin‐inducible degradation (Nishimura *et al*, 2009), *PDS1* was C‐terminally tagged with AID* carrying a 30‐residue linker (Morawska & Ulrich, 2013; Mengoli *et al*, 2021) and crossed into strains harboring a *P_CUP1_‐OsTIR1‐myc3* plasmid at the *ura3* locus (a gift from Neil Hunter; Tang *et al*, 2015). MP proteins were detected in strains heterozygous for *MPC54‐eGFP*, *MPC70‐eGFP* (Knop & Strasser, 2000), or *SPO74* tagged with super‐folder GFP (sfGFP; Pedelacq *et al*, 2006). Spc72 was tagged with eGFP (Knop & Strasser, 2000) or mScarlet (Bindels *et al*, 2017), and Pds1 was tagged with mNeonGreen (Shaner *et al*, 2013). Microtubules were labeled with mScarlet‐I‐Tub1, nuclei with TetR‐tdTomato (Matos *et al*, 2008), and PSMs with mRFP‐Spo20^51–91^ expressed from pRS426‐R20 (a gift from Aaron Neiman; Diamond *et al*, 2009). Heterozygous *NDT80‐sfGFP* was used for monitoring entry into meiosis (Fig 8A and C). To assess spore viability, 36 tetrads per strain were dissected on YPD plates.

### Meiotic and mitotic cultures

Meiosis was induced at 30°C as described (Arguello‐Miranda *et al*, 2017). Briefly, cells from YP‐glycerol plates were uniformly spread on YPD plates and grown for 24 h. Cells were inoculated into liquid YP‐acetate medium and grown for 12 h until cells enter a transient G1 arrest. Cells were washed with sporulation medium (SPM, 2% K‐acetate), inoculated into 100 ml of SPM in a 2.8 l‐Fernbach flask, and rotated on an orbital shaker. *spc72‐7* and control strains were induced to enter meiosis at 24°C, using YP‐2% raffinose plates for initial growth. Cultures in SPM were shifted to 36°C by placing the flasks into a shaking water bath. To arrest *cdc20^ts^‐mAR* strains at metaphase II (Fig 9B and C), cells were allowed to progress to the metaphase I‐arrest at 25°C. At 8 h in SPM, cells were released into anaphase I by addition of CuSO_4_ (10 μM) and shifted to 37°C at 50 min after release. Expression from the *P_EST_* promoter was induced with estradiol (10 μM). Analogue‐sensitive kinases were inhibited with 1NM‐PP1 (*cdc28‐as1* and *hrr25‐as*, 5 μM; *cdc28‐as2*, 10 μM), 1Na‐PP1 (*ime2‐as*, 20 μM), or CMK (*cdc5‐as*, 20 μM). To inhibit and reactivate Cdk1, *cdc28‐as2* cells treated with 1Na‐PP1 (10 μM) were collected by filtration (Whatman 10400772, 3 μm pore size), washed with conditioned SPM (cSPM, 10 culture volumes, obtained from cultures in SPM without inhibitor), and inoculated into cSPM. Indole‐3‐acetic acid (IAA, 2 mM) was used for auxin‐inducible degradation. For mitotic cultures, diploid SK1 *cdc20‐3* strains were grown at 24°C in SC‐2% raffinose medium and shifted to 36°C at the indicated time.

### Live‐cell imaging

Imaging was performed as described (Mengoli *et al*, 2021). Meiotic cells were applied to an 8‐well glass‐bottom µ‐slide (ibidi, Gräfelfing, Germany) coated with Concanavalin A in 250 µl of SPM per well to give a density of 20–30 cells per field of view. Mitotic cells were applied in SC‐raffinose medium to give ~ 10 cells per field of view. Drugs and/or CuSO_4_ were added to 125 µl of medium. For temperature shift experiments (Figs 3A, 7 and EV4), cells were applied at 24°C to the µ‐slide, which was transferred to the microscope equilibrated to 36°C. For Fig 8D, we used the Vaheat micro‐heating system (Interherence, Erlangen, Germany). Cells were applied to an SmS‐R culture chamber in 150 µl of SPM at 24°C and shifted from 24 to 36°C (over 4 min) during imaging. 10 min before the shift, the environmental chamber was set to 36°C. CMK was added in 50 µl of SPM. Cells were imaged on a DeltaVision Elite system consisting of an Olympus IX71 microscope with autofocus (Ultimate Focus) and solid‐state illumination (InsightISS) attenuated by an ND filter (12.6% T), a PlanApo 100×/1.4 NA oil objective, DeltaVision filter sets, a CoolSnap HQ2 camera, and an environmental chamber for temperature control. We acquired Z‐stacks (8 × 1 μm) in the GFP and the RFP channel every 10 min for 14 h. Z‐stacks were deconvolved and projected to a single 2D image with softWoRx 6.1 (standard projection). Time‐lapse series were produced in Fiji (http://fiji.sc/). The frame width is 5 μm, unless stated otherwise. For quantification, meiotic events of individual cells were aligned to a reference event (e.g., SPB separation at metaphase I) set to *t* = 0 in each cell. Percentages of other events were calculated at 10‐min intervals relative to the reference event using Microsoft Excel.

### Transmission electron microscopy

Transmission electron microscopy was performed essentially as described (Byers & Goetsch, 1991). Briefly, cells (4 ml) were treated with 10 mM DTT for 5 min and fixed overnight at 4°C in 0.2 M Na‐cacodylate buffer pH 7.4 with 3% glutaraldehyde. Cells were washed with 0.2 M phosphate citrate buffer pH 5.8 and spheroplasted using zymolyase 100T (0.2 mg/ml, amsbio). Spheroplasts were washed with 0.1 M Na‐acetate pH 6.1 and treated with 2% osmium tetroxide in Na‐acetate for 15 min. Cells were washed with water, pelleted, and overlaid with 1% aqueous uranyl acetate for 60 min. After washing with water, cells were dehydrated through an ethanol series (15, 50, 75, 95, 100%) and incubated in 2:1 (v/v) and 1:1 solutions of ethanol plus Spurr's resin (Electron Microscopy Sciences) and finally in Spurr's resin only. Cells were pelleted, transferred to BEEM capsules, and placed in a 70°C‐oven for 24 h. Ultrathin (50 nm) sections were cut with a 45°‐diamond knife (DiATOME) on a Leica EM UC6 Ultramicrotome and mounted on Copper‐Slotgrids coated with Formvar (Electron Microscopy Sciences). Images were acquired on a JEOL JEM‐1230 transmission electron microscope at 80 kV with a Gatan Orius SC1000 camera controlled by Gatan DigitalMicrograph software.

### Analysis of proteins

To immunoprecipitate proteins tagged with Ha3, protein extracts (0.5 ml, 16 mg/ml) (Petronczki *et al*, 2006) were incubated with protein A‐agarose (Roche) and mouse monoclonal antibodies to Ha (clone 12CA5, Roche). To follow protein levels, cells were broken with glass beads in 10% trichloroacetic acid (Matos *et al*, 2008). Protein samples (60 μg) were separated in SDS–8% PAA gels and transferred by semi‐dry blotting to Immobilon P membranes (Millipore). Membranes were horizontally cut into 2–3 slices and incubated with primary antibodies for 2 h. Primary antibodies had been raised in rabbits to Ama1 (Oelschlaegel *et al*, 2005; 1:2,000), Cdc20 (Camasses *et al*, 2003; 1:2,000), Cdh1 (Schwickart *et al*, 2004; 1:5,000), Ndt80 (a gift from Kirsten Benjamin; Benjamin *et al*, 2003); 1:5,000), Pds1 (Katis *et al*, 2010; 1:500), Rec8 (Petronczki *et al*, 2006; 1:10,000), Cdc5 (1:5,000), Dbf4 (1:5,000), and Spo13 (1:5,000) (Matos *et al*, 2008), and Spc72 (a gift from Michael Knop and Elmar Schiebel; Knop & Schiebel, 1998; 1:3,000). We used goat antibodies (Santa Cruz) for Clb1 (sc‐7647; 1:300) and Ime2 (sc‐26444; 1:300). Mouse monoclonal antibodies were used for detection of GFP (clones 7.1 and 13.1, Roche; 1:4,000) and Pgk1 (clone 22C5D8, Invitrogen; 1:20,000). Ha was detected with a rat monoclonal antibody (clone 3F10, Roche; 1:2,000). HRP‐conjugated secondary antibodies were detected by ECL (GE Healthcare) on X‐ray films, which were digitized using an Epson Perfection V750 Pro scanner. To determine the Cdc5‐ha3/Ha3‐Spo13 ratio, immunoblots were analyzed by near‐infrared fluorescence imaging (LI‐COR Odyssey CLx), using IRDye 800CW secondary antibodies (Li‐COR).

### Statistical analysis

Proportions were compared with Fisher's exact test (two‐tailed). Distributions of the times of Spc72 removal or Mpc70 loading tend to be symmetrical or slightly skewed. Thus, we used the unequal variance *t*‐test (Welch's *t*‐test, unpaired, two‐tailed) to compare means and provide confidence intervals for the difference between group means. With a balanced design and a sample size of *n* = 80–100 per group, Welch's *t*‐test is robust against moderate deviations from normality. Calculations were performed in GraphPad Prism 9.

## Author contributions

**Tugce Oz:** Formal analysis; Investigation; Writing ‐ review & editing. **Valentina Mengoli:** Formal analysis; Investigation; Writing ‐ review & editing. **Julie Rojas:** Formal analysis; Investigation; Writing ‐ review & editing. **Katarzyna Jonak:** Formal analysis; Investigation; Writing ‐ review & editing. **Marianne Braun:** Formal analysis; Investigation. **Ievgeniia Zagoriy:** Formal analysis; Investigation. **Wolfgang Zachariae:** Conceptualization; Formal analysis; Supervision; Writing ‐ original draft.

In addition to the CRediT author contributions listed above, the contributions in detail are:

TO, VM, JR, KJ, and IZ performed experiments. MB prepared TEM samples. TO, VM, JR, KJ, and WZ designed experiments. TO, VM, JR, KJ, IZ, and WZ analyzed data. WZ supervised the project. WZ wrote the manuscript with input from TO, VM, JR, and KJ.

## Disclosure statement and competing interests

The authors declare that they have no conflict of interest.

## Supporting information



Appendix

Expanded View Figures PDF

Source Data for Expanded View/Appendix

Source Data for Figure 5

Source Data for Figure 6

## Data Availability

No datasets were deposited in public repositories.
