# Supplementary material for: The Spo13/Meikin pathway confines the onset of gamete differentiation to meiosis II in yeast
Source: EMBO J. 2022 Jan 13;41(4):e109446. doi: 10.15252/embj.2021109446 (PMC8844990; doi:10.15252/embj.2021109446)
Supplement: Supplementary file 1 — Appendix [file EMBJ-41-e109446-s004.pdf]

APPENDIX

The Spo13/Meikin Pathway Confines the Onset of Gamete Differentiation to Meiosis II in Yeast

Tugce Oz, Valentina Mengoli, Julie Rojas, Katarzyna Jonak, Marianne Braun, Ievgeniia Zagoriy, and Wolfgang Zachariae

TABLE OF CONTENTS

Appendix Figure S1. Exchange of Spc72 for Mpc70 at SPBs in control and *spo13Δ* cells

Appendix Figure S2. Spo13 is not cleaved upon activation of separase

Appendix Figure S3. Analysis of P_{HSL1}-*CDC20 ama1Δ* cells lacking Clb3 or Clb4

Appendix Figure S4. Control of spore formation by Spo13 does not require intact kinetochores

Appendix Figure S5. Spc72 removal requires the activities of Cdc5 and Ime2

Appendix Figure S6. MP assembly does not require Cdc5 or Ime2 activity at metaphase II

Appendix Table S1. *Saccharomyces cerevisiae* SK1 strains used in this study

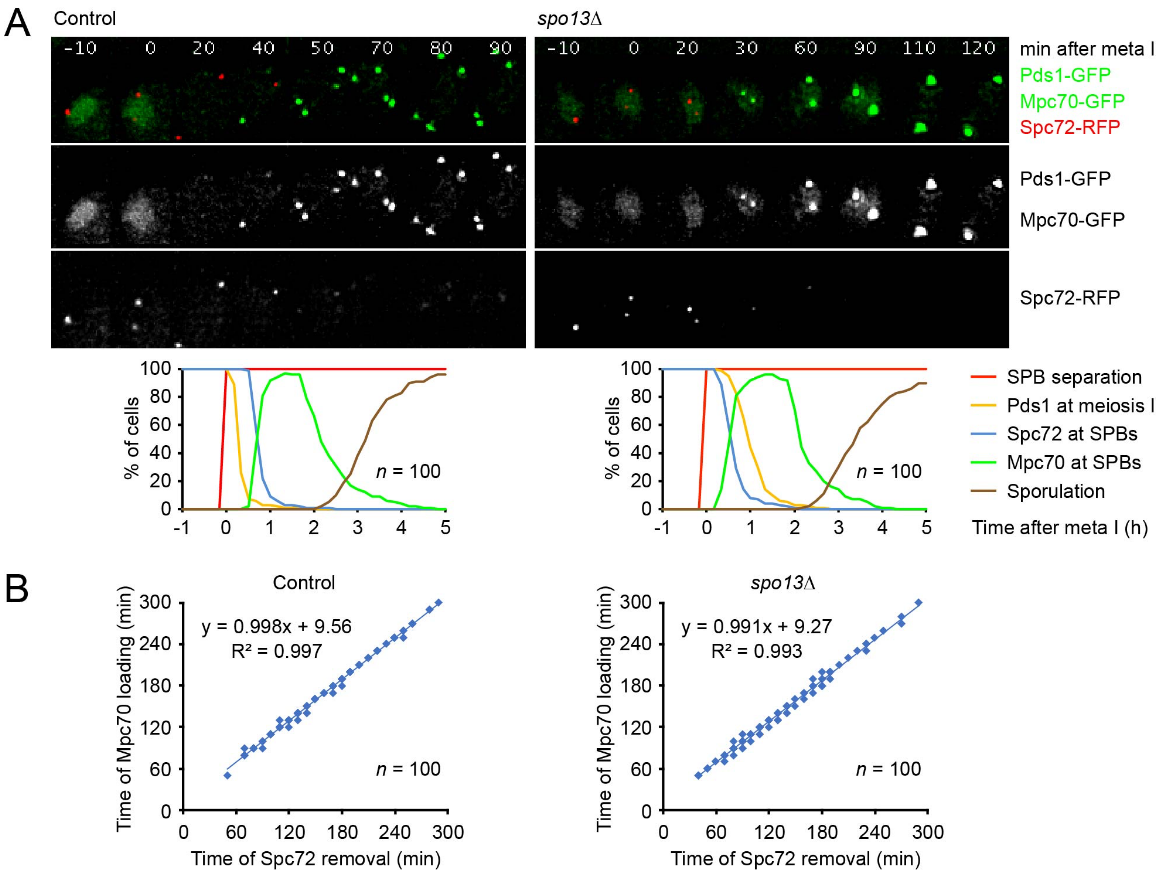

Appendix Figure S1. Exchange of Spc72 for Mpc70 at SPBs in control and *spo13Δ* cells

A. Imaging of Pds1-GFP, Mpc70-GFP, and Spc72-RFP in control and *spo13Δ* cells. Top, time-lapse series. Bottom, meiotic events were quantified in cells synchronized in silico to SPB separation at entry into metaphase I (t = 0). In *spo13Δ* cells, Pds1 degradation is delayed by 42 min (95% CI, 36-48; $P < 0.0001$), whereas Spc72 removal and Mpc70 loading are advanced by 8.7 min (95% CI, 3.6-13.8; $P = 0.0009$) and 9.8 min (95% CI, 4.7-14.9; $P = 0.0002$), respectively. Group means were compared using Welch's *t*-test.

B. Mpc70 loading correlates with Spc72 removal in control and *spo13Δ* cells from (A). Times were measured from the start of imaging at 3.5 h in SPM. The regression lines show similar slopes ($P = 0.53$) and comparable y-intercepts ($P = 0.045$; ANCOVA).

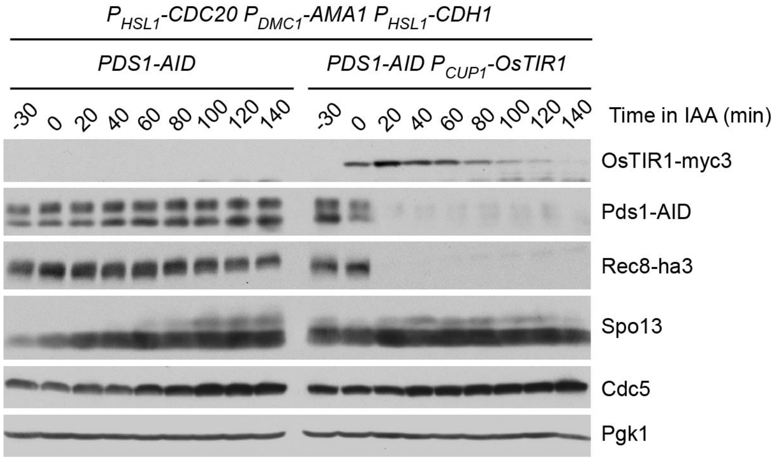

Appendix Figure S2. Spo13 is not cleaved upon activation of separase

Activation of separase in metaphase I-arrested cells by auxin-inducible degradation of Pds1 causes cleavage of Rec8 but does not affect Spo13. *P_{HSL1}-CDC20 P_{DMC1}-AMA1 P_{HSL1}-CDH1* cells containing *PDS1-AID* or *PDS1-AID* plus *P_{CUP1}-OsTIR1-myc3* were arrested at metaphase I by depletion of APC/C activators. At 8 h in SPM, cells were treated with $CuSO_4$ to induce expression of the plant F-box protein OsTIR1. At 8.5 h in SPM (*t* = 0), the auxin IAA was added to activate separase through the degradation of Pds1-AID. Proteins were detected in whole-cell extracts by immunoblotting.

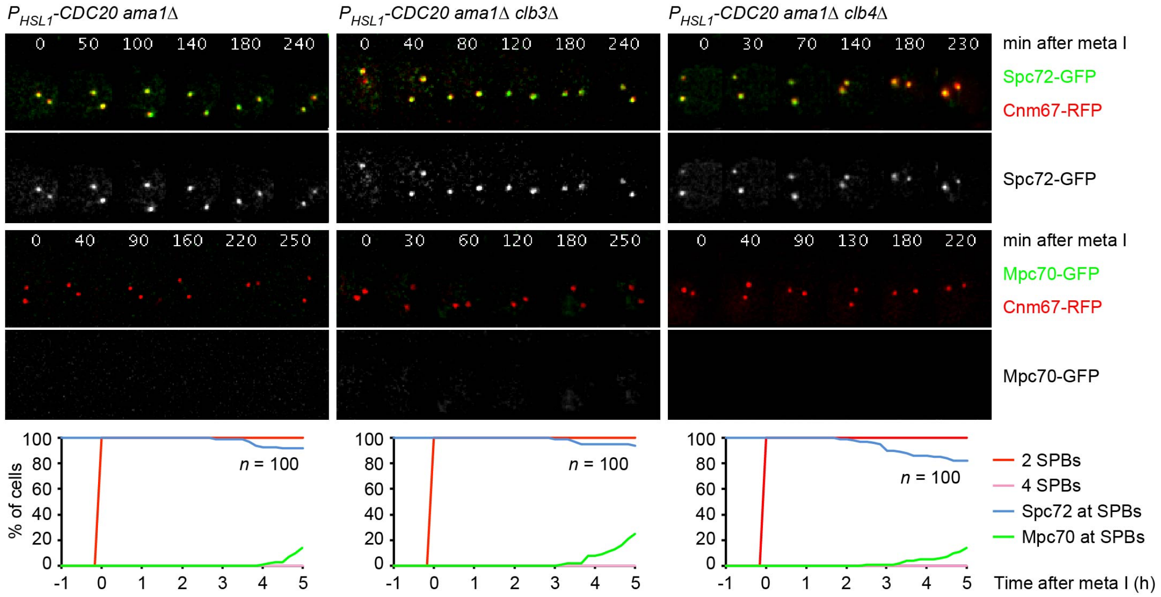

Appendix Figure S3. Analysis of *P_{HSL1}-CDC20 ama1Δ* cells lacking Clb3 or Clb4

Imaging of SPBs (Cnm67-RFP) and Spc72-GFP or Mpc70-GFP in *P_{HSL1}-CDC20 ama1Δ* control cells and cells lacking Clb3 or Clb4. Top, time-lapse series. Bottom, meiotic events were quantified in cells synchronized in silico to SPB separation at entry into metaphase I ($t = 0$). Graphs show overlays of *SPC72-GFP* and *MPC70-GFP* strains.

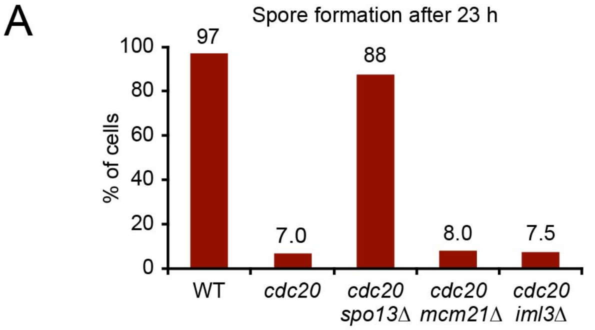

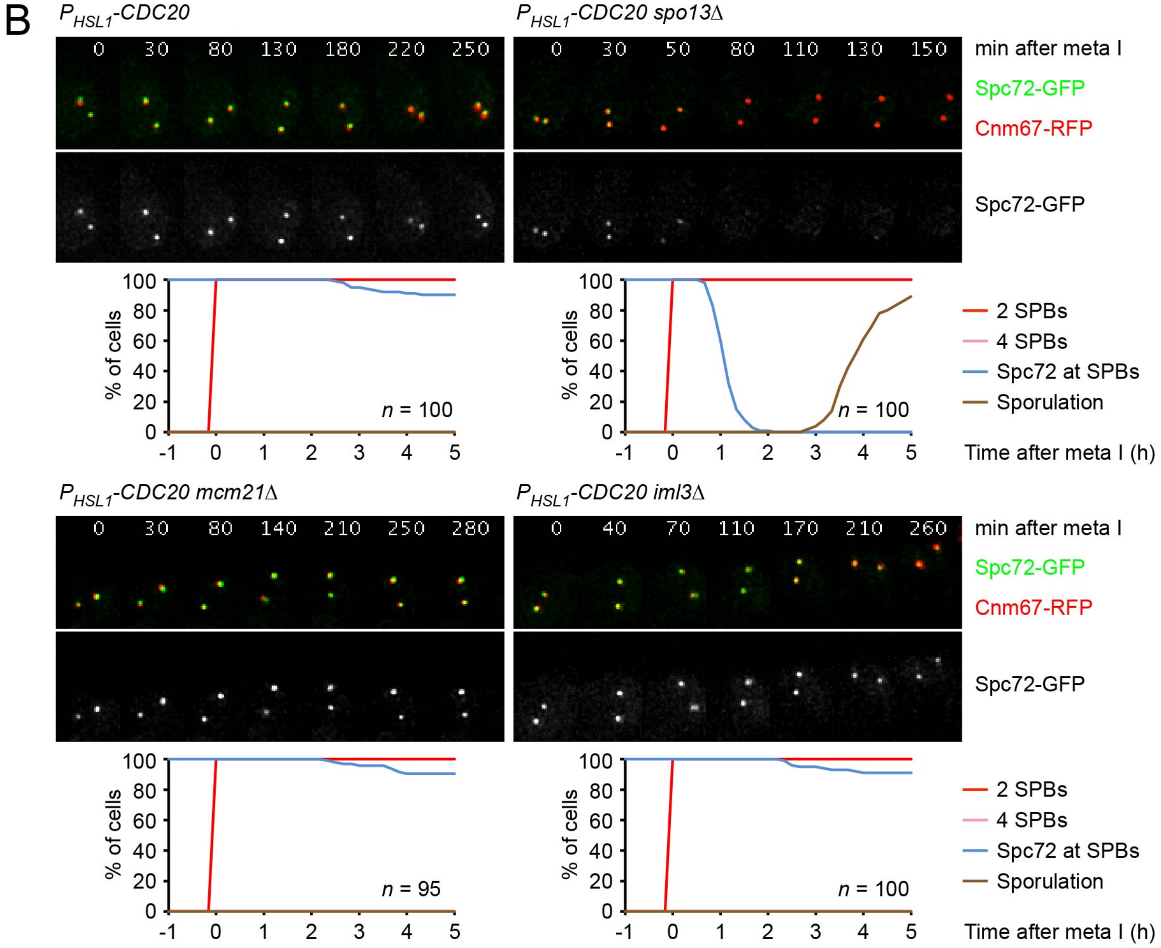

Appendix Figure S4. Control of spore formation by Spo13 does not require intact kinetochores

A. Quantification of spore formation in the wild-type (WT) and the indicated P_{HSL1}-*CDC20* strains at 23 h in SPM. Data are mean values of two cultures (*n* = 200 cells per culture).

B. Imaging of SPBs (Cnm67-RFP) and Spc72-GFP in P_{HSL1}-*CDC20* control cells and cells lacking Spo13, Mcm21, or Iml3. Top, time-lapse series. Bottom, Spc72's presence at SPBs was quantified in cells synchronized in silico to SPB separation at entry into metaphase I (*t* = 0). While P_{HSL1}-*CDC20 mcm21Δ* and P_{HSL1}-*CDC20 iml3Δ* cells are defective in kinetochore assembly, they retain Spc72 at SPBs. Data are representative of two independent experiments.

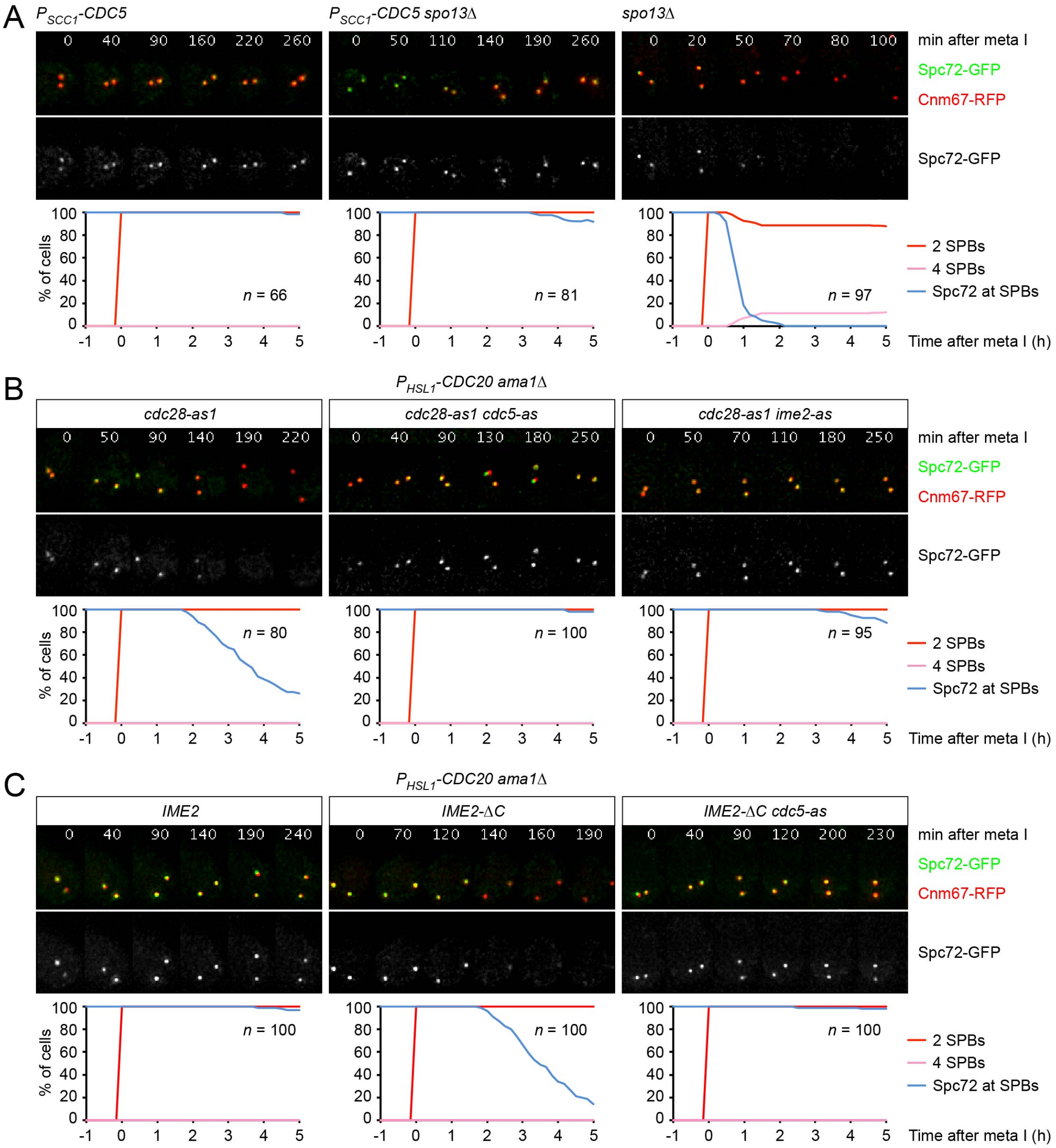

Appendix Figure S5. Spc72 removal requires the activities of Cdc5 and Ime2

A. Imaging of SPBs (Cnm67-RFP) and Spc72-GFP in cells lacking Cdc5 (*P_{SCC1}-CDC5*) and/or Spo13. Top, time-lapse series. Bottom, Spc72's presence at SPBs was quantified in cells synchronized in silico to SPB separation at entry into metaphase I (*t* = 0).

B. Imaging of SPBs (Cnm67-RFP) and Spc72-GFP in *P_{HSL1}-CDC20 ama1Δ cdc28-as1* control cells and cells containing *cdc5-as* or *ime2-as*. At metaphase I (8 h in SPM), cells were treated with 1NM-PP1 (to inhibit Cdc28-as1) plus either CMK (to inhibit Cdc5-as) or 1Na-PP1 (to inhibit Ime2-as). Spc72's presence at SPBs was analyzed as in (A).

C. Imaging of SPBs (Cnm67-RFP) and Spc72-GFP in *P_{HSL1}-CDC20 ama1Δ* cells containing wild-type *IME2*, *IME2-ΔC*, or *IME2-ΔC* plus *cdc5-as*. Cdc5-as was inhibited with CMK at 6.5 h in SPM. Spc72's presence at SPBs was analyzed as in (A).

Data information: data in (B) and (C) are representative of two independent experiments.

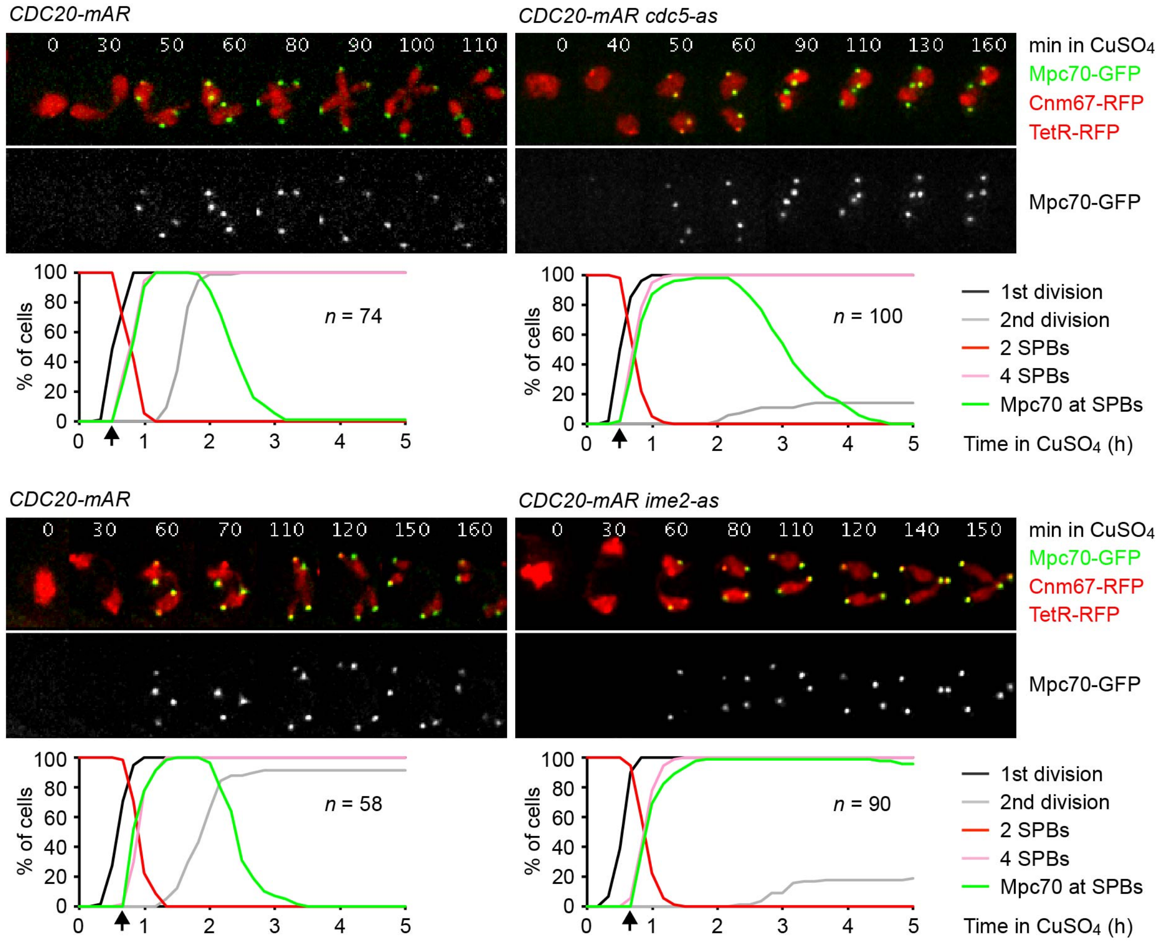

Appendix Figure S6. MP assembly does not require Cdc5 or Ime2 activity at metaphase II

CDC20-mAR control cells and cells containing *cdc5-as* (top) or *ime2-as* (bottom) were released from the metaphase I-arrest with $CuSO_4$ at 7 h in SPM (t = 0). At anaphase I (arrows), cells were treated with CMK (to inhibit Cdc5-as) or 1Na-PP1 (to inhibit Ime2-as). Panels, time-lapse series from the imaging of Mpc70-GFP, SPBs (Cnm67-RFP), and nuclei (TetR-RFP). Graphs, quantification of meiotic events.

Appendix Table S1. ***Saccharomyces cerevisiae*** **SK1 Strains Used in this Study**

Figure	Strain[1]	Genotype[2]
1A	Z17009	*PDS1myc18::KlTRP1*
1A	Z32767	*PDS1myc18::KlTRP1 cdc20::*P_{HSL1}*-CDC20-HphMX4*
1A	Z32771	*PDS1myc18::KlTRP1 cdc20::*P_{HSL1}*-CDC20-HphMX4 spo13Δ::HIS3MX6*
1A	Z38457	*PDS1myc18::KlTRP1 cdc20::*P_{HSL1}*-CDC20-HphMX4 spo13Δ::HIS3MX6::spo13-m2::HphMX4*
1A	Z32768	*PDS1myc18::KlTRP1 cdc20::*P_{HSL1}*-CDC20-HphMX4 clb1Δ::BleMX4*
1A	Z35062	*cdc20::*P_{HSL1}*-CDC20-HphMX4 clb3Δ::TRP1*
1A	Z32769	*PDS1myc18::KlTRP1 cdc20::*P_{SCC1}*-CDC20-KanMX4 clb4Δ::KanMX4*
1B	Z35411	*his3::*P_{HIS3}*-eGFP-TUB1::HIS3 leu2::*P_{URA3}*-tetR-tdTomato::LEU2*
1B	Z35412	*his3::*P_{HIS3}*-eGFP-TUB1::HIS3 leu2::*P_{URA3}*-tetR-tdTomato::LEU2 cdc20::*P_{HSL1}*-CDC20-HphMX4*
1B	Z35414	*his3::*P_{HIS3}*-eGFP-TUB1::HIS3 leu2::*P_{URA3}*-tetR-tdTomato::LEU2 cdc20::*P_{HSL1}*-CDC20-HphMX4 spo13Δ::HIS3MX6*
1B	Z35415	*his3::*P_{HIS3}*-eGFP-TUB1::HIS3 leu2::*P_{URA3}*-tetR-tdTomato::LEU2 cdc20::*P_{HSL1}*-CDC20-HphMX4 clb1Δ::KanMX4*
1C	Z33199	*SPC72-eGFP::KanMX4 CNM67-tdTomato::NatMX4*
1C	Z32054	*SPC72-eGFP::KanMX4 CNM67-tdTomato::NatMX4 cdc20::*P_{HSL1}*-CDC20-HphMX4*
1C	Z21998	*MPC70/MPC70-eGFP::KanMX4 CNM67-tdTomato::NatMX4*
1C	Z35190	*MPC70/MPC70-eGFP::KanMX4 CNM67-tdTomato::NatMX4 cdc20::*P_{HSL1}*-CDC20-HphMX4*
1D	Z35268	*SPC72-eGFP::KlTRP1 CNM67-tdTomato::NatMX4 cdc20::*P_{HSL1}*-CDC20-HphMX4 spo13Δ::BleMX4*
1D	Z35266	*SPC72-eGFP::KlTRP1 CNM67-tdTomato::NatMX4 cdc20::*P_{HSL1}*-CDC20-HphMX4 spo13Δ::HIS3MX6::spo13-m2::HphMX4*
1D	Z35269	*SPC72-eGFP::KlTRP1 CNM67-tdTomato::NatMX4 cdc20::*P_{HSL1}*-CDC20-HphMX4 clb1Δ::BleMX4*
1D	Z35455	*MPC70/MPC70-eGFP::KanMX4 CNM67-tdTomato::NatMX4 cdc20::*P_{HSL1}*-CDC20-HphMX4 spo13Δ::BleMX4*
1D	Z35189	*MPC70/MPC70-eGFP::KanMX4 CNM67-tdTomato::NatMX4 cdc20::*P_{HSL1}*-CDC20-HphMX4 spo13Δ::HIS3MX6::spo13-m2::HphMX4*
1D	Z35456	*MPC70/MPC70-eGFP::KanMX4 CNM67-tdTomato::NatMX4 cdc20::*P_{HSL1}*-CDC20-HphMX4 clb1Δ::BleMX4*
2A	Z32389	*SPC72-eGFP::KanMX4 CNM67-tdTomato::NatMX4 cdc20::*P_{HSL1}*-CDC20-HphMX4 ama1Δ::CaURA3*
2A	Z35509	*SPC72-eGFP::KanMX4 CNM67-tdTomato::NatMX4 cdc20::*P_{HSL1}*-CDC20-HphMX4 ama1Δ::CaURA3 spo13Δ::HIS3MX6*
2A	Z37925	*SPC72-eGFP::KlTRP1 CNM67-tdTomato::NatMX4 cdc20::*P_{HSL1}*-CDC20-HphMX4 ama1Δ::CaURA3 spo13Δ::HIS3MX6::spo13-m2::HphMX4*
2A	Z32908	*MPC70/MPC70-eGFP::KanMX4 CNM67-tdTomato::NatMX4 cdc20::*P_{HSL1}*-CDC20-HphMX4 ama1Δ::CaURA3*
2A	Z35230	*MPC70/MPC70-eGFP::KanMX4 CNM67-tdTomato::NatMX4 cdc20::*P_{HSL1}*-CDC20-HphMX4 ama1Δ::CaURA3 spo13Δ::BleMX4*
2A	Z35229	*MPC70/MPC70-eGFP::KanMX4 CNM67-tdTomato::NatMX4 cdc20::*P_{HSL1}*-CDC20-HphMX4 ama1Δ::CaURA3 spo13Δ::HIS3MX6::spo13-m2::HphMX4*
2B	Z36062	*cdc20::*P_{HSL1}*-CDC20-HphMX4 ama1Δ::NatMX4 cdh1::*P_{HSL1}*-CDH1-BleMX4*
2B	Z36065	*cdc20::*P_{HSL1}*-CDC20-HphMX4 ama1Δ::CaURA3 cdh1::*P_{HSL1}*-CDH1-HIS3MX6 spo13Δ::BleMX4*

2B	Z23218	*cdc20::P_{CLB2}-CDC20-HphMX4 ura3::P_{CUP1}-CDC20-URA3 hrr25Δ::KanMX4::HRR25-HIS3*
2B	Z29809	*cdc20::P_{CLB2}-CDC20-HphMX4 ura3::P_{CUP1}-CDC20-URA3 mpc54Δ::AurCMX4 mpc70Δ::BleMX4*
2C	Z32389	*SPC72-eGFP::KanMX4 CNM67-tdTomato::NatMX4 cdc20::P_{HSL1}-CDC20-HphMX4 ama1Δ::CaURA3*
2C	Z32513	*SPC72-eGFP::KanMX4 CNM67-tdTomato::NatMX4 cdc20::P_{HSL1}-CDC20-HphMX4 ama1Δ::CaURA3 clb1Δ::BleMX4*
2C	Z32388	*SPC72-eGFP::KanMX4 CNM67-tdTomato::NatMX4 cdc20::P_{HSL1}-CDC20-HphMX4 ama1Δ::CaURA3 cdc28-as1*
2C	Z32908	*MPC70/MPC70-eGFP::KanMX4 CNM67-tdTomato::NatMX4 cdc20::P_{HSL1}-CDC20-HphMX4 ama1Δ::CaURA3*
2C	Z32907	*MPC70/MPC70-eGFP::KanMX4 CNM67-tdTomato::NatMX4 cdc20::P_{HSL1}-CDC20-HphMX4 ama1Δ::CaURA3 clb1Δ::BleMX4*
2C	Z32545	*MPC70/MPC70-eGFP::KanMX4 CNM67-tdTomato::NatMX4 cdc20::P_{HSL1}-CDC20-HphMX4 ama1Δ::CaURA3 cdc28-as1*
3A	Z36338	*MPC70/MPC70-eGFP::KanMX4 CNM67-tdTomato::NatMX4 cdc20::P_{HSL1}-CDC20-HphMX4 ama1Δ::CaURA3 spc72Δ::KanMX4 leu2::SPC72-LEU2*
3A	Z36513	*MPC70/MPC70-eGFP::KanMX4 CNM67-tdTomato::NatMX4 cdc20::P_{HSL1}-CDC20-HphMX4 ama1Δ::CaURA3 spc72Δ::KanMX4 leu2::spc72-7-LEU2*
3A	Z36585	*MPC70/MPC70-eGFP::KanMX4 CNM67-tdTomato::NatMX4 cdc20::P_{HSL1}-CDC20-HphMX4 ama1Δ::CaURA3 spc72Δ::KanMX4 leu2::spc72-7-LEU2 hrr25Δ::KanMX4::hrr25-as-HIS3*
3B	Z36546	*cdc20::P_{HSL1}-CDC20-HphMX4 ama1Δ::CaURA3 spc72Δ::KanMX4 leu2::SPC72-LEU2*
3B	Z36545	*cdc20::P_{HSL1}-CDC20-HphMX4 ama1Δ::CaURA3 spc72Δ::KanMX4 leu2::spc72-7-LEU2*
3C	Z35213	*MPC70/MPC70-eGFP::KanMX4 CNM67-tdTomato::NatMX4 cdc20::P_{HSL1}-CDC20-HphMX4 ama1Δ::CaURA3 spo13Δ::BleMX4 his3::P_{GPD}-$GAL4^{484}$-ER-HIS3*
3C	Z35397	*MPC70/MPC70-eGFP::KanMX4 CNM67-tdTomato::NatMX4 cdc20::P_{HSL1}-CDC20-HphMX4 ama1Δ::CaURA3 spo13Δ::BleMX4 his3::P_{GPD}-$GAL4^{484}$-ER-HIS3 leu2::P_{GAL1}-SPC72-LEU2*
4A	Z33847	*SPC72-eGFP::KanMX4 CNM67-tdTomato::NatMX4 his3::P_{GPD}-$GAL4^{484}$-ER-HIS3 spo13Δ::HIS3MX6::spo13-mD::HphMX4*
4A	Z33846	*SPC72-eGFP::KanMX4 CNM67-tdTomato::NatMX4 his3::P_{GPD}-$GAL4^{484}$-ER-HIS3 leu2/leu2::P_{GAL1}-clb1-mDK-LEU2 spo13Δ::HIS3MX6::spo13-mD::HphMX4*
4A	Z33845	*SPC72-eGFP::KanMX4 CNM67-tdTomato::NatMX4 his3::P_{GPD}-$GAL4^{484}$-ER-HIS3 leu2/leu2::P_{GAL1}-clb1-mDK-LEU2*
4A	Z35032	*MPC70/MPC70-eGFP::KanMX4 CNM67-tdTomato::NatMX4 his3::P_{GPD}-$GAL4^{484}$-ER-HIS3 spo13Δ::HIS3MX6::spo13-mD::HphMX4*
4A	Z35033	*MPC70/MPC70-eGFP::KanMX4 CNM67-tdTomato::NatMX4 his3::P_{GPD}-$GAL4^{484}$-ER-HIS3 leu2/leu2::P_{GAL1}-clb1-mDK-LEU2 spo13Δ::HIS3MX6::spo13-mD::HphMX4*
4A	Z35034	*MPC70/MPC70-eGFP::KanMX4 CNM67-tdTomato::NatMX4 his3::P_{GPD}-$GAL4^{484}$-ER-HIS3 leu2/leu2::P_{GAL1}-clb1-mDK-LEU2*
4B	Z33848	*SPC72-eGFP::KanMX4 CNM67-tdTomato::NatMX4 his3::P_{GPD}-$GAL4^{484}$-ER-HIS3*
4B	Z33847	*SPC72-eGFP::KanMX4 CNM67-tdTomato::NatMX4 his3::P_{GPD}-$GAL4^{484}$-ER-HIS3 spo13Δ::HIS3MX6::spo13-mD::HphMX4*
4B	Z35135	*SPC72-eGFP::KanMX4 CNM67-tdTomato::NatMX4 his3::P_{GPD}-$GAL4^{484}$-ER-HIS3 spo13Δ::HIS3MX6::spo13-mD-m2::HphMX4*

4B	Z35031	*MPC70/MPC70-eGFP::KanMX4 CNM67-tdTomato::NatMX4 his3::P_{GPD}-$GAL4^{484}$-ER-HIS3*
4B	Z35032	*MPC70/MPC70-eGFP::KanMX4 CNM67-tdTomato::NatMX4 his3::P_{GPD}-$GAL4^{484}$-ER-HIS3 spo13Δ::HIS3MX6::spo13-mD::HphMX4*
4B	Z35216	*MPC70/MPC70-eGFP::KanMX4 CNM67-tdTomato::NatMX4 his3::P_{GPD}-$GAL4^{484}$-ER-HIS3 spo13Δ::HIS3MX6::spo13-mD-m2::HphMX4*
4C	Z33845	*SPC72-eGFP::KanMX4 CNM67-tdTomato::NatMX4 his3::P_{GPD}-$GAL4^{484}$-ER-HIS3 leu2/leu2::P_{GAL1}-clb1-mDK-LEU2*
4C	Z33846	*SPC72-eGFP::KanMX4 CNM67-tdTomato::NatMX4 his3::P_{GPD}-$GAL4^{484}$-ER-HIS3 leu2/leu2::P_{GAL1}-clb1-mDK-LEU2 spo13Δ::HIS3MX6::spo13-mD::HphMX4*
4C	Z35136	*SPC72-eGFP::KanMX4 CNM67-tdTomato::NatMX4 his3::P_{GPD}-$GAL4^{484}$-ER-HIS3 leu2/leu2::P_{GAL1}-clb1-mDK-LEU2 spo13Δ::HIS3MX6::spo13-mD-m2::HphMX4*
4C	Z35034	*MPC70/MPC70-eGFP::KanMX4 CNM67-tdTomato::NatMX4 his3::P_{GPD}-$GAL4^{484}$-ER-HIS3 leu2/leu2::P_{GAL1}-clb1-mDK-LEU2*
4C	Z35033	*MPC70/MPC70-eGFP::KanMX4 CNM67-tdTomato::NatMX4 his3::P_{GPD}-$GAL4^{484}$-ER-HIS3 leu2/leu2::P_{GAL1}-clb1-mDK-LEU2 spo13Δ::HIS3MX6::spo13-mD::HphMX4*
4C	Z35217	*MPC70/MPC70-eGFP::KanMX4 CNM67-tdTomato::NatMX4 his3::P_{GPD}-$GAL4^{484}$-ER-HIS3 leu2/leu2::P_{GAL1}-clb1-mDK-LEU2 spo13Δ::HIS3MX6::spo13-mD-m2::HphMX4*
5A	Z35509	*SPC72-eGFP::KanMX4 CNM67-tdTomato::NatMX4 cdc20::P_{HSL1}-CDC20-HphMX4 ama1Δ::CaURA3 spo13Δ::HIS3MX6*
5A	Z35620	*SPC72-eGFP::KanMX4 CNM67-tdTomato::NatMX4 cdc20::P_{HSL1}-CDC20-HphMX4 ama1Δ::CaURA3 spo13Δ::HIS3MX6 cdc5-as::HphMX4*
5A	Z35662	*SPC72-eGFP::KanMX4 CNM67-tdTomato::NatMX4 cdc20::P_{HSL1}-CDC20-HphMX4 ama1Δ::CaURA3 spo13Δ::BleMX4 ime2::KanMX4::ime2-as-LEU2*
5B	Z38261	*cdc20::P_{HSL1}-CDC20-HphMX4 ama1Δ::CaURA3 CDC5-ha3::URA3 spo13::Ha3-SPO13-NatMX4*
5C	Z12185	Wild-type
5C	Z17454	*CDC5-ha3::URA3*
5C	Z6619	*CDC5-ha3::URA3 spo13Δ::HIS3MX6*
5D	Z37002	*cdc20::P_{HSL1}-CDC20-HphMX4 cdc5::P_{SCC1}-CDC5-KanMX4 his3::P_{GPD}-$GAL4^{484}$-ER-HIS3 ura3::P_{GAL1}-PBD-ha3-URA3*
5D	Z37001	*cdc20::P_{HSL1}-CDC20-HphMX4 cdc5::P_{SCC1}-CDC5-KanMX4 his3::P_{GPD}-$GAL4^{484}$-ER-HIS3 ura3::P_{GAL1}-PBD-FAA-ha3-URA3*
5E	Z35197	*SPC72-eGFP::KanMX4 CNM67-tdTomato::NatMX4 cdc20::P_{HSL1}-CDC20-HphMX4 ama1Δ::CaURA3*
5E	Z35509	*SPC72-eGFP::KanMX4 CNM67-tdTomato::NatMX4 cdc20::P_{HSL1}-CDC20-HphMX4 ama1Δ::CaURA3 spo13Δ::HIS3MX6*
5E	Z35369	*SPC72-eGFP::KanMX4 CNM67-tdTomato::NatMX4 cdc20::P_{HSL1}-CDC20-HphMX4 ama1Δ::CaURA3 cdc5-as::HphMX4*
6A,C	Z35833	*ndt80Δ::NatMX4 SPC72-eGFP::KlTRP1 CNM67-tdTomato::NatMX4 his3::P_{GPD}-$GAL4^{484}$-ER-HIS3*
6A,C	Z38067	*ndt80Δ::NatMX4 SPC72-eGFP::KlTRP1 CNM67-tdTomato::NatMX4 his3::P_{GPD}-$GAL4^{484}$-ER-HIS3 leu2::P_{GAL1}-IME2-ΔC-ha3-LEU2*
6A,C	Z38066	*ndt80Δ::NatMX4 SPC72-eGFP::KlTRP1 CNM67-tdTomato::NatMX4 his3::P_{GPD}-$GAL4^{484}$-ER-HIS3 ura3::P_{GAL1}-CDC5-ha3-URA3*
6B,D	Z35835	*ndt80Δ::NatMX4 SPC72-eGFP::KlTRP1 CNM67-tdTomato::NatMX4 his3::P_{GPD}-$GAL4^{484}$-ER-HIS3 leu2::P_{GAL1}-IME2-ΔC-ha3-LEU2 ura3::P_{GAL1}-CDC5-ha3-URA3*

6B,D	Z38441	ndt80Δ::NatMX4 SPC72-eGFP::KlTRP1 CNM67-tdTomato::NatMX4 his3::P_{GPD}-$GAL4^{484}$-ER-HIS3 leu2::P_{GAL1}-IME2-ΔC-ha3-LEU2 ura3::P_{GAL1}-CDC5-ha3-URA3 spo13Δ::HIS3MX6::spo13-10A::HphMX4
6B,D	Z38278	ndt80Δ::NatMX4 SPC72-eGFP::KlTRP1 CNM67-tdTomato::NatMX4 his3::P_{GPD}-$GAL4^{484}$-ER-HIS3 leu2::P_{GAL1}-IME2-ΔC-ha3-LEU2 ura3::P_{GAL1}-CDC5-ha3-URA3 spo13Δ::HIS3MX6::spo13-10D::HphMX4
7A	Z36179	cdc20-3 SPC72-eGFP::KlTRP1 CNM67-tdTomato::NatMX4 his3::P_{GPD}-$GAL4^{484}$-ER-HIS3
7A	Z36076	cdc20-3 SPC72-eGFP::KlTRP1 CNM67-tdTomato::NatMX4 his3::P_{GPD}-$GAL4^{484}$-ER-HIS3 leu2::P_{GAL1}-IME2-ΔC-ha3-LEU2
7A	Z36358	cdc20-3 SPC72-eGFP::KlTRP1 CNM67-tdTomato::NatMX4 his3::P_{GPD}-$GAL4^{484}$-ER-HIS3 leu2::P_{GAL1}-IME2-ΔC-ha3-LEU2 ura3::P_{GAL1}-SPO13-URA3
7B	Z36140	cdc20-3 SPC72-eGFP::KlTRP1 CNM67-tdTomato::NatMX4 his3::P_{GPD}-$GAL4^{484}$-ER-HIS3 ura3-P_{GAL1}-CDC5-ha3-URA3
7B	Z36418	cdc20-3 SPC72-eGFP::KlTRP1 CNM67-tdTomato::NatMX4 his3::P_{GPD}-$GAL4^{484}$-ER-HIS3 trp1-P_{GAL1}-CDC5-TRP1 leu2::P_{GAL1}-IME2-ΔC-ha3-LEU2
7B	Z36419	cdc20-3 SPC72-eGFP::KlTRP1 CNM67-tdTomato::NatMX4 his3::P_{GPD}-$GAL4^{484}$-ER-HIS3 trp1-P_{GAL1}-CDC5-TRP1 leu2::P_{GAL1}-IME2-ΔC-ha3-LEU2 ura3::P_{GAL1}-SPO13-URA3
7C	Z38279	cdc20-3 MPC70/MPC70-eGFP::KanMX4 CNM67-tdTomato::NatMX4 his3::P_{GPD}-$GAL4^{484}$-ER-HIS3 leu2::P_{GAL1}-IME2-ΔC-ha3-LEU2 ura3::P_{GAL1}-CDC5-ha3-URA3
7C	Z38417	cdc20-3 MPC70/MPC70-eGFP::KanMX4 CNM67-tdTomato::NatMX4 his3::P_{GPD}-$GAL4^{484}$-ER-HIS3 trp1::P_{GAL1}-NDT80-TRP1
7C	Z38280	cdc20-3 MPC70/MPC70-eGFP::KanMX4 CNM67-tdTomato::NatMX4 his3::P_{GPD}-$GAL4^{484}$-ER-HIS3 trp1::P_{GAL1}-NDT80-TRP1 leu2::P_{GAL1}-IME2-ΔC-ha3-LEU2 ura3::P_{GAL1}-CDC5-ha3-URA3
8A	Z32534	cdc28-as2 MPC70/MPC70-eGFP::KanMX4 NDT80/NDT80-sfGFP::KanMX4 ura3::P_{HIS3}-mScarlet-I-TUB1-URA3 cdc20::P_{HSL1}-CDC20-HphMX4 ama1Δ::NatMX4
8B	Z35798	cdc28-as2 cdc20::P_{HSL1}-CDC20-HphMX4 ama1Δ::NatMX4
8C	Z32534	cdc28-as2 MPC70/MPC70-eGFP::KanMX4 NDT80/NDT80-sfGFP::KanMX4 ura3::P_{HIS3}-mScarlet-I-TUB1-URA3 cdc20::P_{HSL1}-CDC20-HphMX4 ama1Δ::NatMX4
8C	Z38193	cdc28-as2 MPC70/MPC70-eGFP::KanMX4 NDT80/NDT80-sfGFP::KanMX4 ura3::P_{HIS3}-mScarlet-I-TUB1-URA3 cdc20::P_{HSL1}-CDC20-HphMX4 ama1Δ::NatMX4 spo13Δ::BleMX4
8C	Z35946	cdc28-as2 MPC70/MPC70-eGFP::KanMX4 NDT80/NDT80-sfGFP::KanMX4 ura3::P_{HIS3}-mScarlet-I-TUB1-URA3 cdc20::P_{HSL1}-CDC20-HphMX4 ama1Δ::NatMX4 cdc5-as::HphMX4
8D	Z36338	MPC70/MPC70-eGFP::KanMX4 CNM67-tdTomato::NatMX4 cdc20::P_{HSL1}-CDC20-HphMX4 ama1Δ::CaURA3 spc72Δ::KanMX4 leu2::SPC72-LEU2
8D	Z36209	MPC70/MPC70-eGFP::KanMX4 CNM67-tdTomato::NatMX4 cdc20::P_{HSL1}-CDC20-HphMX4 ama1Δ::CaURA3 spc72Δ::KanMX4 leu2::spc72-7-LEU2
8D	Z38848	MPC70/MPC70-eGFP::KanMX4 CNM67-tdTomato::NatMX4 cdc20::P_{HSL1}-CDC20-HphMX4 ama1Δ::CaURA3 spc72Δ::KanMX4 leu2::spc72-7-LEU2 cdc5-as::HphMX4
9A	Z31260	cdc20::P_{CLB2}-CDC20-HphMX4 ura3::P_{CUP1}-CDC20-URA3 MPC70/MPC70-eGFP::KanMX4 CNM67-tdTomato::NatMX4 leu2/leu2::P_{URA3}-tetR-tdTomato-LEU2

9A	Z31259	cdc20::P_{CLB2}-CDC20-HphMX4 ura3::P_{CUP1}-CDC20-URA3 MPC70/MPC70-eGFP::KanMX4 CNM67-tdTomato::NatMX4 leu2/leu2::P_{URA3}-tetR-tdTomato-LEU2 cdc28-as1
9B	Z38565	cdc20::P_{HSL1}-CDC20-HphMX4 trp1::P_{CUP1}-cdc20-3-TRP1 ama1Δ::NatMX4 leu2::P_{DMC1}-cAMA1-LEU2 cdh1::P_{HSL1}-CDH1-BleMX4 MPC70/MPC70-eGFP::KanMX4 [pRS426-P_{TEF2}-mRFP-spo20$^{51\text{-}91}$]
9C	Z38604	cdc20::P_{HSL1}-CDC20-HphMX4 trp1::P_{CUP1}-cdc20-3-TRP1 ama1Δ::NatMX4 leu2::P_{DMC1}-cAMA1-LEU2 cdh1::P_{HSL1}-CDH1-BleMX4 MPC70/MPC70-eGFP::KanMX4 his3/his3::P_{HIS3}-eGFP-TUB1-HIS3 [pRS426-P_{TEF2}-mRFP-spo20$^{51\text{-}91}$]
9C	Z38606	cdc20::P_{HSL1}-CDC20-HphMX4 trp1::P_{CUP1}-cdc20-3-TRP1 ama1Δ::NatMX4 leu2::P_{DMC1}-cAMA1-LEU2 cdh1::P_{HSL1}-CDH1-BleMX4 MPC70/MPC70-eGFP::KanMX4 his3/his3::P_{HIS3}-eGFP-TUB1-HIS3 cdc28-as2 [pRS426-P_{TEF2}-mRFP-spo20$^{51\text{-}91}$]
EV1A	Z35411	his3::P_{HIS3}-eGFP-TUB1::HIS3 leu2::P_{URA3}-tetR-tdTomato::LEU2
EV1A	Z35414	his3::P_{HIS3}-eGFP-TUB1::HIS3 leu2::P_{URA3}-tetR-tdTomato::LEU2 cdc20::P_{HSL1}-CDC20-HphMX4 spo13Δ::HIS3MX6
EV1A	Z35415	his3::P_{HIS3}-eGFP-TUB1::HIS3 leu2::P_{URA3}-tetR-tdTomato::LEU2 cdc20::P_{HSL1}-CDC20-HphMX4 clb1Δ::KanMX4
EV1A	Z39534	his3::P_{HIS3}-eGFP-TUB1::HIS3 leu2::P_{URA3}-tetR-tdTomato::LEU2 ama1Δ::NatMX4
EV1A	Z39537	his3::P_{HIS3}-eGFP-TUB1::HIS3 leu2::P_{URA3}-tetR-tdTomato::LEU2 ama1Δ::NatMX4 cdc20::P_{HSL1}-CDC20-HphMX4 spo13Δ::HIS3MX6
EV1A	Z39536	his3::P_{HIS3}-eGFP-TUB1::HIS3 leu2::P_{URA3}-tetR-tdTomato::LEU2 ama1Δ::NatMX4 cdc20::P_{HSL1}-CDC20-HphMX4 clb1Δ::KanMX4
EV1B	Z31931	cdc20::P_{SCC1}-CDC20-HphMX4 ama1Δ::CaURA3 cdh1::P_{HSL1}-CDH1-HphMX4 SPC72-eGFP::KanMX4 CNM67-tdTomato::NatMX4
EV1B	Z31930	cdc20::P_{SCC1}-CDC20-HphMX4 ama1Δ::CaURA3 cdh1::P_{HSL1}-CDH1-HphMX4 SPC72-eGFP::KanMX4 CNM67-tdTomato::NatMX4 spo13Δ::BleMX4
EV1B	Z32205	cdc20::P_{SCC1}-CDC20-HphMX4 ama1Δ::CaURA3 cdh1::P_{HSL1}-CDH1-BleMX4 MPC70/MPC70-eGFP::KanMX4 CNM67-tdTomato::NatMX4
EV1B	Z32206	cdc20::P_{SCC1}-CDC20-HphMX4 ama1Δ::CaURA3 cdh1::P_{HSL1}-CDH1-BleMX4 MPC70/MPC70-eGFP::KanMX4 CNM67-tdTomato::NatMX4 spo13Δ::HIS3MX6
EV1C	Z26184	SPC72-eGFP::KanMX4 CNM67-tdTomato::NatMX4
EV1C	Z31931	SPC72-eGFP::KanMX4 CNM67-tdTomato::NatMX4 cdc20::P_{SCC1}-CDC20-HphMX4 ama1Δ::CaURA3 cdh1::P_{HSL1}-CDH1-HphMX4
EV1C	Z31930	SPC72-eGFP::KanMX4 CNM67-tdTomato::NatMX4 cdc20::P_{SCC1}-CDC20-HphMX4 ama1Δ::CaURA3 cdh1::P_{HSL1}-CDH1-HphMX4 spo13Δ::BleMX4
EV2	Z33848	SPC72-eGFP::KanMX4 CNM67-tdTomato::NatMX4 his3::P_{GPD}-GAL4^{484}-ER-HIS3
EV2	Z33847	SPC72-eGFP::KanMX4 CNM67-tdTomato::NatMX4 his3::P_{GPD}-GAL4^{484}-ER-HIS3 spo13Δ::HIS3MX6::spo13-mD::HphMX4
EV2	Z33845	SPC72-eGFP::KanMX4 CNM67-tdTomato::NatMX4 his3::P_{GPD}-GAL4^{484}-ER-HIS3 leu2/leu2::P_{GAL1}-clb1-mDK-LEU2
EV2	Z33846	SPC72-eGFP::KanMX4 CNM67-tdTomato::NatMX4 his3::P_{GPD}-GAL4^{484}-ER-HIS3 leu2/leu2::P_{GAL1}-clb1-mDK-LEU2 spo13Δ::HIS3MX6::spo13-mD::HphMX4
EV3A	Z35835	ndt80Δ::NatMX4 SPC72-eGFP::KlTRP1 CNM67-tdTomato::NatMX4 his3::P_{GPD}-GAL4^{484}-ER-HIS3 leu2::P_{GAL1}-IME2-ΔC-ha3-LEU2 ura3::P_{GAL1}-CDC5-ha3-URA3
EV3A	Z35491	ndt80Δ::NatMX4 SPC72-eGFP::KlTRP1 CNM67-tdTomato::NatMX4 his3::P_{GPD}-GAL4^{484}-ER-HIS3 leu2::P_{GAL1}-IME2-ΔC-ha3-LEU2 ura3::P_{GAL1}-CDC5-ha3-URA3 spo13Δ::BleMX4

EV3B	Z38331	cdc20::P_{HSL1}-CDC20-URA3 ama1Δ::NatMX4
EV3B	Z38332	cdc20::P_{HSL1}-CDC20-URA3 ama1Δ::NatMX4 cdc28-as2
EV3B	Z38333	cdc20::P_{HSL1}-CDC20-URA3 ama1Δ::NatMX4 spo13Δ::HIS3MX6::spo13-10A::HphMX4
EV3B	Z38335	cdc20::P_{HSL1}-CDC20-URA3 ama1Δ::NatMX4 spo13Δ::HIS3MX6::spo13-10A::HphMX4 cdc28-as2
EV3B	Z38334	cdc20::P_{HSL1}-CDC20-URA3 ama1Δ::NatMX4 spo13Δ::HIS3MX6::spo13-10D::HphMX4
EV3B	Z38336	cdc20::P_{HSL1}-CDC20-URA3 ama1Δ::NatMX4 spo13Δ::HIS3MX6::spo13-10D::HphMX4 cdc28-as2
EV3C	Z33437	cdc20::P_{HSL1}-CDC20-HphMX4 ama1Δ::CaURA3 SPC72-eGFP::KlTRP1 CNM67-tdTomato::NatMX4
EV3C	Z38465	cdc20::P_{HSL1}-CDC20-HphMX4 ama1Δ::CaURA3 SPC72-eGFP::KlTRP1 CNM67-tdTomato::NatMX4 spo13Δ::HIS3MX6::spo13-10A::HphMX4
EV3C	Z38763	cdc20::P_{HSL1}-CDC20-HphMX4 ama1Δ::CaURA3 SPC72-eGFP::KlTRP1 CNM67-tdTomato::NatMX4 spo13Δ::HIS3MX6::spo13-10D::HphMX4
EV4A	Z36471	cdc20-3 SPC72-eGFP::KlTRP1 CNM67-tdTomato::NatMX4 his3::P_{GPD}-GAL4^{484}-ER-HIS3 cdc5-as::HphMX4
EV4A	Z36472	cdc20-3 SPC72-eGFP::KlTRP1 CNM67-tdTomato::NatMX4 his3::P_{GPD}-GAL4^{484}-ER-HIS3 cdc5-as::HphMX4 leu2::P_{GAL1}-IME2-ΔC-ha3-LEU2
EV4A	Z36076	cdc20-3 SPC72-eGFP::KlTRP1 CNM67-tdTomato::NatMX4 his3::P_{GPD}-GAL4^{484}-ER-HIS3 leu2::P_{GAL1}-IME2-ΔC-ha3-LEU2
EV4B	Z38637	cdc20-3 MPC70/MPC70-eGFP::KanMX4 CNM67-tdTomato::NatMX4 his3/his3::P_{GPD}-GAL4^{484}-ER-HIS3 trp1::P_{GAL1}-NDT80-TRP1 leu2::P_{GAL1}-IME2-ΔC-ha3-LEU2 ura3::P_{GAL1}-CDC5-ha3-URA3
EV4B	Z38638	cdc20-3 MPC70/MPC70-eGFP::KanMX4 CNM67-tdTomato::NatMX4 his3/his3::P_{GPD}-GAL4^{484}-ER-HIS3 trp1::P_{GAL1}-NDT80-TRP1 leu2::P_{GAL1}-IME2-ΔC-ha3-LEU2 ura3::P_{GAL1}-CDC5-ha3-URA3 mpc54Δ::AurCMX4
EV5A	Z31670	SPC72-eGFP::KanMX4 ura3::P_{HIS3}-mScarlet-I-TUB1-URA3
EV5A	Z31671	SPC72-eGFP::KanMX4 ura3::P_{HIS3}-mScarlet-I-TUB1-URA3 cdc28-as1
EV5A	Z31400	MPC70/MPC70-eGFP::KanMX4 ura3::P_{HIS3}-mScarlet-I-TUB1-URA3
EV5A	Z31401	MPC70/MPC70-eGFP::KanMX4 ura3::P_{HIS3}-mScarlet-I-TUB1-URA3 cdc28-as1
EV5B	Z32038	MPC70/MPC70-eGFP::KanMX4
EV5B	Z32450	MPC70/MPC70-eGFP::KanMX4 cdc28-as1
EV5B	Z32037	MPC54/MPC54-eGFP::KanMX4
EV5B	Z32036	MPC54/MPC54-eGFP::KanMX4 cdc28-as1
EV5B	Z32038	MPC70/MPC70-eGFP::KanMX4
EV5B	Z32450	MPC70/MPC70-eGFP::KanMX4 cdc28-as1
EV5B	Z32035	SPO74/SPO74-sfGFP::KlTRP1
EV5B	Z32040	SPO74/SPO74-sfGFP::KlTRP1 cdc28-as1
S1	Z32536	MPC70/MPC70-eGFP::KanMX4 PDS1-mNeonGreen::KlTRP1 SPC72-mScarlet::KlTRP1
S1	Z32535	MPC70/MPC70-eGFP::KanMX4 PDS1-mNeonGreen::KlTRP1 SPC72-mScarlet::KlTRP1 spo13Δ::BleMX4
S2	Z37615	cdc20::P_{HSL1}-CDC20-HphMX4 ama1Δ::NatMX4 leu2::P_{DMC1}-cAMA-LEU2 cdh1::P_{HSL1}-CDH1-BleMX4 REC8-ha3::URA3 PDS1-AID*::KanMX4
S2	Z37616	cdc20::P_{HSL1}-CDC20-HphMX4 ama1Δ::NatMX4 leu2::P_{DMC1}-cAMA-LEU2 cdh1::P_{HSL1}-CDH1-BleMX4 REC8-ha3::URA3 PDS1-AID*::KanMX4 ura3::P_{CUP1}-OsTIR1-myc3::URA3

S3	Z33437	SPC72-eGFP::KlTRP1 CNM67-tdTomato::NatMX4 cdc20::P_{HSL1}-CDC20-HphMX4 ama1Δ::CaURA3
S3	Z35320	SPC72-eGFP::KlTRP1 CNM67-tdTomato::NatMX4 cdc20::P_{HSL1}-CDC20-HphMX4 ama1Δ::CaURA3 clb3Δ::TRP1
S3	Z33291	SPC72-eGFP::KanMX4 CNM67-tdTomato::NatMX4 cdc20::P_{HSL1}-CDC20-HphMX4 ama1Δ::CaURA3 clb4Δ::KanMX4
S3	Z32908	MPC70/MPC70-eGFP::KanMX4 CNM67-tdTomato::NatMX4 cdc20::P_{HSL1}-CDC20-HphMX4 ama1Δ::CaURA3
S3	Z38575	MPC70/MPC70-eGFP::KanMX4 CNM67-tdTomato::NatMX4 cdc20::P_{HSL1}-CDC20-HphMX4 ama1Δ::CaURA3 clb3Δ::TRP1
S3	Z32988	MPC70/MPC70-eGFP::KanMX4 CNM67-tdTomato::NatMX4 cdc20::P_{HSL1}-CDC20-HphMX4 ama1Δ::CaURA3 clb4Δ::HphMX4
S4A	Z12185	Wild-type
S4A,B	Z35267	SPC72-eGFP::KlTRP1 CNM67-tdTomato::NatMX4 cdc20::P_{HSL1}-CDC20-HphMX4
S4A,B	Z35268	SPC72-eGFP::KlTRP1 CNM67-tdTomato::NatMX4 cdc20::P_{HSL1}-CDC20-HphMX4 spo13Δ::BleMX4
S4A,B	Z39487	SPC72-eGFP::KlTRP1 CNM67-tdTomato::NatMX4 cdc20::P_{HSL1}-CDC20-HphMX4 mcm21Δ::BleMX4
S4A,B	Z39485	SPC72-eGFP::KlTRP1 CNM67-tdTomato::NatMX4 cdc20::P_{HSL1}-CDC20-HphMX4 iml3Δ::CaURA3MX4
S5A	Z32337	SPC72-eGFP::KanMX4 CNM67-tdTomato::NatMX4 cdc5:P_{SCC1}-CDC5-KanMX4
S5A	Z32336	SPC72-eGFP::KanMX4 CNM67-tdTomato::NatMX4 cdc5:P_{SCC1}-CDC5-KanMX4 spo13Δ::HIS3MX6
S5A	Z32335	SPC72-eGFP::KanMX4 CNM67-tdTomato::NatMX4 spo13Δ::HIS3MX6
S5B	Z35198	SPC72-eGFP::KanMX4 CNM67-tdTomato::NatMX4 cdc20::P_{HSL1}-CDC20-HphMX4 ama1Δ::CaURA3 cdc28-as1
S5B	Z35370	SPC72-eGFP::KanMX4 CNM67-tdTomato::NatMX4 cdc20::P_{HSL1}-CDC20-HphMX4 ama1Δ::CaURA3 cdc28-as1 cdc5-as::HphMX4
S5B	Z35200	SPC72-eGFP::KanMX4 CNM67-tdTomato::NatMX4 cdc20::P_{HSL1}-CDC20-HphMX4 ama1Δ::CaURA3 cdc28-as1 ime2::KanMX4::ime2-as-LEU2
S5C	Z33437	SPC72-eGFP::KlTRP1 CNM67-tdTomato::NatMX4 cdc20::P_{HSL1}-CDC20-HphMX4 ama1Δ::CaURA3
S5C	Z38204	SPC72-eGFP::KlTRP1 CNM67-tdTomato::NatMX4 cdc20::P_{HSL1}-CDC20-HphMX4 ama1Δ::CaURA3 IME2-ΔC-ha3-HIS3MX6
S5C	Z39253	SPC72-eGFP::KlTRP1 CNM67-tdTomato::NatMX4 cdc20::P_{HSL1}-CDC20-HphMX4 ama1Δ::CaURA3 IME2-ΔC-ha3-HIS3MX6 cdc5-as::HphMX4
S6	Z24053	cdc20::P_{CLB2}-CDC20-KanMX6 trp1::P_{CUP1}-CDC20-TRP1 MPC70/MPC70-eGFP::KanMX4 CNM67-tdTomato::NatMX4 leu2/leu2::P_{URA3}-tetR-tdTomato-LEU2
S6	Z31053	cdc20::P_{CLB2}-CDC20-KanMX6 trp1::P_{CUP1}-CDC20-TRP1 MPC70/MPC70-eGFP::KanMX4 CNM67-tdTomato::NatMX4 leu2/leu2::P_{URA3}-tetR-tdTomato-LEU2 cdc5-as::HphMX4
S6	Z31260	cdc20::P_{CLB2}-CDC20-HphMX4 ura3::P_{CUP1}-CDC20-URA3 MPC70/MPC70-eGFP::KanMX4 CNM67-tdTomato::NatMX4 leu2/leu2::P_{URA3}-tetR-tdTomato-LEU2
S6	Z31398	cdc20::P_{CLB2}-CDC20-HphMX4 ura3::P_{CUP1}-CDC20-URA3 MPC70/MPC70-eGFP::KanMX4 CNM67-tdTomato::NatMX4 leu2/leu2::P_{URA3}-tetR-tdTomato-LEU2 ime2::KanMX4::ime2-as-LEU2

[1]Strains are listed for each figure from left to right and/or top to bottom. [2]All SK1 strains are diploid with the background *MATa/MATα ho::LYS2 lys2 ade2Δ::hisG trp1Δ::hisG leu2Δ::hisG his3Δ::hisG ura3*. Mutations are homozygous unless stated otherwise.
