# Supplementary material for: The Spo13/Meikin pathway confines the onset of gamete differentiation to meiosis II in yeast
Source: EMBO J. 2022 Jan 13;41(4):e109446. doi: 10.15252/embj.2021109446 (PMC8844990; doi:10.15252/embj.2021109446)
Supplement: Supplementary file 2 — Expanded View Figures PDF [file EMBJ-41-e109446-s005.pdf]

Expanded View Figures

Figure EV1. Analysis of Spo13 function in cells lacking APC/C activators.

A Deletion of *AMA1* blocks spore formation and reduces nuclear division (to < 5%) in P_{HSL1}-*CDC20* spo13Δ and P_{HSL1}-*CDC20* clb1Δ cells. Images were taken at 23 h in SPM. Nuclei are labeled with TetR-RFP. Scale bar, 2 μm.

B Imaging of SPBs (Cnm67-RFP) and Spc72-GFP or Mpc70-GFP in *SPO13* and *spo13Δ* cells lacking all three APC/C activators. Top, time-lapse series. Bottom, meiotic events were quantified in cells synchronized *in silico* to SPB separation at entry into metaphase I (t = 0). Graphs show overlays of *SPC72-GFP* and *MPC70-GFP* strains. Data are representative of three independent experiments.

C Immunoblot detection of proteins in whole-cell extracts from control cells and from *SPO13* and *spo13Δ* cells lacking all three APC/C activators. C, sample from proliferating cells. The asterisk marks a non-specific band.

Source data are available online for this figure.

 © 2022 The Authors

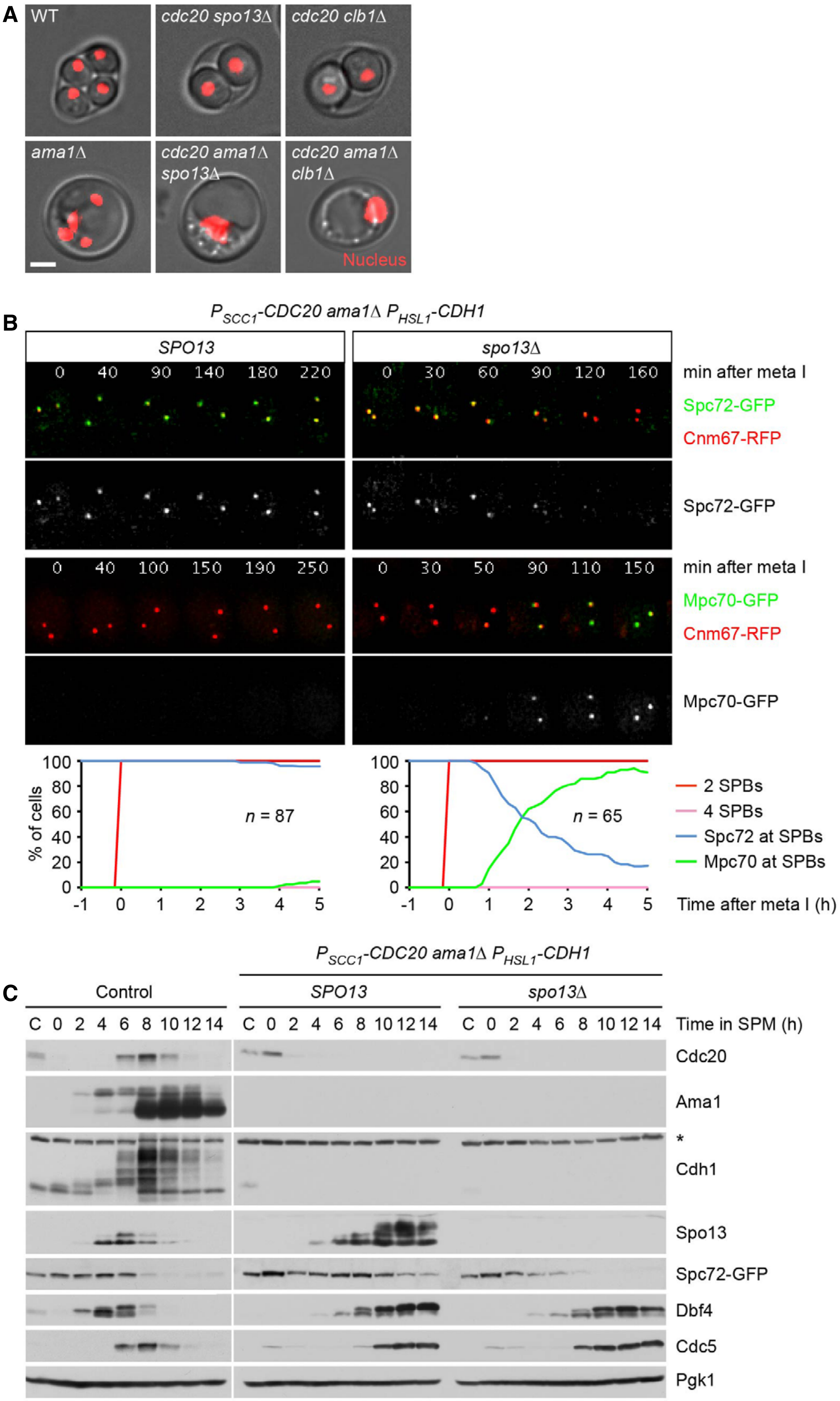
A
WT
cdc20 spo13Δ
cdc20 clb1Δ
ama1Δ
cdc20 ama1Δ spo13Δ
cdc20 ama1Δ clb1Δ
Nucleus
B
P_SCC1-CDC20 ama1Δ P_HSL1-CDH1
SPO13
spo13Δ
0 40 90 140 180 220
0 30 60 90 120 160
min after meta I
Spc72-GFP
Cnm67-RFP
Spc72-GFP
0 40 100 150 190 250
0 30 50 90 110 150
min after meta I
Mpc70-GFP
Cnm67-RFP
Mpc70-GFP
% of cells
n = 87
n = 65
2 SPBs
4 SPBs
Spc72 at SPBs
Mpc70 at SPBs
Time after meta I (h)
C
P_SCC1-CDC20 ama1Δ P_HSL1-CDH1
Control
SPO13
spo13Δ
C 0 2 4 6 8 10 12 14
Time in SPM (h)
Cdc20
Ama1
*
Cdh1
Spo13
Spc72-GFP
Dbf4
Cdc5
Pgk1

Figure EV1.

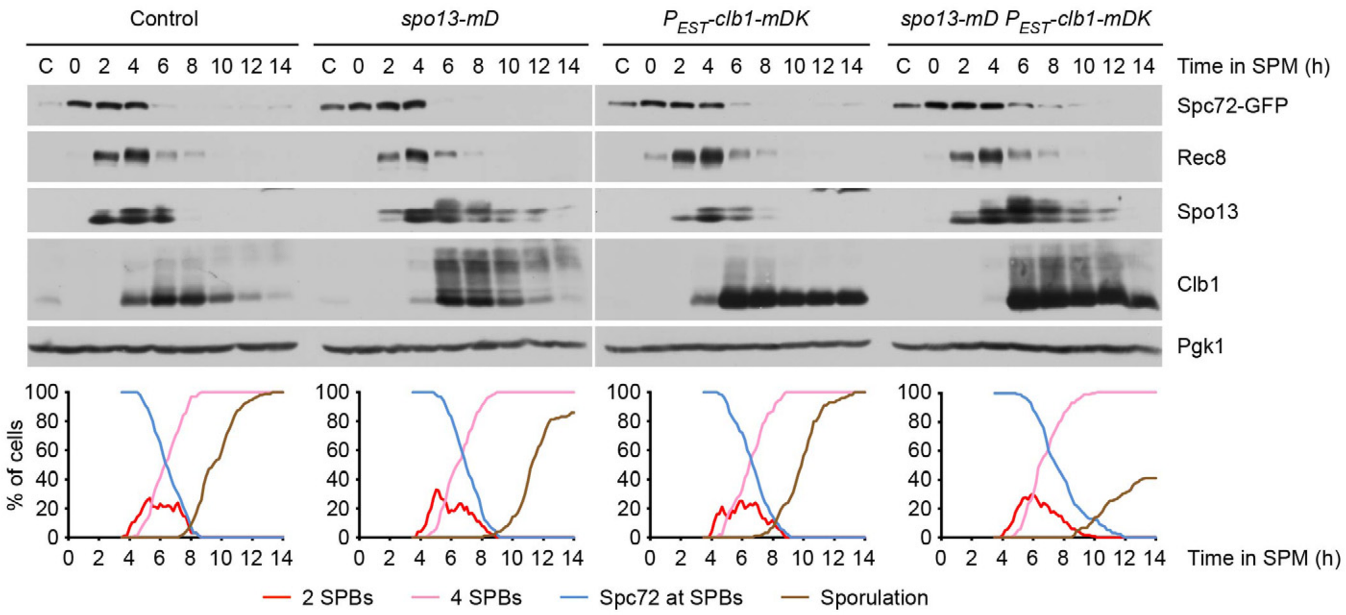

Figure EV2. Protein levels in strains expressing non-degradable Spo13 and/or Clb1.

Analysis of meiosis in cells containing Spo13-mD and/or expressing Clb1-mDK from the P_{EST} promoter. Estradiol was added at 4 h in SPM to induce P_{EST}-*clb1-mDK*. Top, immunoblot detection of proteins in whole-cell extracts. C, sample from proliferating cells. Bottom, culture aliquots were subjected to live-imaging of SPBs (Cnm67-RFP) and Spc72-GFP. Graphs show quantification of meiotic events (n = 100 cells per strain).

Source data are available online for this figure.

Figure EV3. Regulation of Spo13 by Cdk1 activity.

A Imaging of SPBs (Cnm67-RFP) and Spc72-GFP in *ndt80Δ* and *ndt80Δ spo13Δ* cells induced to express P_{EST}-*IME2*-ΔC plus P_{EST}-*CDC5* at 4 h in SPM (t = 0). Top, time-lapse series. Bottom, quantification of cells with Spc72 at SPBs. In *ndt80Δ* cells, the *SPO13* deletion causes only a small advance in Spc72 removal (19 min; 95% CI, 8–30; P = 0.001; Welch's t-test).

B Immunoblot detection of Spo13, Spo13-10A, and Spo13-10D in whole-cell extracts from P_{HSL1}-*CDC20* ama1Δ cells progressing into metaphase I. Cdc28-as2 was inhibited with 1Na-PP1 at 8 h in SPM (arrow). C, sample from proliferating cells.

C, D Imaging of SPBs (Cnm67-RFP) and Spc72-GFP in P_{HSL1}-*CDC20* ama1Δ cells containing different *SPO13* alleles. (C) Time-lapse series of *spo13-10A* and *spo13-10D* cells. (D) Spc72's presence at SPBs was quantified in *spo13-10A*, *spo13-10D*, *spo13Δ*, and control cells synchronized *in silico* to SPB separation at entry into metaphase I (t = 0). *spo13-10D* cells remove Spc72 with similar timing after entry into metaphase I as *spo13Δ* cells (P = 0.64, Welch's t-test).

Data information: Data are representative of two (A) or three (C, D) independent experiments.
Source data are available online for this figure.

 © 2022 The Authors

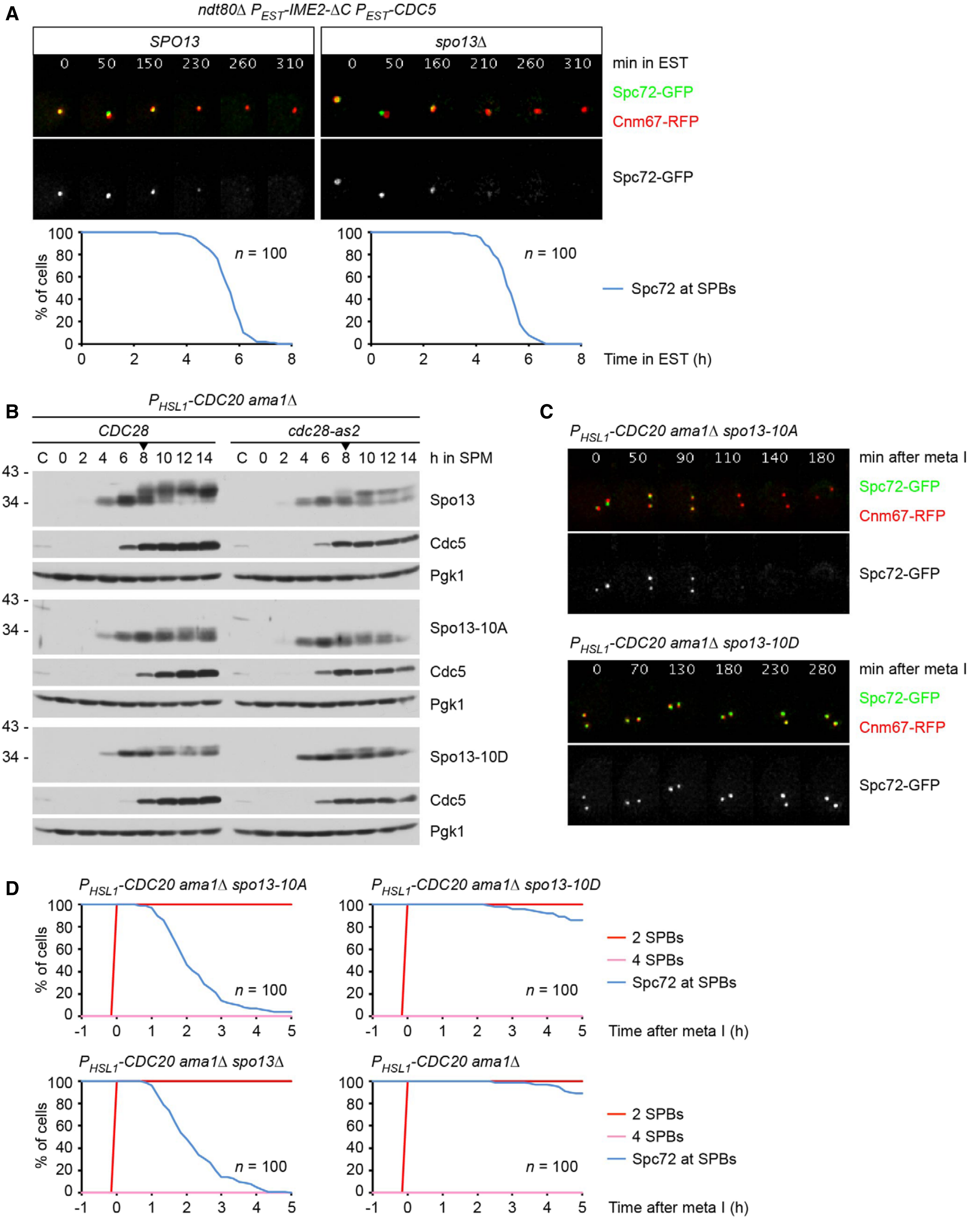
A
ndt80Δ P_{EST}-IME2-ΔC P_{EST}-CDC5
SPO13
spo13Δ
0 50 150 230 260 310
0 50 160 210 260 310
min in EST
Spc72-GFP
Cnm67-RFP
Spc72-GFP
% of cells
n = 100
Spc72 at SPBs
Time in EST (h)
B
P_{HSL1}-CDC20 ama1Δ
CDC28
cdc28-as2
C 0 2 4 6 8 10 12 14
h in SPM
43 -
34 -
Spo13
Cdc5
Pgk1
Spo13-10A
Spo13-10D
C
P_{HSL1}-CDC20 ama1Δ spo13-10A
0 50 90 110 140 180
min after meta I
P_{HSL1}-CDC20 ama1Δ spo13-10D
0 70 130 180 230 280
D
P_{HSL1}-CDC20 ama1Δ spo13-10A
P_{HSL1}-CDC20 ama1Δ spo13-10D
P_{HSL1}-CDC20 ama1Δ spo13Δ
P_{HSL1}-CDC20 ama1Δ
2 SPBs
4 SPBs
Spc72 at SPBs
Time after meta I (h)

Figure EV3.

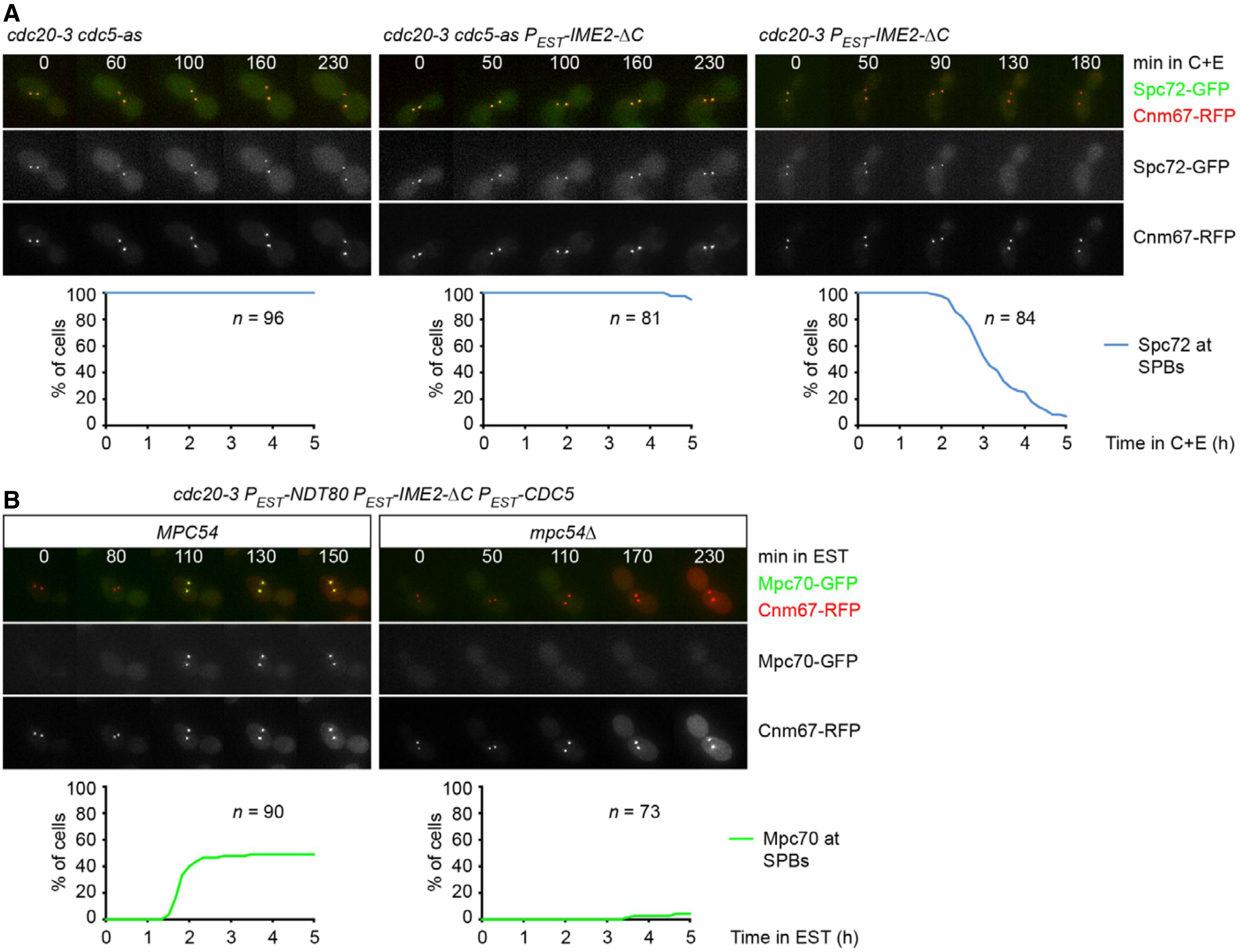

Figure EV4. Analysis of Spc72 removal and MP assembly in mitotic cells.

A Mitotic *cdc20-3* cells containing *cdc5-as* and/or P_{EST}-*IME2*-ΔC were shifted to 36°C for 80 min. At $t = 0$, cells were treated with CMK and estradiol (C + E) to inhibit Cdc5-as and induce P_{EST}-*IME2*-ΔC, respectively. Top, time-lapse series from the imaging of SPBs (Cnm67-RFP) and Spc72-GFP. Frame width, 19 μm. Bottom, quantification of cells with Spc72-GFP at SPBs.

B Mitotic *cdc20-3 MPC54* and *cdc20-3 mpc54Δ* cells containing P_{EST}-*NDT80*, P_{EST}-*IME2*-ΔC, and P_{EST}-*CDC5* were shifted to 36°C for 30 min and treated with estradiol ($t = 0$). Top, time-lapse series from the imaging of SPBs (Cnm67-RFP) and Mpc70-GFP. Frame width, 19 μm. Bottom, quantification of cells with Mpc70-GFP at SPBs.

Data information: Data are representative of two (A) or three (B) independent experiments.

 © 2022 The Authors

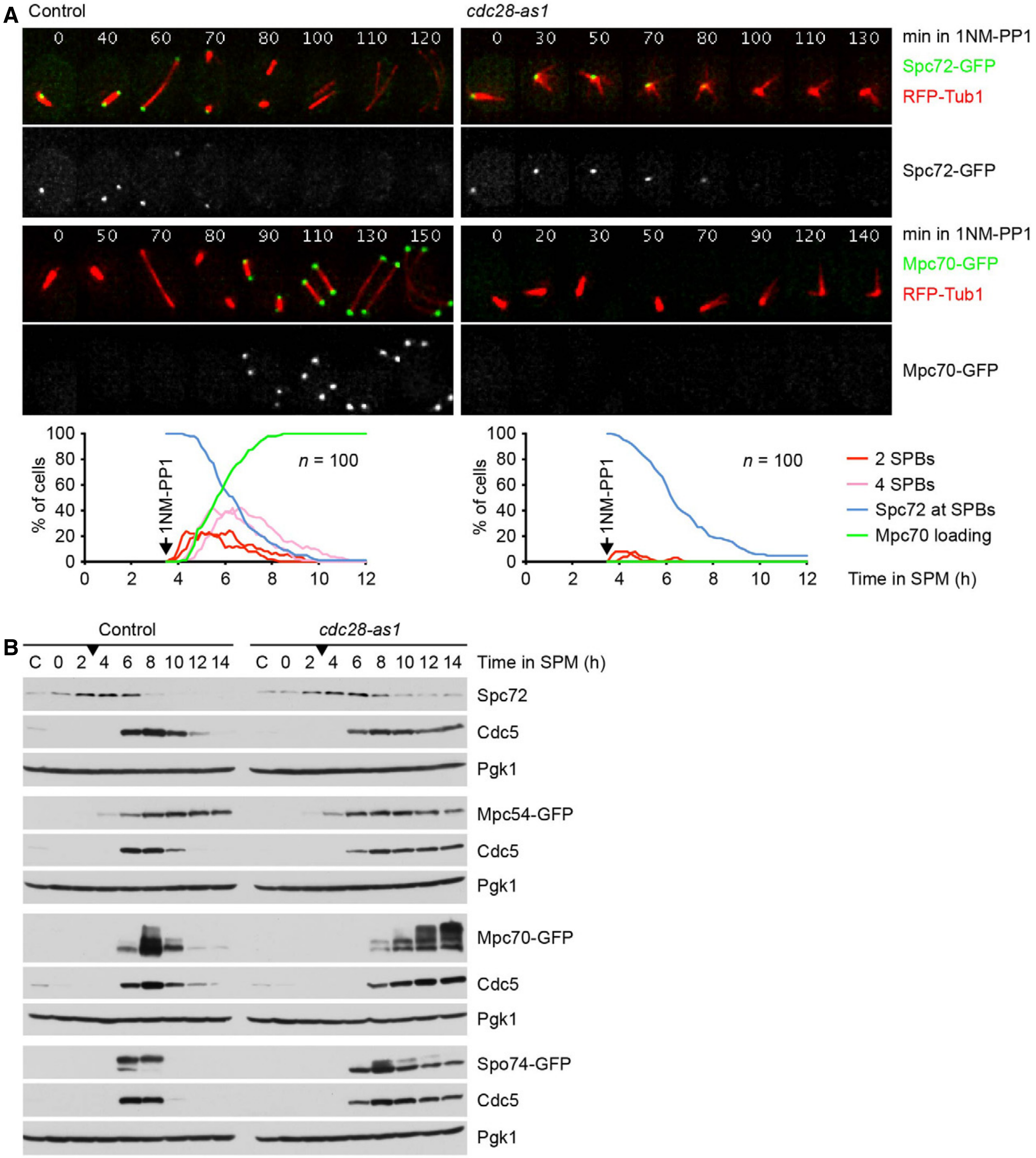

Figure EV5. Analysis of Spc72 and MP proteins after inhibition of Cdk1.

A Imaging of microtubules (RFP-Tub1) and Spc72-GFP or Mpc70-GFP in control and *cdc28-as1* cells treated with 1NM-PP1 at 3.5 h in SPM. Top, time-lapse series. Bottom, quantification of meiotic events. Graphs show overlays of *SPC72-GFP* and *MPC70-GFP* strains.

B Immunoblot detection of Spc72 and GFP-tagged MP proteins in whole-cell extracts from control and *cdc28-as1* strains treated with 1NM-PP1 at 3 h in SPM (arrow). The appearance of Cdc5 marks the activation of Ndt80. C, sample from proliferating cells.

Source data are available online for this figure.

© 2022 The Authors
