# Supplementary figures and images for: The Spo13/Meikin pathway confines the onset of gamete differentiation to meiosis II in yeast
Source: EMBO J. 2022 Jan 13;41(4):e109446. doi: 10.15252/embj.2021109446 (PMC8844990; doi:10.15252/embj.2021109446)

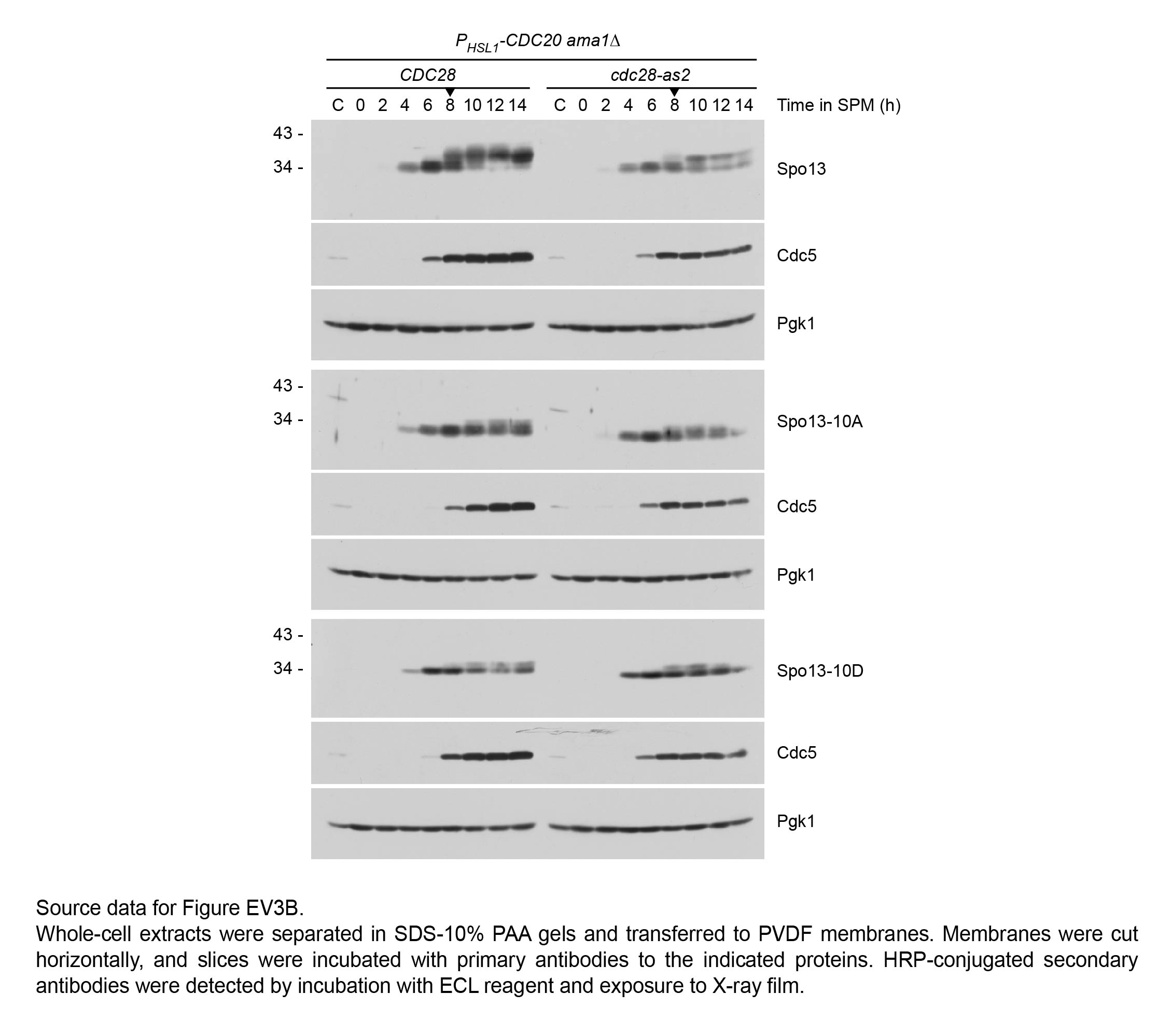

Supplement: Supplementary file 3 — Source Data for Expanded View/Appendix [file EMBJ-41-e109446-s003.zip › Source Data for Expanded View and Appendix/EMBOJ-2021-109446_SourceDataForFigureEV3B.jpg]

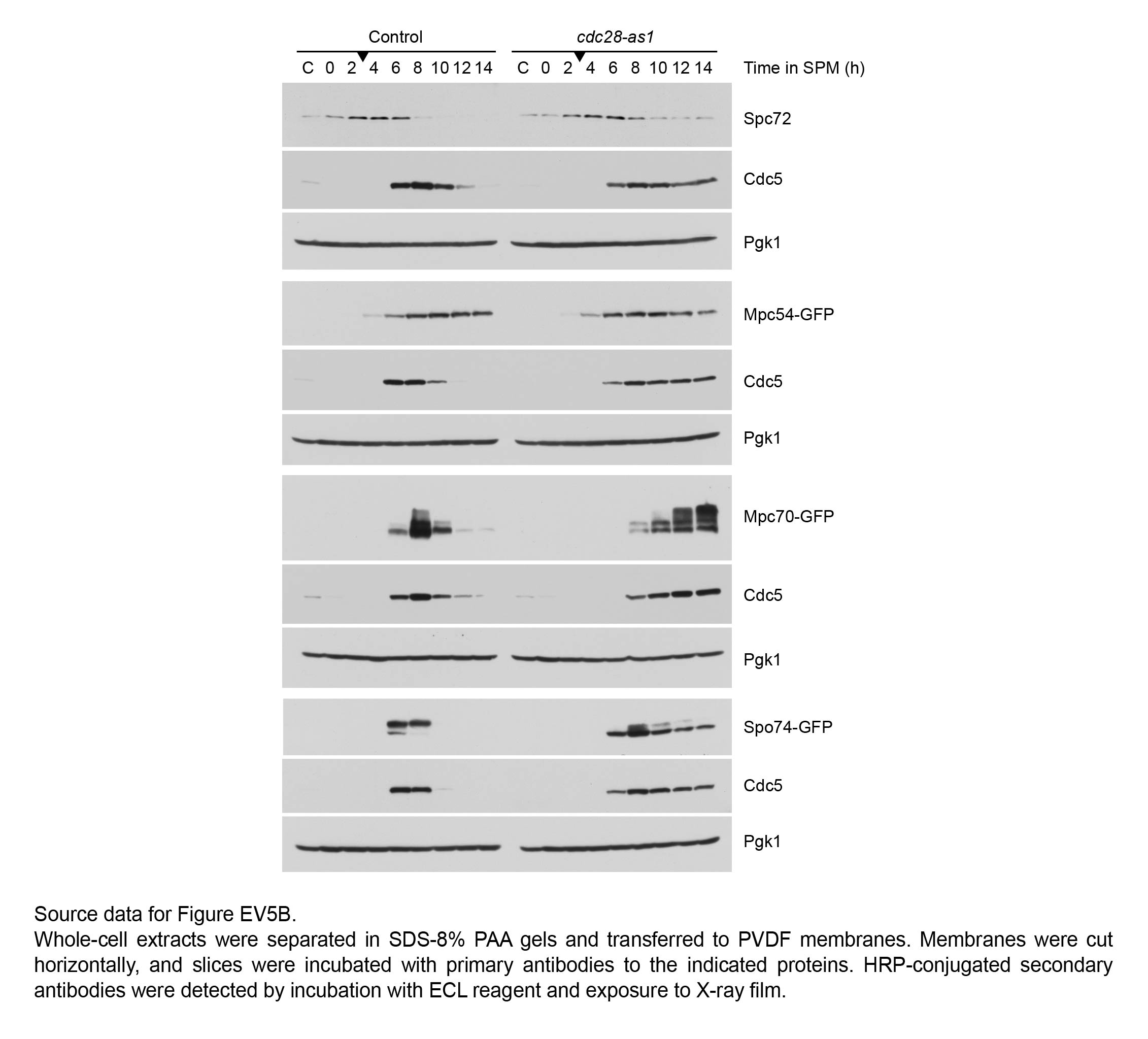

Supplement: Supplementary file 3 — Source Data for Expanded View/Appendix [file EMBJ-41-e109446-s003.zip › Source Data for Expanded View and Appendix/EMBOJ-2021-109446_SourceDataForFigureEV5B.jpg]

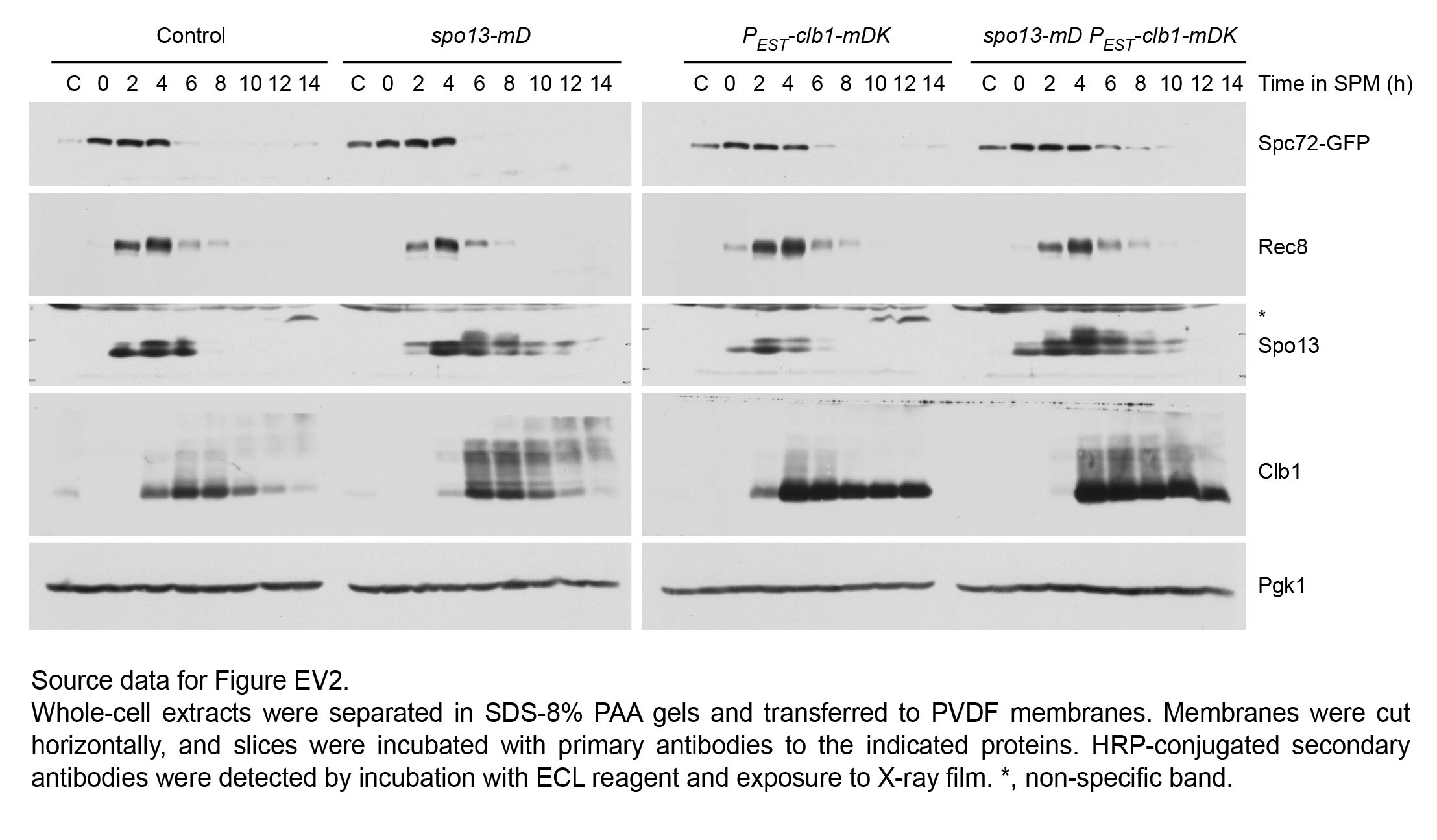

Supplement: Supplementary file 3 — Source Data for Expanded View/Appendix [file EMBJ-41-e109446-s003.zip › Source Data for Expanded View and Appendix/EMBOJ-2021-109446_SourceDataForFigureEV2.jpg]

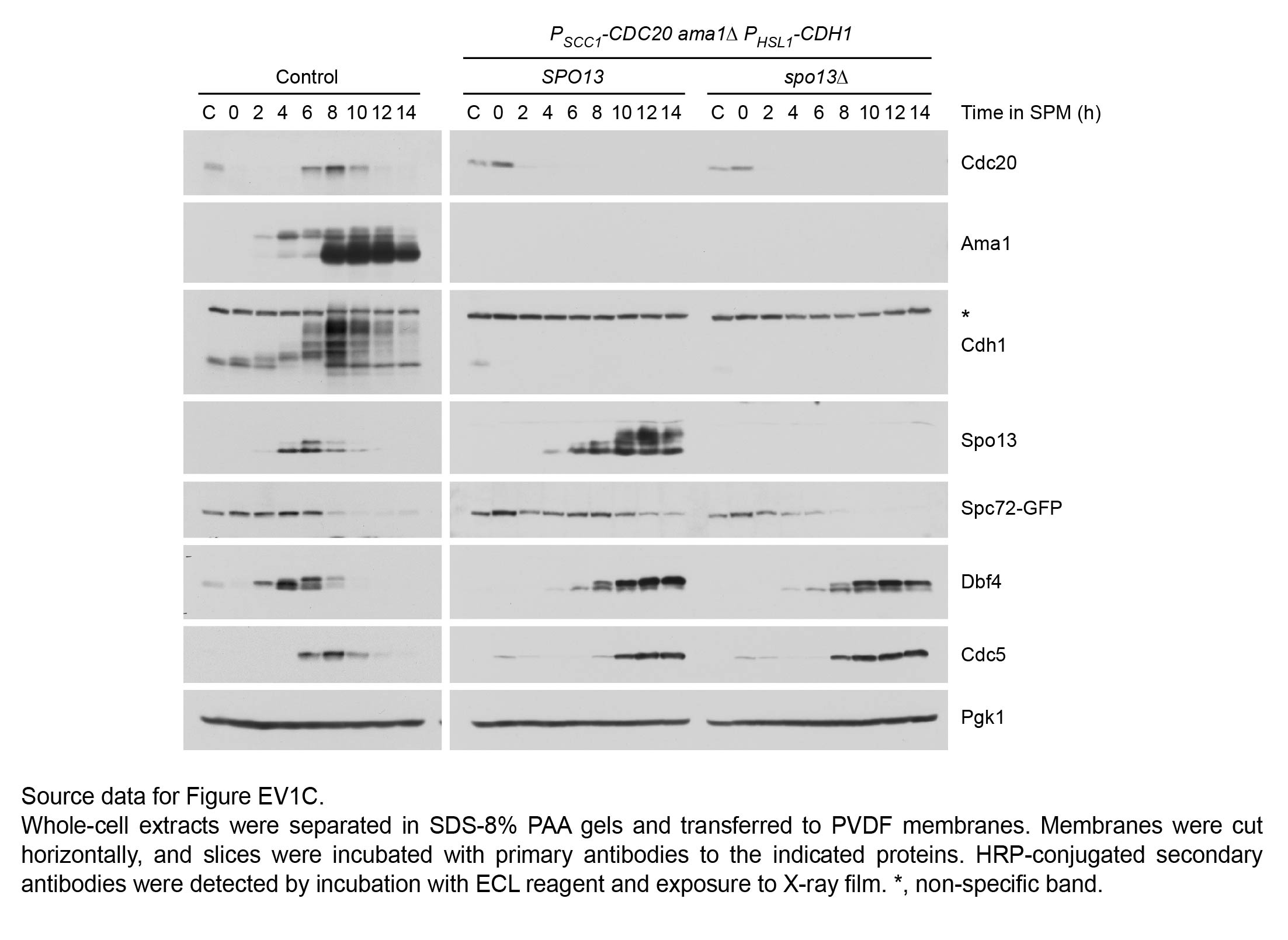

Supplement: Supplementary file 3 — Source Data for Expanded View/Appendix [file EMBJ-41-e109446-s003.zip › Source Data for Expanded View and Appendix/EMBOJ-2021-109446_SourceDataForFigureEV1C.jpg]

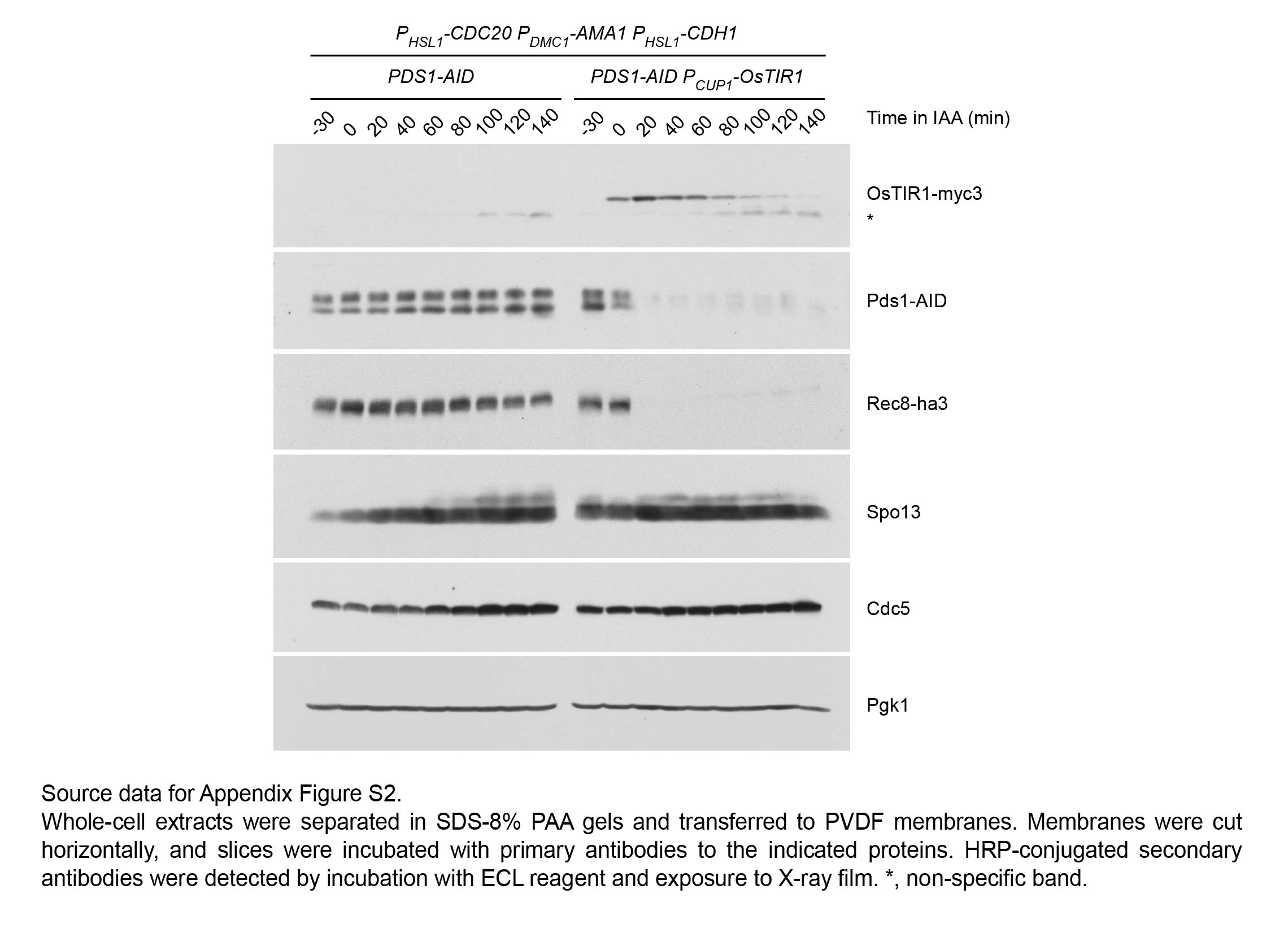

Supplement: Supplementary file 3 — Source Data for Expanded View/Appendix [file EMBJ-41-e109446-s003.zip › Source Data for Expanded View and Appendix/EMBOJ-2021-109446_SourceDataForFigureS2.jpg]

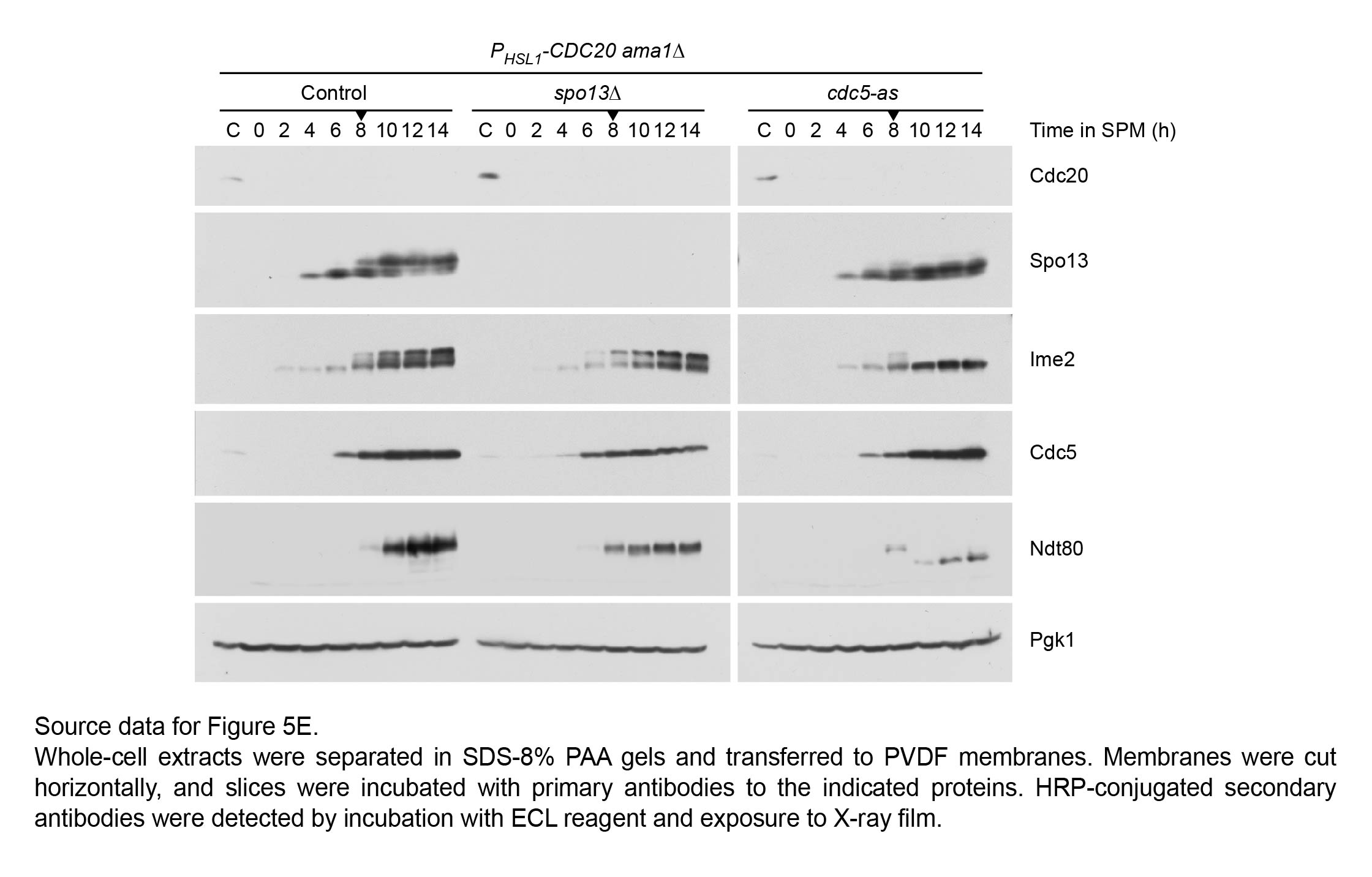

Supplement: Supplementary file 4 — Source Data for Figure 5 [file EMBJ-41-e109446-s006.zip › EMBOJ-2021-109446R-Source_data_for_Fig_5E-sd.jpg]

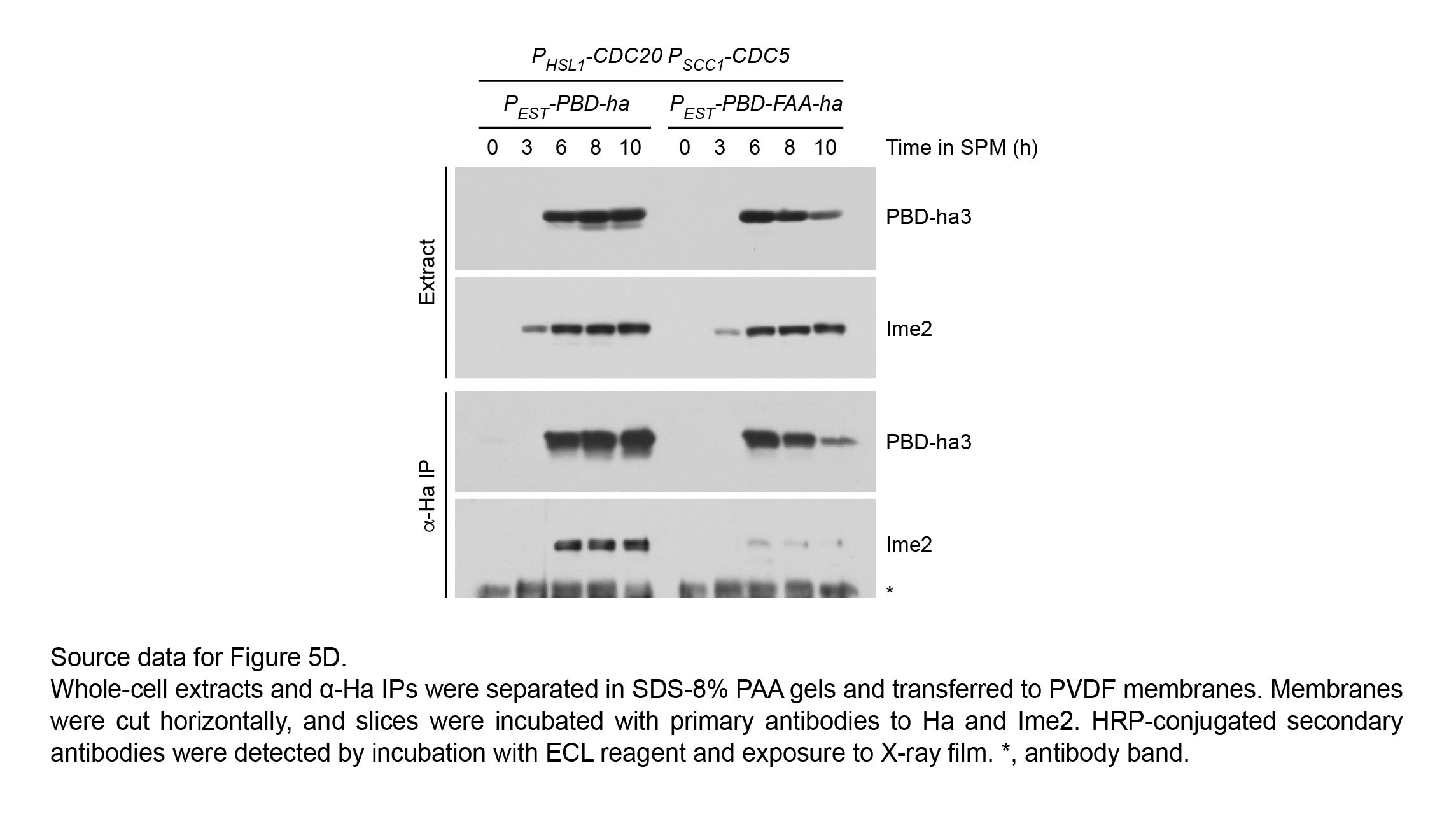

Supplement: Supplementary file 4 — Source Data for Figure 5 [file EMBJ-41-e109446-s006.zip › EMBOJ-2021-109446R-Source_data_for_Fig_5D-sd.jpg]

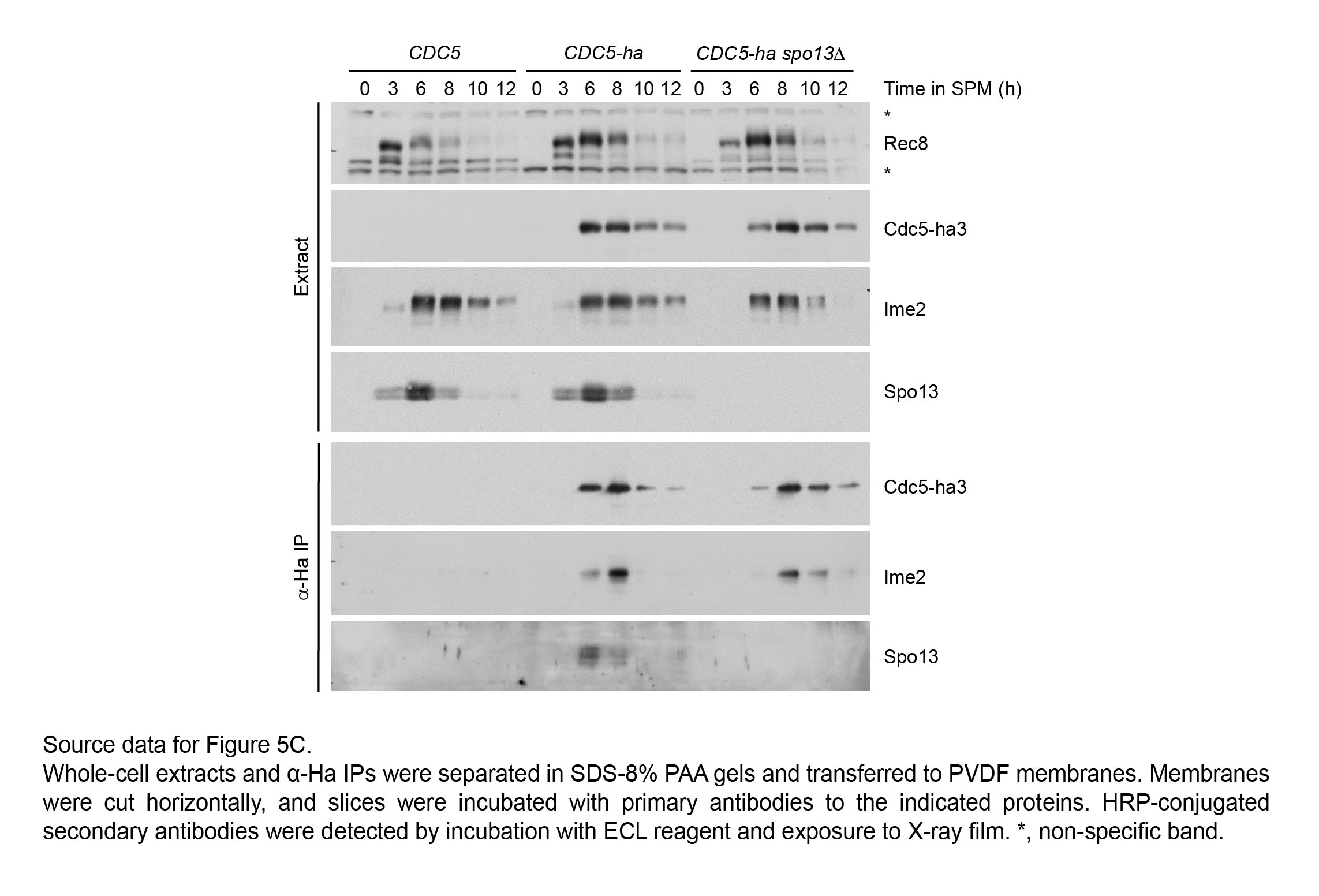

Supplement: Supplementary file 4 — Source Data for Figure 5 [file EMBJ-41-e109446-s006.zip › EMBOJ-2021-109446R-Source_data_for_Fig_5C-sd.jpg]

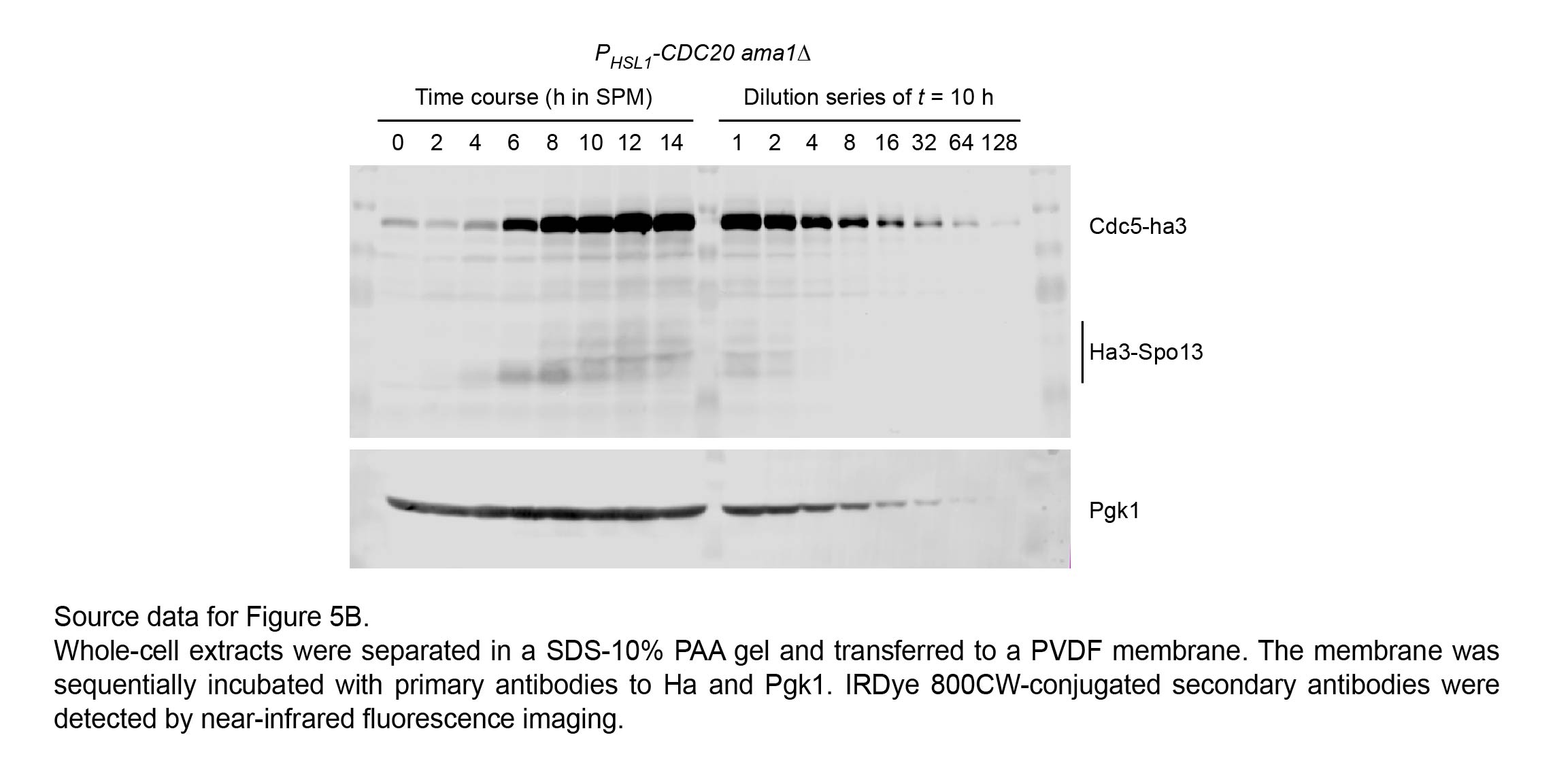

Supplement: Supplementary file 4 — Source Data for Figure 5 [file EMBJ-41-e109446-s006.zip › EMBOJ-2021-109446R-Source_data_for_Fig_5B-sd.jpg]

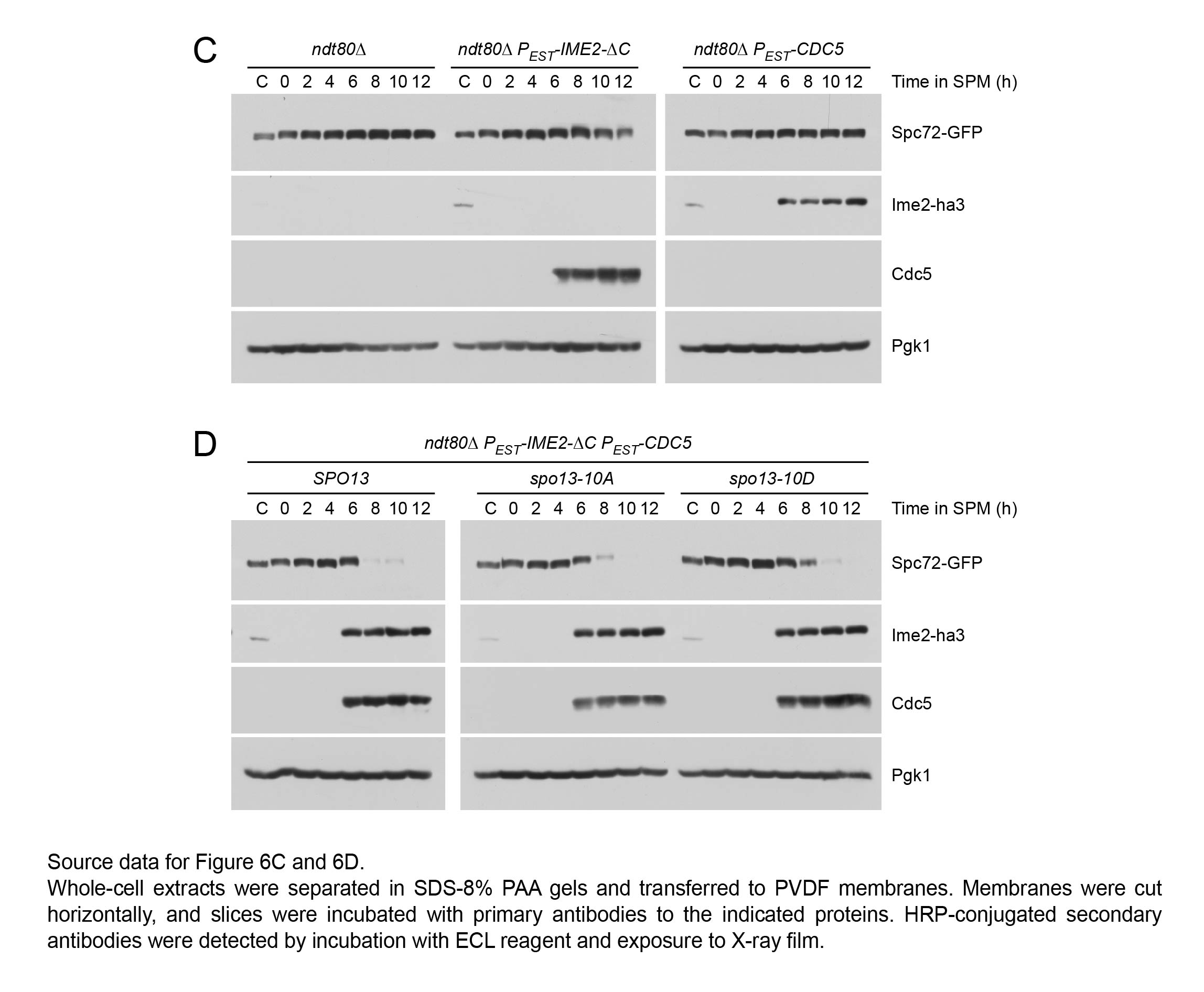

Supplement: Supplementary file 5 — Source Data for Figure 6 [file EMBJ-41-e109446-s001.jpg]
