# Supplementary material for: The Spo13/Meikin pathway confines the onset of gamete differentiation to meiosis II in yeast
Source: EMBO J. 2022 Jan 13;41(4):e109446. doi: 10.15252/embj.2021109446 (PMC8844990; doi:10.15252/embj.2021109446)
Supplement: Supplementary file 6 [file EMBJ-41-e109446-s002.pdf]

THE EMBO JOURNAL

The Spo13/Meikin Pathway Confines the Onset of Gamete Differentiation to Meiosis II in Yeast

Tugce Oz, Valentina Mengoli, Julie Rojas, Katarzyna Jonak, Marianne Braun, Ievgeniia Zagoriy, and Wolfgang Zachariae
DOI: 10.15252/embj.2021109446

Corresponding author(s): Wolfgang Zachariae (zachar@biochem.mpg.de)

Review Timeline:

Submission Date:	12th Aug 21
Editorial Decision:	24th Sep 21
Revision Received:	6th Dec 21
Accepted:	20th Dec 21

Editor: Hartmut Vodermaier

Transaction Report:

(Note: With the exception of the correction of typographical or spelling errors that could be a source of ambiguity, letters and reports are not edited. Depending on transfer agreements, referee reports obtained elsewhere may or may not be included in this compilation. Referee reports are anonymous unless the Referee chooses to sign their reports.)

1st Editorial Decision **24th Sep 2021**

Thank you for submitting your study on Spo13/Meikin regulation of yeast gamete differentiation for our consideration. We have now heard back from all three referees that had agreed to review it. Based on their unanimously positive comments, we shall be happy to pursue this work further for publication in The EMBO Journal, pending satisfactory addressing of a number of specific issues listed in the reports copied below.

REFEREE REPORTS

Referee #1:

This manuscript by from Oz et al describes work in budding yeast meiosis aimed at understanding how the initiation of spore formation is coupled to progression through the meiotic cell division phases. As always with studies from the Zachariae lab, this work is a prime example of the immense power of yeast genetics coupled to beautiful cell biology. The experiments are impeccable, the approaches are elegant and extraordinarily rigorous, and the conclusions are very solid. Especially the experiments where certain aspects of the regulation of meiotic plaque formation are recapitulated in mitosis are very impressive. This work provides important new insight into a fascinating biological question. Thus, I strongly support publication of this manuscript, and have only a few (minor) questions/remarks.

1) In the introduction: "These include, for instance, the reduplication of spindle pole bodies (SPBs, the yeast centrosomes), the initiation of spore differentiation, translation of the Clb3 cyclin, redistribution of mitochondria, and deprotection of centromeric cohesin". I believe in the cited papers, Carlile and Amon, 2008 should be included (when discussing Clb3 translation)
2) In the experiments in mitotic cells, the authors nicely show that Mpc70 can be accumulated at SPBs. As I understand it Mpc70 recruitment is one of the early steps in spore formation and pro-spore membrane establishment. Do the authors have any idea whether more downstream events also initiate under these conditions? Would there be reasons why this does not work in mitotic cells?
3) In figure 1D, can the plotted data from spo13del cells (now in App Fig 1) be included for reference? It would help with the interpretation. The actual move could stay in App Figs, but the plots would be helpful here.
4) In figure 5D, a negative control (i.e. a non HA tag condition) is missing. I realize the PBD-FAA mutant serves a binding mutant but also having a no tag-control would help in gauging the effect of this mutant.
5) Throughout the paper I kept asking myself how Spo13 would control this effect. Of course, the authors have done a commendable job in tackling this question and the explanation that is put forward in the discussion is very tantalizing, but I am wondering if the way these observations are currently discussed throughout the MS cannot be made a bit clearer. Could clarifying or concluding statements be made more readily when showing/discussing spo13 observations? The striking effect combined with the way we think of spo13 right now can leave the reader feeling a bit 'left in the dark' at moments. Also regarding spo13/meikin: can the authors comment on and include a reference to the recent work by the Cheeseman and Lampson labs? Do authors also detect separase-dependent cleavage? Could this be an explanation for the Spo13/Cdc5 role the authors propose?
6) Related to the spo13 point: is there a way to investigate if kinetochore localization of spo13 is needed for this role? Could the mif2/CENP-C binding site be mutated? Would one then expect this system to still work (for example in the mitotic system)?

Gerben Vader

Referee #2:

The manuscript „The Spo13/Meikin Pathway Confines the Onset of Gamete Differentiation to Meiosis II in Yeast" by Oz et al. reports coordination of meiotic progression with gamete formation. In budding yeast, initiation of gamete formation is coupled to the formation of meiotic plaques at spindle pole bodies. To turn the spindle pole body from a microtubule organizing center into a membrane attachment point, the gamma-tubulin receptor Spc72 has to be removed from the spindle pole body. The pathway, which initiates the formation of meiotic plaques in a timely manner at the onset of meiosis II remained enigmatic so far. The main finding of the manuscript is that the kinases Cdc5 and Ime2 are essential for initiating gamete formation and that Spo13/Meikin has to be degraded by the anaphase-promoting complex to allow meiotic plaque formation. This event happens upon reactivation of Cdk1 at entry into metaphase meiosis II. The organization of these specific events might be a blueprint for the activation of other events that need to be restricted to meiosis II.
The manuscript is of excellent quality and only one major point needs to be addressed before publication. Previous publications by the Knop lab (Gordon et al. EMBOJ 2006) and the Taxis lab (Renicke et al. Genetics 2017) have described the involvement of Spc72 in meiotic plaque formation and have described Spc72 localization during meiosis in detail, respectively. Both publications and their findings were not discussed by the authors. The authors describe in the abstract the spindle pole bodies cannot form meiotic plaques during meiosis I because of the presence of Spc72 at the spindle pole bodies during this stage and they credit Spc72 with an inhibitory role in their final model (Figure 9C). Yet, in Renicke et al it was published that Spc72 is not always present a both spindle pole bodies during meiosis I and that a minority of cells do not show any Spc72 signals during meiosis I. Moreover, downregulation of Spc72 early in meiosis did not show any abnormalities in sporulation. How does this fit to the model of the authors?

Referee #3:

Gamete formation requires successful meiosis followed by and gamete differentiation. Here the authors characterize the regulation of spore differentiation in S. cerevisiae, from metaphase I to metaphase II. They conclude that 1) Spc72 blocks formation of the meiotic plaque (MP), 2) PLK/Cdc5 and Ime2 kinases remove Spc72 from SPBs, 3) Spo13-Cdc5 inhibits both Ime2 and formation of the MP directly, 4) Clb-Cdk1 promotes activity of Spo13-Cdc5 and formation of the MP, 5) Cdc5 kinase activity promotes removal of Spc72 via phosphorylation of Ime2, and 6) APC/CCdc20 mediates both the degradation of Spo13-Cdc5 and also the inactivation of Cdk1-Clb1.
These findings are significant in their novelty. The link between meiosis II and gamete differentiation has previously been studied only in Drosophila, where no relationship was found. Despite contrasting with the Drosophila study, the claims are overall convincing. The mechanism of regulation proposed could be of interest to not only those interested in cell division, but in any type of cell cycle regulation.
Outlined below is one weakness in the manuscript: 1) the assumption that Cdc5 kinase functions upstream of Ime2.
Major criticisms:
1. Page 8, paragraph 2, "Live-imaging revealed that spo13Δ mutants remove Spc72 from and recruit Mpc70 to SPBs with wild-type kinetics." If Spo13, as the focus of the paper, is responsible for persistence of Spc72 and inhibition of MP formation, why is Spc72 removal and Mpc70 recruitment to SPBs unaffected in spo13Δ mutants, as shown in Appendix Fig S1? Perhaps Spo13 inhibition is generally short-lived in the cell?
2. Page 8, paragraph 3, Please provide rationale behind the use of the ama1Δ in the strains used in many of the experiments. The ama1Δ mutant does not appear to have been tested on its own, which would determine if deletion of AMA1 is sufficient to inhibit removal of Spc72.
3. Page 11, paragraph 3, "inhibition of Cdc5 activity prevents the removal of Spc72 from SPBs." Page 12, paragraph 2, "inhibition of Ime2 activity prevents the removal of Spc72." Page 13, paragraph 1, "Which expression of either Ime2-ΔC or Cdc5 alone has no effect, co-expression of both kinases causes degradation of removal from SPBs of Spc72." Page 22, paragraph 3, "We have placed Ime2 at the bottom of the signaling cascade from APC/C^{Cdc20} in the nucleus to Spc72 on the cytoplasmic face of the SPB because it is the only meiosis-specific regulator required for reconstituting Spc72 removal in mitotic cells." This seems like a weak reason to place Ime2 at the bottom of the signaling cascade. If there is additional justification to do so, please provide.
Minor criticisms:
1. Page 14, paragraph 2, "These conditions would limit MP assembly to metaphase II of meiosis but do not exist in a normal mitosis." Should "but" be "because they"? Which would change the sentence to: "These conditions would limit MP assembly to metaphase II of meiosis because they do not exist in a normal mitosis."
2. Page 16, paragraph 1, "While Cdk1-Clb1 plays a specific role in Spc72 removal, this does not seem to be the case in MP assembly." This sentence is confusing because it implies that Clb1 does not play a role in MP assembly. It appears instead that Clb1 plays a role in MP assembly but it can be replaced by Clb2, 3 or 4. The authors could clarify, by adding an additional sentence.
3. General note. There are places in the paper where the meaning becomes convoluted when discussing the absence of a protein or an effect (i.e. when using words such as "lack," "block," "deletion," "prevent," or "removal" back to back). For instance, page 11, paragraph 3, "Indeed, depletion of Cdc5 prevents Spc72's removal also in the spo13Δ mutant." It's possible that this can't be avoided, but it can result in unnecessary confusion.

Point-by-point response to reviewer's comments

We thank all reviewers for their insightful comments, which we feel have substantially improved the manuscript. Please find below our point-by-point response in blue text. Major changes in the manuscript are in red text.

Referee #1 (Gerben Vader):
This manuscript by from Oz et al describes work in budding yeast meiosis aimed at understanding how the initiation of spore formation is coupled to progression through the meiotic cell division phases. As always with studies from the Zachariae lab, this work is a prime example of the immense power of yeast genetics coupled to beautiful cell biology. The experiments are impeccable, the approaches are elegant and extraordinarily rigorous, and the conclusions are very solid. Especially the experiments where certain aspects of the regulation of meiotic plaque formation are recapitulated in mitosis are very impressive. This work provides important new insight into a fascinating biological question. Thus, I strongly support publication of this manuscript, and have only a few (minor) questions/remarks.
We thank the reviewer for his positive and supportive comments on our study.

1) In the introduction: "These include, for instance, the reduplication of spindle pole bodies (SPBs, the yeast centrosomes), the initiation of spore differentiation, translation of the Clb3 cyclin, redistribution of mitochondria, and deprotection of centromeric cohesin". I believe in the cited papers, Carlile and Amon, 2008 should be included (when discussing Clb3 translation).
We have added the citation as requested by the reviewer (page 4) but note that the intention was to provide examples of meiosis II-specific processes with one key reference each. The Carlile and Amon (2008) paper was cited already when we discuss the role of translational control in meiosis (page 20).

2) In the experiments in mitotic cells, the authors nicely show that Mpc70 can be accumulated at SPBs. As I understand it Mpc70 recruitment is one of the early steps in spore formation and pro-spore membrane establishment. Do the authors have any idea whether more downstream events also initiate under these conditions? Would there be reasons why this does not work in mitotic cells?
Expression of Ndt80 and Ime2 causes MP assembly in mitotic cells as shown by the accumulation of Mpc70 at SPBs (**Fig 7C**), which depends on another MP protein (Mpc54, **Fig EV4B**). Our **new Fig 9C** reveals one reason why these mitotic cells cannot sporulate. To elicit MP formation, the following conditions have to be met: first, removal of Spc72 by expression of Ime2 in the presence of Cdc5 (**Fig 7A, B**). Second, expression of Ndt80 to induce synthesis of MP proteins and establishment of a high-Cdk1 state to allow MP assembly. We achieved the latter by arresting *cdc20-3* ts-mutants at metaphase. However, **new Fig 9C** shows that PSM formation requires APC/C-Cdc20 activity. Sporulation requires additional processes not reconstituted by Ndt80 expression. For instance, PSM closure requires Ama1, whose expression depends on the meiosis-specific splicing factor Mer1 (Cooper et al., 2000).

3) In figure 1D, can the plotted data from spo13del cells (now in App Fig 1) be included for reference? It would help with the interpretation. The actual move could stay in App Figs, but the plots would be helpful here.
We prefer to keep the current arrangement of the data. First, **Fig 1** is already quite large; new data could only be added by removing others. We feel that the data currently shown in **Fig 1** are crucial for the logical flow of our story. Second, **Fig 1** analyzes the consequences of removing or inactivating Spo13 in Cdc20-depleted cells. **Fig 1D** fits into this scheme; it shows Spc72 removal and Mpc70 loading in *cdc20 spo13* and *cdc20 clb1Δ* double mutants. Third, the analysis of the *spo13Δ* single mutant in **Appendix Fig S1** uses different makers (Spc72-RFP, Mpc70-GFP, and Pds1-GFP) than the experiments in **Fig 1** (Cnm67-RFP plus Spc72-GFP or Mpc70-GFP). Thus, we are reluctant to separate the graphs from the corresponding images and show the *spo13Δ* single mutant in a separate figure.

4) In figure 5D, a negative control (i.e. a non-HA tag condition) is missing. I realize the PBD-FAA mutant serves a binding mutant but also having a no tag-control would help in gauging the effect of this mutant.
The PBD-FAA mutant is an established negative control for the binding of the PBD to a protein of interest (Song et al., 2000; Elia et al., 2003). We see a large difference between PBD and PBD-FAA with regard to Ime2-binding (**Fig 5D**), suggesting that Cdc5's association with Ime2 (**Fig 5C**) involves the PBD. It could be argued that in IP experiments, a tagged but biologically inactive bait is a better negative control than the untagged bait. In the former case, the beads carry antibody plus (inactive) bait, while in the latter, they carry only the antibody. In most IP experiments, one just does not have the luxury of a well characterized, inactive bait. An untagged PBD would tell us more about the significance of the extremely weak signal from the PBD-FAA IP than about the relevance of the strong signal from the PBD IP. However, our conclusions rest on the difference between functional and non-functional PBD. We feel that the information gained from repeating this experiment with an untagged PBD does not justify the effort in constructing and characterizing strains expressing untagged PBD.

5) Throughout the paper I kept asking myself how Spo13 would control this effect. Of course, the authors have done a commendable job in tackling this question and the explanation that is put forward in the discussion is very tantalizing, but I am wondering if the way these observations are currently discussed throughout the MS cannot be

made a bit clearer. Could clarifying or concluding statements be made more readily when showing/discussing spo13 observations? The striking effect combined with the way we think of spo13 right now can leave the reader feeling a bit 'left in the dark' at moments.
We presume that "the way we think of Spo13 right now" refers to the fact that Spo13 is usually discussed in the context of the meiosis I-specific behavior of kinetochores and centromeres. However, mutants defective in monopolin and centromeric cohesin protection show a different phenotype than the *spo13Δ* mutant (e.g., Toth et al., 2000), indicating that Spo13 has functions beyond the kinetochore/centromere. We find that one of these functions is the regulation of spore formation. In fact, **new Appendix Fig S4A, B** show that Spo13's role in the control of spore formation does not require intact kinetochores. It is therefore not surprising that some of our results and conclusions appear somewhat unfamiliar. We have made an effort to improve the structure and logical flow of the text.
Our work describes a regulatory network capable of confining MP assembly to meiosis II. We appreciate, however, that most of the underlying biochemical mechanisms remain to be elucidated (page 24). Our work broadens the range of processes controlled by Spo13, which should facilitate unraveling these mechanisms.

Also regarding spo13/meikin: can the authors comment on and include a reference to the recent work by the Cheeseman and Lampson labs? Do authors also detect separase-dependent cleavage? Could this be an explanation for the Spo13/Cdc5 role the authors propose?
We agree and have included a discussion of the work of Cheesman and Lampson (page 23). Furthermore, we have directly tested whether Spo13 is cleaved by separase. Briefly, we have activated separase in metaphase I-arrested cells by auxin-inducible degradation of Pds1. While Rec8 is rapidly cleaved, Spo13 is not affected (page 8; **new Appendix Fig S2**), suggesting that Spo13 is not a separase substrate. We note that the idea of Cdc5-Spo13 as an active kinase is consistent with the data of Cheesman and Lampson on Plk1-meikin. Furthermore, meikin's cleavage by separase is compatible with our two-step model for the regulation of meiosis II-specific processes (see page 23).

6) Related to the spo13 point: is there a way to investigate if kinetochore localization of spo13 is needed for this role? Could the mif2/CENP-C binding site be mutated? Would one then expect this system to still work (for example in the mitotic system)?
So far, mammalian meikin and *S. pombe* Moa1, but not *S. cerevisiae* Spo13, have been shown to bind CENP-C (Kim et al., 2015). Since meikin, Moa1, and Spo13 are poorly conserved, and structural information is not available, constructing and characterizing a CENP-C-binding mutant of Spo13 represents a considerable amount of work, which goes beyond the scope of this manuscript.
Instead, we have tested whether Spo13's function in sporulation depends on the integrity of kinetochores (**new Appendix Fig S4A, B**). Deletion of subunits of the inner kinetochore Ctf19-complex, such as Mcm21 or Iml3, is compatible with proliferation but prevents kinetochore re-assembly at entry into meiosis I (Borek et al., 2021). Most kinetochore proteins (including Mif2) are reduced or absent at centromeres, leading to massive chromosome miss-segregation. Nevertheless, *cdc20 mcm21Δ* and *cdc20 iml3Δ* mutants differ from *cdc20 spo13Δ* cells in that they retain Spc72 at SPBs and do not sporulate. Thus, Spo13's function in sporulation does not require intact kinetochores.

Referee #2:
The manuscript „The Spo13/Meikin Pathway Confines the Onset of Gamete Differentiation to Meiosis II in Yeast" by Oz et al. reports coordination of meiotic progression with gamete formation. In budding yeast, initiation of gamete formation is coupled to the formation of meiotic plaques at spindle pole bodies. To turn the spindle pole body from a microtubule organizing center into a membrane attachment point, the gamma-tubulin receptor Spc72 has to be removed from the spindle pole body. The pathway, which initiates the formation of meiotic plaques in a timely manner at the onset of meiosis II remained enigmatic so far. The main finding of the manuscript is that the kinases Cdc5 and Ime2 are essential for initiating gamete formation and that Spo13/Meikin has to be degraded by the anaphase-promoting complex to allow meiotic plaque formation. This event happens upon reactivation of Cdk1 at entry into metaphase meiosis II. The organization of these specific events might be a blueprint for the activation of other events that need to be restricted to meiosis II.
The manuscript is of excellent quality and only one major point needs to be addressed before publication. Previous publications by the Knop lab (Gordon et al. EMBOJ 2006) and the Taxis lab (Renicke et al. Genetics 2017) have described the involvement of Spc72 in meiotic plaque formation and have described Spc72 localization during meiosis in detail, respectively. Both publications and their findings were not discussed by the authors. The authors describe in the abstract the spindle pole bodies cannot form meiotic plaques during meiosis I because of the presence of Spc72 at the spindle pole bodies during this stage and they credit Spc72 with an inhibitory role in their final model (Figure 9C). Yet, in Renicke et al it was published that Spc72 is not always present a both spindle pole bodies during meiosis I and that a minority of cells do not show any Spc72 signals during meiosis I. Moreover, downregulation of Spc72 early in meiosis did not show any abnormalities in sporulation. How does this fit to the model of the authors?
We thank the reviewer for his/her positive comments on our work.

We have cited the papers commonly used to cover the Spc72-related issues relevant to our story: Spc72's function as γ-TuC receptor required for the nucleation of astral microtubules (Knop and Schiebel, 1998; Soues and Adams, 1998), its binding to the SPB protein Nud1 (Gruneberg et al., 2000), and its degradation and removal from SPBs at entry into meiosis II (Knop and Strasser, 2000). Knop and Strasser (2000) noted that Spc72 is degraded and removed from SPBs before MP formation but did not test whether the former is a prerequisite for the latter.
We have now included the Gordon et al. (2006) and Renicke et al. (2017) papers as additional references for the Spc72-Nud1 interaction, localization of Spc72 to SPBs in early meiosis and meiosis I, and the fact that the relevance of Spc72's degradation for MP assembly had been unclear (pages 5, 7, 22, and 24).
Gordon et al. (2006) and Renicke et al. (2017) have analyzed the role of Nud1 and the MEN pathway, respectively, in SPB inheritance and spore number control. These processes allow yeast cells to produce fewer spores under starvation conditions. The surviving spores are specified by a mechanism related to the age of the SPBs. This raises interesting questions about how yeast distinguishes SPBs of different age and has implications for population genetics. However, we do not work under starvation conditions. On the contrary, we make every effort to provide optimal sporulation conditions to achieve the high synchrony crucial for our experiments. Furthermore, most of our experiments use mutants that undergo only one division or arrest at metaphase I. While interesting, SPB inheritance and spore number control under starvation are not of immediate relevance to our story.
To inactivate Spc72, we shifted *cdc20 ama1Δ* strains carrying the *spc72-7* ts-mutation (Knop and Strasser, 2000; a gift from Michael Knop) to 37°C as cells arrest at metaphase I. Live-imaging and TEM show that these cells form MPs, demonstrating that Spc72 prevents MP formation at metaphase I (**Fig 3A, B**). By contrast, Renicke et al. (2017) did not arrest cells and used a different approach to inactivate Spc72. Therefore, these experiments are conceptually very different. Renicke et al. (2017) see Spc72 on most (89%) but not all SPBs at meiosis I, while we see Spc72 on SPBs in >98% of cells at this stage. This seems a small difference, given different synchronization protocols, fluorophores, and imaging setups.
Mpc70 recruitment is slower in *cdc20 ama1Δ spc72-7* cells than in *cdc20 ama1Δ spo13Δ* cells (**Fig 3A vs Fig 3C**), suggesting that Spc72 is not the only means by which Spo13 inhibits MP formation at metaphase I. Indeed, **Fig 8C** shows that Cdc5-Spo13 inhibits both Spc72 removal and MP assembly at metaphase I. To test whether Cdc5-Spo13 inhibits MP assembly in *cdc20 ama1Δ spc72-7* cells, we have inhibited Cdc5 in these cells (**new Fig 8D**). Cdc5 inhibition accelerates Mpc70 loading, confirming that Cdc5-Spo13 hinders MP assembly at metaphase I, even when Spc72 is inactive. When Spc72 is inactivated in cells progressing through meiosis, as in Renicke et al. (2017), MP formation is still limited by Spo13's degradation at anaphase I, and the kinetics of sporulation is not altered.

Referee #3:
Gamete formation requires successful meiosis followed by and gamete differentiation. Here the authors characterize the regulation of spore differentiation in S. cerevisiae, from metaphase I to metaphase II. They conclude that 1) Spc72 blocks formation of the meiotic plaque (MP), 2) PLK/Cdc5 and Ime2 kinases remove Spc72 from SPBs, 3) Spo13-Cdc5 inhibits both Ime2 and formation of the MP directly, 4) Clb-Cdk1 promotes activity of Spo13-Cdc5 and formation of the MP, 5) Cdc5 kinase activity promotes removal of Spc72 via phosphorylation of Ime2, and 6) APC/CCdc20 mediates both the degradation of Spo13-Cdc5 and also the inactivation of Cdk1-Clb1. These findings are significant in their novelty. The link between meiosis II and gamete differentiation has previously been studied only in Drosophila, where no relationship was found. Despite contrasting with the Drosophila study, the claims are overall convincing. The mechanism of regulation proposed could be of interest to not only those interested in cell division, but in any type of cell cycle regulation.
We thank the reviewer for his/her insightful and supportive comments on our work.

Outlined below is one weakness in the manuscript: 1) the assumption that Cdc5 kinase functions upstream of Ime2.
Major criticisms:
1. Page 8, paragraph 2, "Live-imaging revealed that spo13Δ mutants remove Spc72 from and recruit Mpc70 to SPBs with wild-type kinetics." If Spo13, as the focus of the paper, is responsible for persistence of Spc72 and inhibition of MP formation, why is Spc72 removal and Mpc70 recruitment to SPBs unaffected in spo13Δ mutants, as shown in Appendix Fig S1? Perhaps Spo13 inhibition is generally short-lived in the cell?
The reviewer might have overlooked an important piece of information hidden within the second half of the sentence, namely that Spc72 is removed with wild-type kinetics (**Appendix Fig S1A**), *although Pds1 degradation is delayed and SPB duplication does not occur* (Shonn et al., 2002). Since this paragraph was unclear, we have revised it (page 8). Briefly, in control cells, Spc72 is removed (blue trace) and Mpc70 is loaded (green trace) *after* Pds1 is degraded (yellow trace) at anaphase I. In the *spo13Δ* mutant, Pds1 degradation is delayed due to SAC activity (Shonn et al., 2002), while Spc72 removal and Mpc70 loading occur slightly earlier than in control cells. As a result, they now occur *before* Pds1 degradation (i.e., at metaphase I). We now provide statistics in the figure legend and in **Appendix Fig S1B**. These data, and **Fig 1D**, show that the *SPO13* deletion uncouples Spc72 removal and MP formation from the activity of APC/C-Cdc20. In *spo13Δ* cells, Spc72's degradation and removal from SPBs is driven by the Ndt80-dependent accumulation/activation of Cdc5 and Ime2. Accordingly, the *SPO13* deletion causes Spc72 degradation/removal and MP formation even in cells lacking all three APC/C activators (**Fig EV1B, C**). To put Spc72

degradation in *cdc20 ama1Δ cdh1 spo13Δ* cells in perspective, we have added analysis of protein levels in control cells (**revised Fig EV1C**).

2. Page 8, paragraph 3, Please provide rationale behind the use of the ama1Δ in the strains used in many of the experiments. The ama1Δ mutant does not appear to have been tested on its own, which would determine if deletion of AMA1 is sufficient to inhibit removal of Spc72.
Rationale for deleting AMA1: **Fig 1D** shows that the *SPO13* deletion causes exchange of Spc72 for Mpc70 in Cdc20-depleted cells. However, the *SPO13* deletion also causes nuclear division in Cdc20-depleted cells (Katis et al., 2004). We find that this division is blocked by the deletion of *AMA1* (**new Fig EV1A**), while Spc72 removal and MP formation still occur (**Fig 2A**). Thus, exchange of Spc72 for Mpc70 requires the degradation/inactivation of Spo13 (to remove Spc72) and a high-Cdk1 state (to allow MP assembly) but not nuclear division or Ama1 activity. The finding that *cdc20 spo13Δ* cells undergo nuclear division in an Ama1-dependent manner might indicate that Spo13 has a role in in preventing Ama1 from promoting nuclear division when Cdc20 is inhibited/absent (discussed on page 21).
Is deletion of AMA1 sufficient to inhibit removal of Spc72? APC/C promotes Spc72's degradation and removal from SPBs only indirectly, through the degradation of Spo13. *spo13Δ* cells degrade/remove Spc72 even in the absence of all three APC/C activators (**Fig EV1B, C**). Thus, the question becomes: is deletion of *AMA1* sufficient to prevent degradation of Spo13? The answer is 'no'. First, Spo13 degradation requires Cdc20 but occurs normally in *ama1Δ* mutants (Sullivan & Morgan, 2000). Second, Ama1 is inactive at anaphase I (Okaz et al., 2012), which is why Ama1-specific substrates (e.g., Cdc5) are stable at this stage. Accordingly, Ama1 is dispensable for MP assembly and PSM formation. However, as mentioned on page 8, *ama1Δ* mutants fail to close the PSM and therefore fail to form spores (Diamond et al., 2009; see **new Fig EV1A and Fig 9B).** While we have confirmed that *ama1Δ* cells are proficient in Spc72 degradation and MP assembly, we prefer to cite the paper of Diamond et al. (2009).

3. Page 11, paragraph 3, "inhibition of Cdc5 activity prevents the removal of Spc72 from SPBs." Page 12, paragraph 2, "inhibition of Ime2 activity prevents the removal of Spc72." Page 13, paragraph 1, "Which expression of either Ime2-ΔC or Cdc5 alone has no effect, co-expression of both kinases causes degradation of removal from SPBs of Spc72." Page 22, paragraph 3, "We have placed Ime2 at the bottom of the signaling cascade from APC/C^{Cdc20} in the nucleus to Spc72 on the cytoplasmic face of the SPB because it is the only meiosis-specific regulator required for reconstituting Spc72 removal in mitotic cells." This seems like a weak reason to place Ime2 at the bottom of the signaling cascade. If there is additional justification to do so, please provide.
The wiring diagram in **Fig 9D** aims to summarize our data in an easily-accessible manner and with a minimum of unknown components. The emphasis is on regulatory properties rather than biochemical mechanisms, many of which remain to be elucidated (discussed on page 24). The network provides a regulatory environment for the function of Spo13 and a testable hypothesis for the regulation of meiosis II-specific processes. We agree that "signaling cascade" implies more mechanistic detail than we currently provide and have abandoned this expression. We also appreciate that placing Cdc5 into the network is challenging because it promotes and also inhibits Spc72's removal from SPBs. Our network provides a simple solution to this conundrum, which is consistent with our data and published work. We are not aware of a substantially different alternative to our network.
Rationale for placing Ime2 in a downstream position: while Cdc5 and Ime2 are required for Spc72 removal (**Fig 5A**), they are dispensable for MP formation once Spc72 has been removed/degraded (**Appendix Fig S6**). This puts Cdc5 and Ime2 on the left arm of the wiring diagram in **Fig 9D**. Since Cdc5 and Ime2 are required for Spc72 removal in *cdc20 ama1Δ spo13Δ* cells (**Fig 5**), they must act downstream of Spo13 and upstream of Spc72. Reconstitution of Spc72 removal in prophase-arrested *ndt80Δ* cells (**Fig 6**) and mitotic cells (**Fig 7 and Fig EV4**) supports this order. Furthermore, we now show that hyperactive Ime2-ΔC causes Spc72 removal in *cdc20 ama1Δ* cells, that is, in cells containing active Spo13 (page 13; **new Appendix Fig S5C**). We think it likely that Ime2 is the downstream component that elicits Spc72 removal. Expression of meiosis-specific Ime2 is sufficient to trigger the meiosis-specific process of Spc72 removal in mitotic cells (**Fig 7**). By contrast, Cdc5 cannot (and, one might argue, should not) induce Spc72 removal on its own, even at very high levels. Nevertheless, considering that Cdc5 binds and potentially activates Ime2, we discuss (page 24) that Spc72 removal/degradation is mediated by a Cdc5-Ime2 complex. However, this does not change the regulatory properties of the network. Ime2 or Cdc5-Ime2 might target a ubiquitin-ligase to Spc72. Since this hypothetical ligase is not essential for the regulatory properties of the network, we have omitted it from **Fig 9D**.
Rationale for Cdc5 activating Ime2's function in Spc72 removal: Cdc5 and Ime2 interact, and they are both required for Spc72's removal in *cdc20 ama1Δ spo13Δ* cells (**Fig 5A, C**). However, Cdc5 but not Ime2 has been shown to bind to SPBs. Furthermore, Cdc5 modifies/phosphorylates Ime2, while we have no evidence for the reverse (**new Fig 5E**). A simple interpretation is that Cdc5 activates Ime2 and recruits it to SPBs. This idea is consistent with published work. Ime2 is activated as cells produce Ndt80, and consequently Cdc5, at entry into metaphase I (Benjamin et al., 2003). Indeed, Schindler and Winter (2006) have proposed Cdc5 as a kinase that activates Ime2.
Rationale for the Cdc5-Spo13 kinase inhibiting Ime2's function in Spc72 removal: the *SPO13* deletion and the *spo13-m2* mutation cause Spc72 removal in *cdc20 ama1Δ* cells, (**Fig 2A**), suggesting that Spo13 prevents Cdc5 from promoting Spc72's removal or that Cdc5-Spo13 inhibits removal and is able to override the function of free Cdc5 in

promoting removal. Our data and previously published results support the latter possibility. First, Spo13 levels are much lower than those of Cdc5 (**Fig 5B**), making it unlikely that Spo13 can inhibit all of Cdc5. Second, Cdc5 activity and Spo13 are both required for inhibiting MP assembly at metaphase I (**Fig 8C, new Fig 8D**), implying that Cdc5-Spo13 is an active kinase. Consistent with this, the monopolin Lrs4 and the cyclin Clb1 are phosphorylated in a Cdc5- and Spo13-dependent manner (see page 25). The proposal that Cdc5-Spo13 inhibits Ime2's function in Spc72 removal is a simple model accommodating all our data. This idea is consistent with the observation of Berchowitz et al. (2013) that Ime2 activity further increases as cells enter meiosis II.

Activation of Spo13 by Cdk1: to test whether Spo13 is activated by Cdk1, we analyzed non-phosphorylatable (*spo13-10A*) and phospho-mimicking mutants (*spo13-10D*) in cells with low Cdk1 activity, namely *ndt80Δ* cells expressing Cdc5 and Ime2 (**Fig 6**). We now provide analysis of the *spo13* phospho-site mutants also in *cdc20 ama1Δ* cells, which contain high Cdk1 activity (**new Fig EV3C, D**). While *spo13-10A* behaves similar to the *SPO13* deletion, *spo13-10D* resembles wild-type *SPO13*, suggesting that phosphorylation by Cdk1 is essential for Spo13's function.

Minor criticisms:

1. Page 14, paragraph 2, "These conditions would limit MP assembly to metaphase II of meiosis but do not exist in a normal mitosis." Should "but" be "because they"? Which would change the sentence to: "These conditions would limit MP assembly to metaphase II of meiosis because they do not exist in a normal mitosis."

We prefer to keep the current expression. The sentence is a prediction from experiments in mitotic cells (**Fig 7**). While it is clear that these conditions do not exist in a normal mitosis (Ime2 is not expressed), it remains to be tested (in the following experiments, i.e., **Fig 8 and Fig 9**) whether the prediction holds true in meiosis.

2. Page 16, paragraph 1, "While Cdk1-Clb1 plays a specific role in Spc72 removal, this does not seem to be the case in MP assembly." This sentence is confusing because it implies that Clb1 does not play a role in MP assembly. It appears instead that Clb1 plays a role in MP assembly but it can be replaced by Clb2, 3 or 4. The authors could clarify, by adding an additional sentence.

We agree with the reviewer and have revised the relevant sentences (page 17). Furthermore, we have performed a new experiment to clarify the roles of APC/C-Cdc20 and Cdk1 in MP assembly and PSM formation at meiosis II. Briefly, we arrested *cdc20^{ts}-mAR ama1 cdh1* cells containing Mpc70-GFP, GFP-tubulin, and RFP-Spo20 at metaphase II and then inhibited analogue-sensitive Cdk1 (**new Fig 9C**). Four conclusions can be drawn: first, recruitment of Mpc70-GFP to SPBs shows that APC/C-Cdc20 activity at meiosis II is dispensable for MP formation, while APC/C-Cdc20 activity at meiosis I is essential (it triggers Spc72 removal). Second, inactivation of Cdk1 causes disassembly of MPs, suggesting that Cdk1 activity is required for formation and persistence of MPs at meiosis II. Third, cells arrested at metaphase II due to inactivation/depletion of APC/C activators do not form PSMs, suggesting that APC/C-Cdc20 activity at meiosis II is required for PSM formation. Importantly, this finding reveals how SAC activity at meiosis II blocks sporulation (page 17, 18). Fourth, inhibition of Cdk1 does not restore PSM formation, suggesting that the relevant Cdc20-substrate is not a cyclin. This substrate is now called "X" in revised **Fig 9D**.

3. General note. There are places in the paper where the meaning becomes convoluted when discussing the absence of a protein or an effect (i.e. when using words such as "lack," "block," "deletion," "prevent," or "removal" back to back). For instance, page 11, paragraph 3, "Indeed, depletion of Cdc5 prevents Spc72's removal also in the spo13Δ mutant." It's possible that this can't be avoided, but it can result in unnecessary confusion.

We agree with the reviewer that the language can be improved at times and have revised some of the relevant sentences. In others, however, we did not find a better expression.

Thank you for submitting your final revised manuscript for our consideration. I am pleased to inform you that following positive re-review by one of the original referees (see comments below), we have now accepted it for publication in The EMBO Journal.

REFEREE REPORTS

Referee #2:

The authors addressed my concerns. I recommend accepting the revised manuscript for publication.

EMBO PRESS

YOU MUST COMPLETE ALL CELLS WITH A PINK BACKGROUND ➔

PLEASE NOTE THAT THIS CHECKLIST WILL BE PUBLISHED ALONGSIDE YOUR PAPER

Corresponding Author Name: Wolfgang Zachariae

Journal Submitted to: EMBO Journal

Manuscript Number: EMBOJ-2021-109446

Reporting Checklist For Life Sciences Articles (Rev. June 2017)

This checklist is used to ensure good reporting standards and to improve the reproducibility of published results. These guidelines are consistent with the Principles and Guidelines for Reporting Preclinical Research issued by the NIH in 2014. Please follow the journal's authorship guidelines in preparing your manuscript.

A- Figures

1. Data

The data shown in figures should satisfy the following conditions:

- ➔ the data were obtained and processed according to the field's best practice and are presented to reflect the results of the experiments in an accurate and unbiased manner.
- ➔ figure panels include only data points, measurements or observations that can be compared to each other in a scientifically meaningful way.
- ➔ graphs include clearly labeled error bars for independent experiments and sample sizes. Unless justified, error bars should not be shown for technical replicates.
- ➔ if n< 5, the individual data points from each experiment should be plotted and any statistical test employed should be justified
- ➔ Source Data should be included to report the data underlying graphs. Please follow the guidelines set out in the author ship guidelines on Data Presentation.

2. Captions

Each figure caption should contain the following information, for each panel where they are relevant:

- ➔ a specification of the experimental system investigated (eg cell line, species name).
- ➔ the assay(s) and method(s) used to carry out the reported observations and measurements
- ➔ an explicit mention of the biological and chemical entity(ies) that are being measured.
- ➔ an explicit mention of the biological and chemical entity(ies) that are altered/varied/perturbed in a controlled manner.

- ➔ the exact sample size (n) for each experimental group/condition, given as a number, not a range;
- ➔ a description of the sample collection allowing the reader to understand whether the samples represent technical or biological replicates (including how many animals, litters, cultures, etc.).
- ➔ a statement of how many times the experiment shown was independently replicated in the laboratory.
- ➔ definitions of statistical methods and measures:
  - common tests, such as t-test (please specify whether paired vs. unpaired), simple χ^2 tests, Wilcoxon and Mann-Whitney tests, can be unambiguously identified by name only, but more complex techniques should be described in the methods section;
  - are tests one-sided or two-sided?
  - are there adjustments for multiple comparisons?
  - exact statistical test results, e.g., P values = x but not P values < x;
  - definition of 'center values' as median or average;
  - definition of error bars as s.d. or s.e.m.

Any descriptions too long for the figure legend should be included in the methods section and/or with the source data.

In the pink boxes below, please ensure that the answers to the following questions are reported in the manuscript itself. Every question should be answered. If the question is not relevant to your research, please write NA (non applicable). We encourage you to include a specific subsection in the methods section for statistics, reagents, animal models and human subjects.

USEFUL LINKS FOR COMPLETING THIS FORM

http://www.antibodypedia.com
http://1degreebio.org
http://www.equator-network.org/reporting-guidelines/improving-bioscience-research-repor
http://grants.nih.gov/grants/olaw/olaw.htm
http://www.mrc.ac.uk/Ourresearch/Ethicsresearchguidance/Useofanimals/index.htm
http://ClinicalTrials.gov
http://www.consort-statement.org
http://www.consort-statement.org/checklists/view/32-consort/66-title
http://www.equator-network.org/reporting-guidelines/reporting-recommendations-for-tumo
http://datadryad.org
http://figshare.com
http://www.ncbi.nlm.nih.gov/gap
http://www.ebi.ac.uk/ega
http://biomodels.net/
http://biomodels.net/miriam/
http://jjj.biochem.sun.ac.za
https://osp.od.nih.gov/biosafety-biosecurity-and-emerging-biotechnology/
http://www.selectagents.gov/

B- Statistics and general methods

Please fill out these boxes ➔ (Do not worry if you cannot see all your text once you press return)

1.a. How was the sample size chosen to ensure adequate power to detect a pre-specified effect size?	We did not pre-specify an effect size.
1.b. For animal studies, include a statement about sample size estimate even if no statistical methods were used.	No animals were used; this study uses budding yeast.
2. Describe inclusion/exclusion criteria if samples or animals were excluded from the analysis. Were the criteria pre-established?	No samples were excluded from analysis.
3. Were any steps taken to minimize the effects of subjective bias when allocating animals/samples to treatment (e.g. randomization procedure)? If yes, please describe.	Randomization procedures were not applied.
For animal studies, include a statement about randomization even if no randomization was used.	No animals were used; this study uses budding yeast.
4.a. Were any steps taken to minimize the effects of subjective bias during group allocation or/and when assessing results (e.g. blinding of the investigator)? If yes please describe.	Blinding was not performed.
4.b. For animal studies, include a statement about blinding even if no blinding was done	No animals were used; this study uses budding yeast.
5. For every figure, are statistical tests justified as appropriate?	Yes, to the best of our knowledge.
Do the data meet the assumptions of the tests (e.g., normal distribution)? Describe any methods used to assess it.	Distributions tend to be symmteric or slightly skewed. Thus, we decided, a priori, to use Welch's t-test to compare group means and provide 95% confidence intervals for the difference between group means. With a balanced design and samples sizes of n = 80-100 per group, Welch's t-test is robust to moderate deviations from normality.
Is there an estimate of variation within each group of data?	An estimate of variation is provided, either in the figures, figure legends, or in the text.

Is the variance similar between the groups that are being statistically compared?	We did not asume the variance to be similar between gropus. We used the t-test with correction for unequal variance (Welch's t-test) to compare group means.

C- Reagents

6. To show that antibodies were profiled for use in the system under study (assay and species), provide a citation, catalog number and/or clone number, supplementary information or reference to an antibody validation profile. e.g., Antibodypedia (see link list at top right), 1DegreeBio (see link list at top right).	Citations and/or supplier and catalog numbers for antibodies are provided.
7. Identify the source of cell lines and report if they were recently authenticated (e.g., by STR profiling) and tested for mycoplasma contamination.	A table with the full genotypes of all Saccharomyces cerevisiae strains used in this study is provided.

* for all hyperlinks, please see the table at the top right of the document

D- Animal Models

8. Report species, strain, gender, age of animals and genetic modification status where applicable. Please detail housing and husbandry conditions and the source of animals.	NA
9. For experiments involving live vertebrates, include a statement of compliance with ethical regulations and identify the committee(s) approving the experiments.	NA
10. We recommend consulting the ARRIVE guidelines (see link list at top right) (PLoS Biol. 8(6), e1000412, 2010) to ensure that other relevant aspects of animal studies are adequately reported. See author guidelines, under 'Reporting Guidelines'. See also: NIH (see link list at top right) and MRC (see link list at top right) recommendations. Please confirm compliance.	NA

E- Human Subjects

11. Identify the committee(s) approving the study protocol.	NA
12. Include a statement confirming that informed consent was obtained from all subjects and that the experiments conformed to the principles set out in the WMA Declaration of Helsinki and the Department of Health and Human Services Belmont Report.	NA
13. For publication of patient photos, include a statement confirming that consent to publish was obtained.	NA
14. Report any restrictions on the availability (and/or on the use) of human data or samples.	NA
15. Report the clinical trial registration number (at ClinicalTrials.gov or equivalent), where applicable.	NA
16. For phase II and III randomized controlled trials, please refer to the CONSORT flow diagram (see link list at top right) and submit the CONSORT checklist (see link list at top right) with your submission. See author guidelines, under 'Reporting Guidelines'. Please confirm you have submitted this list.	NA
17. For tumor marker prognostic studies, we recommend that you follow the REMARK reporting guidelines (see link list at top right). See author guidelines, under 'Reporting Guidelines'. Please confirm you have followed these guidelines.	NA

F- Data Accessibility

18: Provide a "Data Availability" section at the end of the Materials & Methods, listing the accession codes for data generated in this study and deposited in a public database (e.g. RNA-Seq data: Gene Expression Omnibus GSE39462, Proteomics data: PRIDE PXD000208 etc.) Please refer to our author guidelines for 'Data Deposition'.<br><br>Data deposition in a public repository is mandatory for:<br>a. Protein, DNA and RNA sequences<br>b. Macromolecular structures<br>c. Crystallographic data for small molecules<br>d. Functional genomics data<br>e. Proteomics and molecular interactions	Under "Data Availability", we state that no datasets were deposited in public repositories.
19. Deposition is strongly recommended for any datasets that are central and integral to the study; please consider the journal's data policy. If no structured public repository exists for a given data type, we encourage the provision of datasets in the manuscript as a Supplementary Document (see author guidelines under 'Expanded View' or in unstructured repositories such as Dryad (see link list at top right) or Figshare (see link list at top right).	NA
20. Access to human clinical and genomic datasets should be provided with as few restrictions as possible while respecting ethical obligations to the patients and relevant medical and legal issues. If practically possible and compatible with the individual consent agreement used in the study, such data should be deposited in one of the major public access-controlled repositories such as dbGAP (see link list at top right) or EGA (see link list at top right).	NA
21. Computational models that are central and integral to a study should be shared without restrictions and provided in a machine-readable form. The relevant accession numbers or links should be provided. When possible, standardized format (SBML, CellML) should be used instead of scripts (e.g. MATLAB). Authors are strongly encouraged to follow the MIRIAM guidelines (see link list at top right) and deposit their model in a public database such as Biomodels (see link list at top right) or JWS Online (see link list at top right). If computer source code is provided with the paper, it should be deposited in a public repository or included in supplementary information.	NA

G- Dual use research of concern

22. Could your study fall under dual use research restrictions? Please check biosecurity documents (see link list at top right) and list of select agents and toxins (APHIS/CDC) (see link list at top right). According to our biosecurity guidelines, provide a statement only if it could.	No.
